# A phase 1 trial of lipid-encapsulated mRNA encoding a monoclonal antibody with neutralizing activity against Chikungunya virus

**DOI:** 10.1038/s41591-021-01573-6

**Published:** 2021-12-09

**Authors:** Allison August, Husain Z. Attarwala, Sunny Himansu, Shiva Kalidindi, Sophia Lu, Rolando Pajon, Shu Han, Jean-Michel Lecerf, Joanne E. Tomassini, Marjie Hard, Leon M. Ptaszek, James E. Crowe, Tal Zaks

**Affiliations:** 1 Moderna, Inc., Cambridge, MA USA; 2 Massachusetts General Hospital, Boston, MA USA; 3 Vanderbilt University Medical Center, Nashville, TN USA

**Keywords:** Infectious diseases, Viral infection, Preventive medicine

## Abstract

Chikungunya virus (CHIKV) infection causes acute disease characterized by fever, rash and arthralgia, which progresses to severe and chronic arthritis in up to 50% of patients. Moreover, CHIKV infection can be fatal in infants or immunocompromised individuals and has no approved therapy or prevention. This phase 1, first-in-human, randomized, placebo-controlled, proof-of-concept trial conducted from January 2019 to June 2020 evaluated the safety and pharmacology of mRNA-1944, a lipid nanoparticle-encapsulated messenger RNA encoding the heavy and light chains of a CHIKV-specific monoclonal neutralizing antibody, CHKV-24 (NCT03829384). The primary outcome was to evaluate the safety and tolerability of escalating doses of mRNA-1944 administered via intravenous infusion in healthy participants aged 18–50 years. The secondary objectives included determination of the pharmacokinetics of mRNA encoding for CHKV-24 immunoglobulin heavy and light chains and ionizable amino lipid component and the pharmacodynamics of mRNA-1944 as assessed by serum concentrations of mRNA encoding for CHKV-24 immunoglobulin G (IgG), plasma concentrations of ionizable amino lipid and serum concentrations of CHKV-24 IgG. Here we report the results of a prespecified interim analysis of 38 healthy participants who received intravenous single doses of mRNA-1944 or placebo at 0.1, 0.3 and 0.6 mg kg^−1^, or two weekly doses at 0.3 mg kg^−1^. At 12, 24 and 48 h after single infusions, dose-dependent levels of CHKV-24 IgG with neutralizing activity were observed at titers predicted to be therapeutically relevant concentrations (≥1 µg ml^−1^) across doses that persisted for ≥16 weeks at 0.3 and 0.6 mg kg^−1^ (mean t_1/2_ approximately 69 d). A second 0.3 mg kg^−1^ dose 1 week after the first increased CHKV-24 IgG levels 1.8-fold. Adverse effects were mild to moderate in severity, did not worsen with a second mRNA-1944 dose and none were serious. To our knowledge, mRNA-1944 is the first mRNA-encoded monoclonal antibody showing in vivo expression and detectable ex vivo neutralizing activity in a clinical trial and may offer a treatment option for CHIKV infection. Further evaluation of the potential therapeutic use of mRNA-1944 in clinical trials for the treatment of CHIKV infection is warranted.

## Abstract (web summary)

An mRNA-based therapeutic can drive expression of a functional antibody in humans at levels capable of neutralizing Chikungunya virus ex vivo.

## Main

CHIKV is a positive-sense, single-stranded RNA virus in the alphavirus family and is transmitted by *Aedes* mosquitoes. Infection with CHIKV causes an acute disease that can progress to severe and chronic arthritis in up to 50% of patients and can be fatal in some populations, including infants and immunocompromised individuals^1–6^. Although historically CHIKV infection has been an endemic pathogen limited to Africa and Asia, more recently outbreaks and epidemics have occurred in other regions due to the increasing distribution of the *Aedes* vector. In 2004, a CHIKV epidemic in Kenya spread rapidly across the Indian Ocean and India, attributed to spread by viremic travelers, leading to millions of infected individuals worldwide, including in countries of Europe and the Americas^6^. In 2015, in the Americas, 693,489 suspected cases of CHIKV were reported to the Pan American Health Organization^7^. In 2016, Brazil, Bolivia and Colombia reported the most cases worldwide, followed by India, Kenya and Argentina^7,8^. In 2017, cases were reported in ten European countries, with more than half occurring in Italy; overall there were >1 million cases of CHIKV infection reported in the Americas and an estimated 3 million cases globally^7,8^.

Currently, there is no licensed vaccine nor antiviral drug available to prevent CHIKV infection. Several approaches are in development including various vaccine and passive immunization strategies based on the known roles of humoral and cellular immunity toward the protection and elimination of CHIKV infection^1–3,6,9–12^. Defining a correlate of protection for the implementation of clinical trials in the evaluation of vaccines and therapeutics for CHIKV is important because this virus causes large epidemics that are followed by a rapid decline in new infections due to herd immunity. Studies in small animals and nonhuman primates suggest that neutralizing antibodies are a potential immune correlate of protection for CHIKV^13–16^. CHIKV infection induces high levels of neutralizing antibodies in humans that mainly recognize the envelope glycoprotein (E2) and correlate with viral clearance and protection across genotypes. There is also evidence for the long-term persistence of CHIKV neutralizing antibodies, which remain protective for years in previously infected individuals^17–19^. Passive immunotherapy with convalescent sera from CHIKV-infected patients or human CHIKV-specific neutralizing human monoclonal antibodies that mainly recognize the E2 glycoprotein, also prevent CHIKV infection in animal models, providing an additional possible approach for the prevention of infection and disease^20–24^. In humans, epidemiological studies identified levels of pre-existing CHIKV neutralizing antibodies associated with a decreased risk of symptomatic CHIKV infection and correlated with those in convalescent patient sera^25,26^.

Monoclonal antibody therapies have been approved clinically for frontline prevention or treatment of several infectious diseases including respiratory syncytial virus, Zaire Ebola virus, human immunodeficiency virus and inhalational anthrax^27–30^. Similar therapies for the treatment of severe acute respiratory syndrome coronavirus 2 (SARS-CoV-2) infection are being actively studied for their potential to provide passive immunity as both prophylaxis in susceptible populations and strategies for treatment of active infections^31,32^. However, the development and widespread use of monoclonal antibody therapeutics has been impeded by subtherapeutic neutralizing effects in some cases, production challenges and high costs associated with the manufacture of recombinant antibody proteins^33–35^.

Alternative methods to produce effective, safe and cost-effective monoclonal antibody therapies are being pursued, including those based on DNA and RNA delivery, which can circumvent the purification process and posttranslational modification concerns related to recombinant antibody technology^33^. mRNA platforms have been used to generate monoclonal antibodies in preclinical studies^36–38^ and antigens that elicit immune responses in early-phase and advanced vaccine clinical trials^39–45^. The efficacy of mRNA platforms in eliciting binding and neutralizing antibodies has also been shown in recent mRNA vaccine trials for SARS-CoV-2 (refs. ^46–48^). Moreover, mRNA sequence modification affords the opportunity to engineer both antigen and antibody modifications, including antibody constant region (Fc) alterations that extend half-life, reduce innate immune activation, optimize effector function or eliminate Fc receptor interactions potentially related to antibody-dependent enhancement of pathogenesis and enhance antiviral potencies^33,49–53^.

We recently reported that a lipid nanoparticle (LNP)-encapsulated mRNA encoding the light and heavy chains of a human monoclonal antibody (CHKV-24 IgG) targeting the CHIKV E2 glycoprotein elicited high levels of biologically relevant neutralizing antibodies when administered by infusion into mice or cynomolgus macaques^54^. The antibody protected against arthritis, musculoskeletal disease and lethal challenge in mice infected with CHIKV. Infusion of the mRNA-encoding CHKV-24 IgG in cynomolgus macaques was well tolerated with mean maximum serum concentrations of human CHKV-24 IgG antibodies (10.1–35.9 µg ml^−1^) reached at 24 h that exceeded the levels needed for protection in mice (3–10 µg ml^−1^) and were detectable through day 83 after a single dose at 0.5 mg kg^−1^ and through day 100 after 2 weekly doses of 3 mg kg^−1^ (ref. ^54^). The antibody levels achieved in these preclinical studies, combined with evidence for the protective roles of CHIKV neutralizing antibody in animal models and seroepidemiological studies in humans, suggest that serum levels of CHKV-24 IgG ≥1 µg ml^−1^ represent an initial target anticipated to correlate with neutralizing activity to inform vaccine and therapeutic antibody development^13,25,26^.

These preclinical data support further assessment of the clinical potential of this LNP mRNA (mRNA-1944). In this report, we present the results of the interim analysis of a phase 1, first-in-human, randomized, placebo-controlled, proof-of-concept trial of the safety, tolerability, pharmacokinetics (PK) and pharmacodynamics (PD) of mRNA-1944 in healthy adults.

## Results

### Trial population

**Fig. 1:**
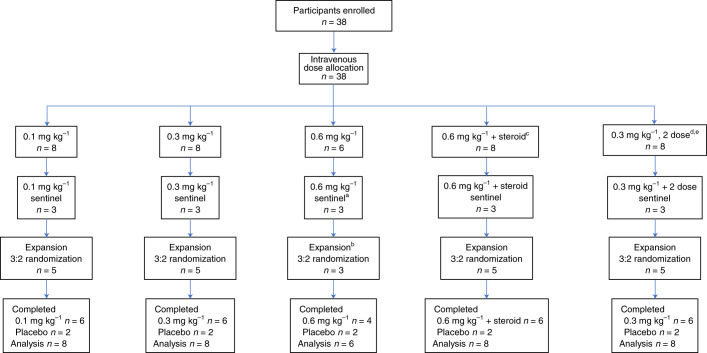
Trial flow. The study protocol planned to enroll 8 participants per treatment group. Six of 8 participants were allocated in the 0.6 mg kg^−1^ dose group due to the decision to augment the premedication regimen with steroid to assess its potential mitigation of infusion-related reactions (0.6 mg kg^−1^ + steroid group). ^a^Participants received loratadine and ranitidine 90 min before infusion. ^b^Participants received loratadine, ranitidine (sentinel, expansion) and acetaminophen (expansion) 90 min before infusion. ^c^Participants received steroid (dexamethasone) and diphenhydramine and famotidine 90 min before infusion. ^d^Participants received diphenhydramine and famotidine 90 min before infusion. ^e^Participants were administered two 0.3 mg kg^−1^ doses on days 1 and 8.

Between 10 January 2019 and 1 June 2020, 38 participants were enrolled in the study. All were considered eligible. Between 22 January 2019 and 18 June 2020, all 38 were randomized to treatment with a single dose of mRNA-1944 at 0.1 mg kg^−1^, 0.3 mg kg^−1^, 0.6 mg kg^−1^ or 0.6 mg kg^−1^ preadministered with a steroid to assess its impact on infusion-related reactions (0.6 mg kg^−1^ with a steroid) or placebo, or 2 weekly doses at 0.3 mg kg^−1^ or placebo administered on days 1 and 8 (Fig. 1). All 38 participants received the intended treatment at the study site and were observed for 48 h afterwards as inpatients. Participants were followed for safety, PK and PD analyses for at least eight weeks postdose (data cutoff date: 15 October 2020) during the prespecified interim analysis. Per protocol, the follow-up of study participants for safety, PK and PD will continue for 12 months postdose. The follow-up data will be summarized in a subsequent report.

The baseline characteristics of participants were generally balanced between treatment groups (Table 1). The mean age of participants was 35 years, 53% were female and most participants (74%) were white. Participants had a mean body weight of 76 kg and a body mass index (BMI) of 27.1 kg m^−2^.

**Table 1:** Demographic and baseline characteristics of the study participants

	mRNA-1944	mRNA-1944	mRNA-1944	mRNA-1944	mRNA-1944	mRNA-1944	Placebo	Overall
	0.1 mg kg^−1^^a^	0.3 mg kg^−1^^a^	0.6 mg kg^−1^^b^	0.6 mg kg^−1^ + steroid^c^	0.3 mg kg^−1^ 2-dose^d,e^	All	All	
	*n* = 6	*n* = 6	*n* = 4	*n* = 6	*n* = 6	*n* = 28	*n* = 10	*n* = 38
Age, years mean (range)	34.0 (20–43)	37.5 (32–50)	36.3 (23–49)	36.2 (24–49)	34.7 (20–46)	35.7 (20–50)	33.2 (24–46)	35.0 (20–50)
Women, *n* (%)	3 (50.0)	4 (66.7)	1 (25.0)	5 (83.3)	2 (33.3)	15 (53.6)	5 (50.0)	20 (52.6)
Race, *n* (%)								
White	4 (66.7)	5 (83.3)	2 (50.0)	6 (100)	3 (50.0)	20 (71.4)	8 (80.0)	28 (73.7)
Black or African American	2 (33.3)	1 (16.7)	1 (25.0)	0	3 (50.0)	7 (25.0)	1 (10.0)	8 (21.1)
Other	0	0	1 (25.0)	0	0	1 (3.6)	1 (10.0)	2 (5.3)
Weight, mean kg	85. 8	78.8	85.3	69.6	68.2	77.0	73.2	76.0
BMI, mean kg m^−^^2^	29.9	29.6	30.3	24.8	25.6	27.9	25.0	27.1

Percentages are based on the number of participants in the safety population who received the specified treatment and the total number in each group. ^a^Participants received loratadine and ranitidine 90 min before infusion. ^b^Participants received loratadine, ranitidine (sentinel, expansion) and acetaminophen (expansion) 90 min before infusion. ^c^Participants received steroid (dexamethasone) and diphenhydramine and famotidine 90 min before infusion. ^d^Participants received diphenhydramine and famotidine 90 min before infusion. ^e^Participants were administered two 0.3 mg kg^−1^ doses on days 1 and 8.

### Safety

Safety was assessed in the safety population, which included all 38 participants who received at least 1 dose of mRNA-1944 or placebo treatment. Adverse events (AEs) were graded according to the National Cancer Institute Common Terminology Criteria for Adverse Events (CTCAE) v.5^55^. Overall, 19 (50%) participants reported treatment-related AEs (Table 2), nearly all of which were attributed to infusion-related reactions (18 (95%)). All AEs were transient and resolved spontaneously without treatment except for one episode of emesis for which the participant received ondansetron. There were no deaths, serious AEs or discontinuations due to AEs. The most common treatment-related AEs occurring at a frequency of >5% in the study across the mRNA-1944 groups were headache, nausea and myalgia; chills, fatigue and nausea occurred in those on placebo. The highest frequencies of treatment-related AEs were reported in the 0.6 mg kg^−1^ groups with and without steroid and in the 0.3 mg kg^−1^ group combined for 2 doses. Few treatment-related AEs occurred in the single-dose 0.1 and 0.3 mg kg^−1^ mRNA-1944 and placebo groups.

**Table 2:** Treatment-related AEs

	mRNA-1944	mRNA-1944	mRNA-1944	mRNA-1944	mRNA-1944	mRNA-1944	Placebo
	0.1 mg kg^−1^^a^	0.3 mg kg^−1^^a^	0.6 mg kg^−1^^b^	0.6 mg kg^−1^ + steroid^c^	0.3 mg kg^−1^ 2-dose^d,e^	All	All
AE, *n* (%)	*n* = 6	*n* = 6	*n* = 4	*n* = 6	*n* = 6	*n* = 28	*n* = 10
Any	1 (16.7)	1 (16.7)	4 (100.0)	6 (100.0)	5 (83.3)	17 (60.7)	2 (20.0)
Grade 1	1 (16.7)	1 (16.7)	2 (50.0)	4 (66.7)	2 (33.3)	10 (35.7)	2 (20.0)
Grade 2	0	0	1 (25.0)	2 (33.3)	3 (50.0)	6 (21.4)	0
Grade 3	0	0	1 (25.0)^f^	0	0	1 (3.6)	0
Grade 4	0	0	0	0	0	0	0
Serious	0	0	0	0	0	0	0
Leading to death	0	0	0	0	0	0	0
Leading to discontinuation	0	0	0	0	0	0	0
**Occurring in** >**5% of participant SOC, preferred term, CTCAE**^**g**^							
Headache	0	1 (16.7)	3 (75.0)	4 (66.7)	1 (16.7)	9 (32.1)	0
Nausea	0	0	2 (50.0)	2 (33.3)	3 (50.0)	7 (25.0)	1 (10.0)
Myalgia	0	0	0	4 (66.7)	1 (16.7)	5 (17.9)	0
Dizziness	0	0	2 (50.0)	1 (16.7)	1 (16.7)	4 (14.3)	0
Chills	0	0	2 (50.0)	1 (16.7)	1 (16.7)	4 (14.3)	1 (10.0)
Vomiting	0	0	2 (50.0)	0	2 (33.3)	4 (14.3)	0
Flushing	0	0	1 (25.0)	1 (16.7)	2 (33.3)	4 (14.3)	0
Increased heart rate	0	0	0	3 (50.0)	0	3 (10.7)	0
Pyrexia	0	0	2 (50.0)	0	0	2 (7.1)	0
Back pain	0	0	0	1 (16.7)	1 (16.7)	2 (7.1)	0
Musculoskeletal stiffness	0	0	2 (50.0)	0	0	2 (7.1)	0
Increased white blood cell count	0	0	1 (25.0)	1 (16.7)	0	2 (7.1)	0
Sinus tachycardia	0	0	2 (50.0)	0	0	2 (7.1)	0
Decreased appetite	0	0	0	0	2 (33.3)	2 (7.1)	0
Hyperhidrosis	0	0	0	1 (16.7)	1 (16.7)	2 (7.1)	0
Fatigue	0	0	0	0	1 (16.7)	1 (3.6)	1 (10.0)

*n* = number of participants randomized and exposed to the specified study treatment. Treatment-emergent, treatment-related AEs were defined as any event not present before exposure to the study treatment or any event already present that worsened in intensity or frequency after exposure and was considered treatment-related by the investigator. In each group, a participant was counted once if the participant reported one or more AEs. Percentages are based on the number of participants in the safety population who received the specified treatment and total (*n*) in the group. AEs were coded using the Medical Dictionary for Regulatory Activities v.23.0 (grade 1, mild; grade 2, moderate; grade 3, severe; grade 4, life-threatening). ^a^Participants received loratadine and ranitidine 90 min before infusion. ^b^Participants received loratadine, ranitidine (sentinel, expansion) and acetaminophen (expansion) 90 min before infusion. ^c^Participants received steroid (dexamethasone) and diphenhydramine and famotidine 90 min before infusion. ^d^Participants received diphenhydramine and famotidine 90 min before infusion. ^e^Participants were administered two 0.3 mg kg^−1^ doses on days 1 and 8. ^f^One participant had 2 grade 3 AEs (one of increased white blood cell count and one of sinus tachycardia). ^g^AEs are listed in order of decreasing frequency of treatment-related AEs >5% for the active treatment group. SOC, system organ class.

Treatment-related AEs were generally mild to moderate in severity (Table 2 and Supplementary Table 1), most of which were grade 1 events. Six (21.4%) grade 2 events and 1 (3.6%) grade 3 event were reported for the 28 participants across the mRNA-1944 groups. In the 0.6 mg kg^−1^ group (without steroid), the most frequent grade 2 events were nausea and vomiting (2 (50.0%) of 4 participants for each) and there was also 1 grade 3 event of sinus tachycardia that started 1 h postdose and resolved without intervention by 16 h postdose. In the 0.6 mg kg^−1^ preadministered steroid group, 1 grade 2 event of headache was reported. In the 0.3 mg kg^−1^ 2-dose group, the most frequently reported grade 2 events were nausea in 1 (17%) and vomiting in 2 (33%) of 6 participants after the first dose and retching in 1 (17%) after the second dose (Supplementary Table 2). No grade 3 events occurred at either dose in this group, nor was there an increase in the percentage of participants who reported any AE with the second dose compared with the first dose. There were no exacerbations or worsening of symptoms in any participant after the second dose compared with dose 1. Also, the rate and severity distribution of AEs was lower than those reported in the 0.6 mg kg^−1^ groups and the overall AE profile was similar to that of the other dose groups.

Changes from baseline for all laboratory assessments were generally minimal and similar between all treatment and placebo groups including both doses in the 0.3 mg kg^−1^ 2-dose regimen with no meaningful changes in liver or kidney laboratory results. One grade 3 AE of increased white blood cell count, which returned to normal 5 d postdose, was observed in the 0.6 mg kg^−1^ group.

### Serum levels of CHKV-24 IgG

**Fig. 2:**
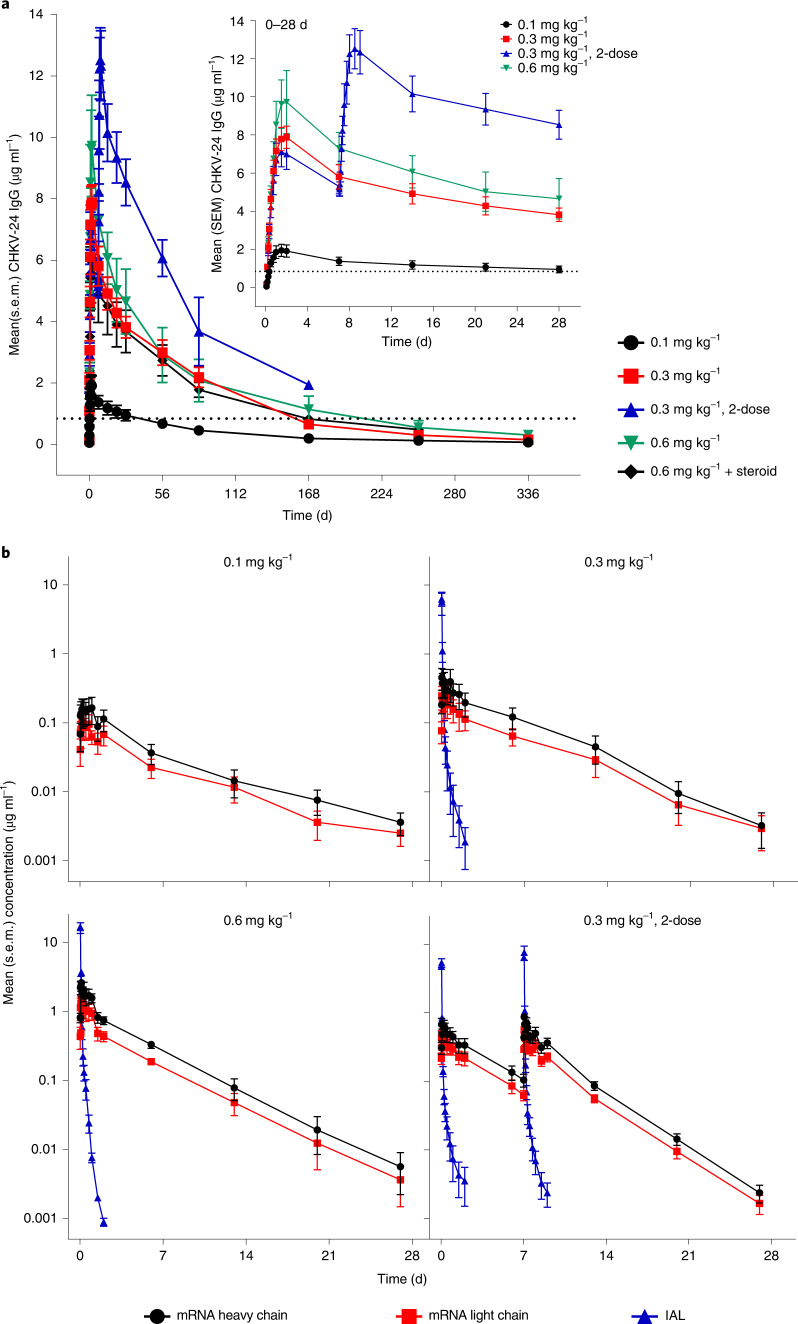
Serum concentration of CHKV-24 IgG, mRNA encoding heavy and light chains of CHKV-24 IgG and IAL. **a**, Mean serum concentration time profiles of CHKV-24 IgG after the administration of single doses of 0.1, 0.3 and 0.6 mg kg^−1^ and 2 doses of 0.3 mg kg^−1^ mRNA-1944 over the course of 366 d and during 28 d (inset). **b**, Mean serum concentration time profiles for mRNA (heavy and light chains) and IAL during 28 d. The error bars represent the s.e.m. The dotted lines represent the serum target concentration of 1 µg ml^−1^ antibody anticipated to provide neutralizing antibody protection against CHIKV infection^13,25,26,54^. *n* = 6 participants at each time point for the single-dose 0.1, 0.3 and 0.6 mg kg^−1^ + steroid and 2-dose 0.3 mg kg^−1^ groups; *n* = 4 participants at each time point for the single-dose 0.6 mg kg^−1^ group examined over 366 d for CHKV-24 IgG and over 28 d for mRNA (heavy and light chains) and IAL.

Serum levels of CHKV-24 IgG were assessed in the PK population, consisting of participants in the safety population who had had detectable serum mRNA encoding for CHKV-24 IgG, which included 28 participants on active treatment. The administration of mRNA-1944 resulted in dose-related increases in CHKV-24 IgG serum levels at the 0.1, 0.3 and 0.6 mg kg^−1^ doses after a single administration (maximum observed effect (E_max_) = 2.0–10.2 µg ml^−1^; Fig. 2a, Table 3 and Supplementary Table 3). At these doses, CHKV-24 IgG reached peak levels within 36–48 h postdose followed by a decline with an overall mean terminal half-life (t_1/2_) of approximately 69 d. Peak levels of CHKV-24 IgG at all dose levels exceeded 1 µg ml^−1^, the lowest effective concentration anticipated to correlate with neutralizing activity^13,25,26,54^; at doses ≥0.3 mg kg^−1^, concentrations of CHKV-24 IgG ≥1 µg ml^−1^ were present for at least 16 weeks post-single dose. Steroid premedication at the 0.6 mg kg^−1^ level led to a slightly lower (approximately 1.7-fold) CHKV-24 IgG E_max_ (6.1 µg ml^−1^) compared with the 0.6 mg kg^−1^ group without steroid premedication (10.2 µg ml^−1^; Supplementary Fig. 1a, Table 3 and Supplementary Table 3). Serum exposures for CHKV-24 IgG were consistent for the single dose administered in the 0.3 mg kg^−1^ group (7.9 µg ml^−1^) and the first dose (7.2 µg ml^−1^) in the 0.3 mg kg^−1^ 2-dose group. Administration of a second weekly mRNA-1944 dose at 0.3 mg kg^−1^ was associated with a 1.8-fold linear accumulation of CHKV-24 IgG concentrations (E_max_ = 12.9 µg ml^−1^), consistent with the extended terminal t_1/2_ of CHKV-24 IgG.

**Table 3:** PK and PD parameters for CHKV-24 IgG mRNA encoding the heavy and light chains of CHKV-24 IgG and IAL

	mRNA-1944	mRNA-1944	mRNA-1944	mRNA-1944	mRNA-1944	mRNA-1944
	0.1 mg kg^−1^^a^	0.3 mg kg^−1^^a^	0.6 mg kg^−1^^b^	0.6 mg kg^−1^ + steroid^c^	0.3 mg kg^−1^ 2-dose^d,e^ 1st dose	0.3 mg kg^−1^ 2-dose^d,e^ 2nd dose
Parameter	*n* = 6	*n* = 6	*n* = 4	*n* = 6	*n* = 6	*n* = 6
**CHKV-24 IgG**						
E_max_, μg ml^−1^ mean (CV%)	2.0 (40.4)	7.9 (18.2)	10.2 (29.6)	6.1 (47.0)	7.2 (26.9)	12.9 (21.4)
TE_max_, h median (range)	36.0 (24.0–48.0)	48.0 (36.0–48.1)	48.0 (36.0–48.0)	48.0 (24.0–48.0)	36.0 (24.0–48.0)	42.0 (24.0–48.0)
AUEC_0–168_, μg h ml^−1^ mean (CV%)	260 (40.0)	1,070 (21.9)	1,310 (20.0)	846 (48.2)	965 (28.3)	1,830 (21.9)
AUEC_0–inf_, μg h ml^−1^ mean (CV%)	2,820 (58.6)	11,600 (24.6)	13,400 (58.1)	11,200 (30.0)	NA	23,400 (26.9)
t_1/2,_ h mean (CV%)	1,470 (34.6)	1,930 (15.4)	1,570 (48.8)	1,720 (24.1)	NA	1,520 (20.9)
**mRNA light chain**						
C_max,_ μg ml^−1^ mean (CV%)	0.12 (70.7)	0.29 (89.9)	1.5 (44.4)	0.90 (48.5)	0.53 (45.6)	0.58 (30.3)
t_max_, h median (range)	12.1 (4.0–48.0)	3.5 (1.0–18.0)	2.0 (1.0–8.0)	2.0 (2.0–4.0)	4.0 (1.0–6.0)	2.0 (1.0–6.0)
AUC_0–168_, μg h ml^−1^ mean (CV%)	8.0 (75.6)	17.7 (77.8)	70.4 (27.6)	49.4 (35.8)	32.2 (42.6)	26.9 (30.3)
AUC_0–inf_, μg h ml^−1^ mean (CV%)	11.6 (77.4)	27.2 (73.6)	89.9 (18.4)	60.4 (33.8)	37.9 (43.8)	NA
t_1/2,_ h mean (CV%)	87.4 (52.6)	88.8 (31.5)	82.5 (35.9)	68.6 (30.9)	62.6 (9.4)	61.2 (22.9)
Clearance, ml h kg^−1^ mean (CV%)	4.6 (69.6)	7.4 (99.4)	2.3 (16.0)	3.8 (51.6)	3.1 (45.9)	3.4 (22.5)
**mRNA heavy chain**						
C_max,_ μg ml^−1^ mean (CV%)	0.21 (78.4)	0.50 (86.6)	2.7 (32.1)	1.5 (53.7)	0.80 (45.5)	0.92 (29.5)
t_max_, h median (range)	18.0 (2.0–24.0)	1.0 (1.0–18.0)	2.0 (1.0–8.0)	4.0 (2.0–24.0)	4.0 (1.0–6.0)	1.5 (1.0–6.0)
AUC_0–168_, μg h ml^−1^ mean (CV%)	13.4 (85.2)	31.3 (87.1)	121.0 (15.4)	84.9 (39.6)	49.8 (46.5)	42.0 (34.3)
AUC_0–inf_, μg h ml^−1^ mean (CV%)	18.9 (83.7)	46.5 (80.0)	154.0 (10.9)	102.0 (35.2)	60.1 (49.3)	NA
t_1/2,_ h mean (CV%)	85.9 (51.9)	85.0 (27.4)	79.6 (31.0)	67.2 (30.2)	64.8 (9.7)	60.7 (22.5)
Clearance ml h kg^−1^ mean (CV%)	5.8 (70.2)	10.2 (106.4)	2.6 (10.8)	4.6 (54.9)	4.1 (52.2)	4.4 (24.9)
**IAL**						
C_max_, μg ml^−1^ mean (CV%)	NA	7.1 (37.1)	15.3 (51.2)	16.3 (28.3)	5.8 (22.2)	8.0 (50.9)
t_max_, h median (range)	NA	1.0 (0.5–1.1)	1.0 (0.5–3.0)	1.00 (0.5–1.0)	1.00 (0.5–1.0)	1.00 (0.5–1.0)
AUC_0–168_, μg h ml^−1^ mean (CV%)	NA	9.5 (43.4)	20.7 (24.3)	28.7 (31.7)	7.8 (15.6)	10.1 (40.1)
AUC_0–inf_, μg h ml^−1^ mean (CV%)	NA	9.5 (43.4)	20.7 (24.3)	28.7 (31.8)	7.8 (15.6)	NA
t_1/2_, h mean (CV%)	NA	8.0 (39.3)	6.7 (33.9)	13.3 (25.7)	8.1 (40.1)	9.8 (26.8)
Clearance, ml h kg^−1^ mean (CV%)	NA	369 (43.8)	313 (30.8)	232 (30.7)	404 (20.0)	334 (29.5)

AUEC_0–168_, area under the effect curve from time 0 to 168 d. ^a^Participants received loratadine and ranitidine 90 min before infusion. ^b^Participants received loratadine, ranitidine (sentinel, expansion) and acetaminophen (expansion) 90 min before infusion. ^c^Participants received steroid (dexamethasone) and diphenhydramine and famotidine 90 min before infusion. ^d^Participants received diphenhydramine and famotidine 90 min before infusion. ^e^Participants were administered two 0.3 mg kg^−1^ doses on days 1 and 8. NA, not assessed.

### Levels of CHKV-24 mRNA and ionizable amino lipid component

Serum levels of mRNAs encoding of CHKV-24 IgG and an ionizable amino lipid (IAL) component were assessed in 28 participants in the PD analysis set that comprised those in the safety population who had both detectable mRNA encoding for each of the heavy and light chains of CHKV-24 IgG and IAL component concentrations. The serum concentrations of the mRNAs encoding the heavy and light chains increased in a dose-proportional manner after single administration of 0.1, 0.3 and 0.6 mg kg^−1^ doses. Peak concentrations were observed during the first 1–18 h after the end of intravenous infusion with maximum serum concentration (C_max_) values ranging from 0.12 to 1.5 µg ml^−1^ (Fig. 2b, Table 3 and Supplementary Table 3). Serum concentrations of mRNA encoding the heavy and light chains of CHKV-24 IgG declined rapidly after reaching peak levels with a terminal t_1/2_ of 83.5 h (range 46.4–126) and 86.2 h (range 46.8–134.7), respectively. As observed for the serum levels of CHKV-24 IgG, mRNA heavy and light chain C_max_ concentrations were approximately 1.7-fold lower in the 0.6 mg kg^−1^ steroid premedication group (Supplementary Fig. 1b, Table 3 and Supplementary Table 3). The results for mRNAs encoding the heavy and light chains of CHKV-24 IgG in the 0.3 mg kg^−1^ 2-dose group were comparable to those in the single-dose 0.3 mg kg^−1^ group and were similar after the first and second doses of treatment. The C_max_ values for the heavy and light chains, respectively were 0.80 and 0.53 µg ml^−1^ after the first dose and 0.92 and 0.58 µg ml^−1^ after the second dose. The serum exposure ratios of the heavy-to-light chain mRNAs were consistent across doses. Likewise, mean t_1/2_ values were comparable at the first (64.8 (range 58.0–74.9) and 62.6 (56.9-71.2)) and second (60.7 (43.9-77.6) and 61.2 (44.3-78.3)) doses for the mRNA heavy and light chains, respectively. Clearance rates for the mRNA heavy and light chains ranged from 2.3 to 10.2 ml h kg^−1^ across all dose groups.

The levels of the IAL component in the serum were evaluated in all dose groups except the 0.1 mg kg^−1^ group. The maximum serum concentrations of IAL were observed at the end of intravenous infusion, which declined rapidly to undetectable levels beyond 48 h postdose. The mean terminal t_1/2_ was 7.3 h (range 4.5–9.6) for the single 0.3 mg kg^−1^ and 0.6 mg kg^−1^ doses (Fig. 2b, and Table 3 and Supplementary Table 3). The C_max_ values for IAL increased dose-proportionately with mean values of 7.1 and 15.3 µg ml^−1^ for the 0.3 and 0.6 mg kg^−1^ single-dose groups, respectively. Serum IAL exposures (C_max_ and area under the concentration curve from time 0 to 168 d (AUC_0–168_)) were similar between the groups receiving a single infusion of 0.6 mg kg^−1^ of mRNA-1944 with or without steroid premedication (Supplementary Fig. 1b, Table 3 and Supplementary Table 3). In the 0.3 mg kg^−1^ 2-dose group, serum IAL exposures postinfusion were similar at the first (AUC_0–168_ = 7.8 h µg ml^−1^) and second (AUC_0–168_ = 10.1 h µg ml^−1^) dose indicating that no substantial accumulation of IAL was observed after the second weekly dose.

### Serum neutralizing antibody titers

**Fig. 3:**
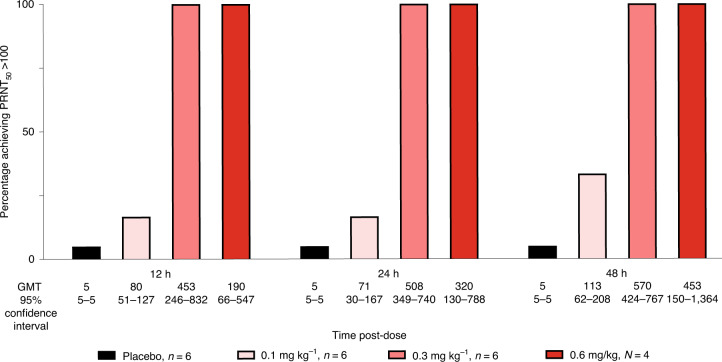
Neutralizing antibody titers for single-dose groups. Serum neutralizing titers of CHKV-24 IgG against CHIKV were assessed using the PRNT at 12, 24 and 48 h after administration of 0.1, 0.3 and 0.6 mg kg^−1^ (without steroid) doses of mRNA-1944. The percentage of participants achieving PRNT_50_ GMTs >100 and the GMTs for each dose group are provided. PRNT_50_ GMT >100 represents a level of CHIKV neutralizing antibodies previously associated with protection from both symptomatic CHIKV infection and subclinical seroconversion in humans^25,26^.

The neutralizing activity of CHKV-24 IgG against CHIKV was assessed in the serum of participants using the plaque reduction neutralization test (PRNT) after the administration of single doses of 0.1, 0.3 and 0.6 mg kg^−1^ mRNA and placebo at 12, 24 and 48 h. Dose-dependent neutralizing antibody titers were observed in all participants who were administered mRNA-1944 at all dose levels with no observable PRNT_50_ geometric mean titers (GMTs) in placebo recipients at the limit of detection (<10) by 12 h (Supplementary Fig. 2). After single doses of mRNA-1944 at the 0.3 and 0.6 mg kg^−1^ doses, 100% of participants had achieved PRNT_50_ GMTs >100, a level previously associated with protection from CHIKV infection in humans^25,26^, and persisted through 48 h (Fig. 3). Overall, these data indicated that functional CHKV-24 IgG antibodies were produced by mRNA-1944.

### Other exploratory parameters

Two participants on placebo had pre-existing anti-polyethylene glycol (PEG) mean titers of 223 at day 1 that were detectable through 24 weeks then were undetectable through 48 weeks. None of the participants treated with mRNA-1944 had detectable anti-PEG or anti-CHKV-24 IgG antibodies before or after administration of treatment at all doses. Transient increases in serum levels of C-reactive protein, complement, interleukin-6 (IL-6) and interferon gamma-induced protein 10 (IP-10) were observed at 12–24 h postdose that rapidly declined during 48 h among participants who received single doses of mRNA-1944 in the 0.6 mg kg^−1^ (with or without steroid) and 0.3 mg kg^−1^ 2-dose groups compared with participants who received placebo (Supplementary Fig. 3). Increases in the levels of these proteins were similar after the first and second doses at 0.3 mg kg^−1^ mRNA-1944 with no exacerbation of these elevations noted on administration of the second dose.

## Discussion

Given the continued global spread of CHIKV infection and its attendant morbidity, there is a need to develop vaccines and therapeutics. Currently, with no approved vaccines or therapeutics, CHIKV control measures rely on the use of personal protective measures and vector control^6^. While vaccines are highly effective in controlling vector-borne disease such as for yellow fever and Japanese encephalitis, given the rapid spread and high attack rates of CHIKV, therapeutics would also be beneficial^6^. As such, various vaccine and passive immunization strategies are under evaluation. This first-in-human phase 1 trial evaluated the safety and tolerability of escalating doses of mRNA-1944, encoding a CHIKV neutralizing antibody, administered by intravenous infusion in adults aged 18–50 years as a potentially adjunctive therapy to vaccination to help prevent and better control worldwide CHIKV infection. Single administration of mRNA-1944 at doses of 0.1, 0.3 and 0.6 mg kg^−1^ and a second dose at the intermediate 0.3 mg kg^−1^ level elicited dose-dependent increases in CHKV-24 IgG at levels predicted to provide protection against human infection and disease based on previous studies and also had an acceptable safety profile at these doses^13,25,26^. Thus, mRNA-1944 may offer a therapeutic option for the prevention and treatment of CHIKV infection, although additional longer-term studies in larger populations are needed.

Across doses, AEs in the study were mild to moderate in severity and transient in duration. Most treatment-related AEs reported were infusion-related reactions. The most commonly reported AEs were headache, nausea and myalgia in the mRNA-1944 group, and chills, fatigue and nausea in participants on placebo. There were few treatment-related AEs at the lower doses or in the placebo group, with the highest frequencies of AEs observed in the 0.6 mg kg^−1^ groups and in the 0.3 mg kg^−1^ combined for 2 doses group. Most AEs were grade 1 (35.7%) and 2 (21.4%) events across the mRNA-1944 groups with a single grade 3 (3.6%) AE of sinus tachycardia reported in 1 participant in the 0.6 mg kg^−1^ group, which resolved without intervention within 16 h postdose. There were no deaths or serious AEs or discontinuations due to AEs; AEs did not worsen with a second dose.

Although encapsulation of mRNA in LNPs ensures robust delivery to the cytosol, such particles can elicit immune-related and cellular toxicities^33,56^. As shown in animal models, LNPs can activate the complement system and also splenic B cells that produce anti-PEG antibodies; however, optimal mRNA and LNP formulation can mitigate these effects^33,56^. In our phase 1 study, detectable levels of ionizable lipid component were readily eliminated within a terminal mean half-life of 9.2 h; intervention-induced anti-PEG and anti-CHKV-24 IgG antibodies were not detected. Additionally, while systemic delivery of foreign RNA can activate an innate immune response, the mRNAs encoding CHKV-24 IgG were generally eliminated within 48 h postdose in the serum of participants. Postdose levels of C-reactive protein, complement and the acute phase reactants IL-6 and IP-10 were transient and declined within 48 h, which is consistent with the rapid elimination of both LNP and mRNA.

The use of mRNA technology for protein production may minimize the need to deliver high doses of antibody typically used for therapeutic antibodies and the exposure necessary for maximal effect^33,57^. Transient production of high local concentrations of mRNA-encoded proteins of interest can be obtained within hours in the absence of dose-limiting toxicity^33,57^. In this study, single administration of mRNA-1944 encoding the light and heavy chains of CHKV-24 IgG by intravenous infusion resulted in rapidly generated levels of neutralizing antibodies at all doses tested by 12 h that peaked within 48 h with a measured mean half-life of approximately 69 d. Additionally, the high levels of antibody achieved by 36–48 h exceeded the anticipated protective CHIKV neutralizing antibody level of 1 µg ml^−1^, shown previously to be associated with protection from both symptomatic CHIKV infection and subclinical seroconversion^25,26^. At the 0.3 and 0.6 mg kg^−1^ doses, antibody levels persisted for a least 16 weeks postdose and 100% of participants achieved a PRNT_50_ GMT >100, with a favorable safety profile. Administration of a second dose of mRNA-1944 at the intermediate 0.3 mg kg^−1^ level led to a 1.8-fold increase in the concentration of CHKV-24 IgG, with an extended terminal t_1/2_ of CHKV-24 IgG similar to the mean half-life observed across single doses, while maintaining an acceptable safety profile.

In this study, the two separate mRNAs encoding the heavy and light chains of CHKV-24 IgG produced functional neutralizing antibody and displayed consistent serum exposure ratios for the CHKV-24 IgG heavy-to-light chain mRNAs across the dose groups (C_max_ = 1.5–1.8 µg ml^−1^). Overall, these data provide the first demonstration in a clinical trial that antibody production by mRNA can safely achieve predicted therapeutically relevant serum concentrations, supporting the potential for this approach to have extended utility across various infectious diseases beyond CHIKV infection and in other therapeutic areas. Utilization of the mRNA platform to produce multiple therapeutic proteins simultaneously was also demonstrated in the phase 1 trial of a cytomegalovirus mRNA-1647 vaccine, consisting of five mRNAs encoding the subunits of the viral pentameric antigen complex and an additional mRNA encoding the viral glycoprotein B that resulted in high neutralizing titers^44^. While mRNA technology allows for simultaneous translation and complex protein formation from separate mRNAs, the expression of combinations of antibodies would require designing single-chain antibodies to prevent misassortment of heavy and light chains.

Although mRNA therapeutics are not yet commercially available, various mRNA vaccines have shown encouraging results in early-stage clinical studies^39–41,44^. More recently, larger-scale testing of mRNA vaccines in phase 3 clinical trials has demonstrated protective efficacy against symptomatic SARS-CoV-2 infection^42,43^. The success of these latter trials and data reported herein could augment the development of additional mRNA platform approaches for treating emerging and existing pathogens.

This study has several limitations. Since the study was designed as a dose-escalating phase 1 trial in healthy adults in the USA and participants were primarily white, additional studies are needed to fully assess the potential of this therapeutic approach in CHIKV-endemic regions and other populations. Although robust neutralizing activity was observed in serum, the duration of neutralizing antibody was assessed only up to 48 h per the protocol and longer-term studies are needed. Additionally, although the second dose of 0.3 mg kg^−1^ mRNA-1944 resulted in a 1.8-fold linear increase in serum CHKV-24 IgG that persisted well above predicted levels of CHKV neutralizing activity, assessment of neutralizing activity was not specified in the protocol. Additionally, this study describes a therapeutic monoclonal antibody approach for the treatment of CHIKV as a potentially adjunctive therapy to vaccination using an intravenous infusion route to rapidly generate high levels of antibody. A general limitation to this approach is that intravenous delivery in low- or middle-income countries could be challenging in settings with restricted resources. While antibody concentrations were generated that were expected to be sufficient for infection prophylaxis based on preclinical studies and an observed relationship between pre-existing CHIKV antibodies and a decreased risk of symptomatic CHIKV infection in humans^13,25,26^, it is unknown what levels of CHIKV monoclonal antibody would be required for therapeutic use to prevent infection.

In conclusion, this study demonstrated that administration of LNP mRNA-1944 encoding the heavy and light chains of CHKV-24 IgG produced high levels of functional neutralizing antibody and that CHKV-24 IgG persisted for several months at levels above the anticipated protective titers needed to prevent CHIKV infection, with an acceptable safety profile and predictable intra- and interindividual pharmacology. These results suggest that an mRNA antibody approach may have utility in the prevention and treatment of CHIKV infection. Furthermore, this study demonstrates the ability to produce functional proteins from separate mRNAs encoding different proteins and the feasibility of using a repeat dose regimen with this mRNA platform. Although the practicality of LNP-encapsulated mRNAs for passive immunization has been described in preclinical trials^36–38,54^, this is the first clinical trial, to our knowledge, that evaluates the safety and pharmacology of an mRNA encoding a monoclonal antibody as a potential prophylactic and therapeutic approach to treating disease. Future longer-term studies are needed to further evaluate the potential protective effect of this therapeutic approach in a wider population, including in those in CHIKV-endemic areas.

## Methods

### Study design and participants

This is a first-in-human phase 1, randomized, investigator-blinded (except for sentinel participants), placebo-controlled, dose escalation study to evaluate the safety, tolerability, PK and PD (CHKV-24 IgG) of mRNA-1944 in healthy adult participants aged 18–50 years (NCT03829384). Participants received intravenous single doses of mRNA-1944 or placebo at 0.1, 0.3, 0.6 mg kg^−1^ and 0.6 mg kg^−1^, or two weekly doses at 0.3 mg kg^−1^. The study was conducted at a single site in accordance with the International Council on Harmonization of Good Clinical Practice guidelines and the protocol was approved by regulatory (U. S. Food and Drug Administration) and institutional review boards (Western Institutional Review Board). All participants provided written informed consent.

Eligible participants were male and female adults, aged 18–50 years, weighing 50 to ≤90 kg with a maximum BMI ≤33 kg m^−2^ and considered by the investigator to be in good general health based on medical history, clinical laboratory assessments, electrocardiogram (ECG) results, vital sign measurements and physical examination findings. Excluded from the study were those having acute or chronic clinically important disease, elevated liver function tests and creatinine or decreased platelets and safety laboratory test results of grade 2 or higher. Also excluded were those who had anti-CHIKV IgG, had participated in an investigational study involving any investigational product within 60 d or 5 half-lives, received any live attenuated or inactive vaccines within 4 weeks before the study start or had plans to receive any vaccine during the study, or received a vaccine for CHIKV, dengue, yellow fever, tick-borne encephalitis or Japanese encephalitis virus at any time, or previously participated in an investigational study involving LNPs. Receipt of a seasonal influenza vaccine was permissible. Those with a known or suspected immune-mediated disease or immunosuppressive condition (including lymphoproliferative disorders) as determined by medical history and/or physical examination were also excluded. Pregnant or breastfeeding females and sexually active male and female participants or those unwilling to use adequate contraception for at least 36 weeks after the study drug infusion were also excluded. A full listing of inclusion and exclusion criteria is provided in the online protocol (Supplementary information).

### Treatment

Since mRNA-1944 was administered for the first time to humans, safety precautions such as sequential enrollment, premedication, dose escalation and continuous safety evaluations were overseen by an internal safety team (IST) and an unblinded independent safety monitoring committee (SMC). The investigator, study participants, site monitors and study site personnel were blinded to the study drug administered with the exceptions of the three unblinded sentinel participants at each dose level and unblinded pharmacy personnel, study monitor and clinical trial manager, as well as an unblinded team that provided safety data to the SMC.

All doses of mRNA-1944 and placebo were administered intravenously as a single 100 ml infusion in 2 syringes using a syringe infusion pump over 1–3 h at the study site (PPD Pharmacology Unit, Austin, Texas) by appropriately trained clinic staff. Participants were observed for 48 h afterwards as inpatients. On day 1, all participants in the dose level groups 0.1 and 0.3 mg kg^−1^ and the expansion group of the 0.6 mg kg^−1^ group were premedicated with the antihistamine loratadine (10 mg, oral) and the histamine-2 blocker ranitidine (150 mg, oral) approximately 90 min before the planned start of study drug infusion. Acetaminophen (650 mg, oral) was also given to the expansion participants in the 0.6 mg kg^−1^ group as premedication. Because 1 participant in the 0.6 mg kg^−1^ dose level cohort (after receiving the oral premedication regimen) developed a persistent grade 2 or higher infusion-related reaction (IRR), the other participants in the 0.6 mg kg^−1^ group were additionally premedicated with the steroid dexamethasone (10 mg, intravenous) to assess the impact of steroid on IRRs; loratadine (10 mg, oral) was replaced with diphenhydramine (50 mg, oral) and ranitidine with famotidine (20 mg, oral). The 0.3 mg kg^−1^ 2-dose group received diphenhydramine (50 mg, oral) and famotidine (20 mg, oral) as premedication at each day 1 and day 8 doses.

The mRNA-1944 drug product consisted of 2 mRNAs encoding the heavy and light chains of the previously described CHKV-24 monoclonal antibody^54^ in an LNP formulation intended for intravenous infusion. This mRNA contains the previously described LS mutations (M428L and N434S) in the constant (Fc) region of the antibody to extend its serum half-life^49–51,58^. The mRNA-1944 drug product formulation included four lipid excipients: a proprietary IAL; a proprietary high-purity PEG-2k-stearate monoester (modified PEG); and the commercially available lipids cholesterol and 1,2-distearoyl-sn-glycero-3-phosphocholine. The mRNA-1944 drug product was formulated with 20 mM of Tris buffer, 60 mM of NaCl, 8% sucrose, 1.3% ethanol and 1 mM of diethylenetriaminepentaacetic acid at pH 7.5; placebo was 0.9% sodium chloride. Additional details describing the overall planned treatment strategy are provided in the Supplementary protocol.

### Procedures

Seven dose groups of mRNA-1944 were planned to be investigated in a dose escalation manner (Supplemental protocol) including single-dose groups of 0.1, 0.3, 0.6 and 1.0 mg kg^−1^ and an optional 0.45 mg kg^−1^ dose group and a 2-dose 0.3 mg kg^−1^ group administered single doses of 0.3 mg kg^−1^ on days 1 and 8. A second single-dose 0.6 mg kg^−1^ group was added, which received steroid in the premedication regimen for both placebo and mRNA-1944 groups to enable a more robust characterization of the PK, PD and adverse reaction profile. A steroid (dexamethasone) was also added to the premedication regimen in the 1.0 mg kg^−1^ dose level group. Neither the optional 0.45 mg kg^−1^ dose group nor the planned 1.0 mg kg^−1^ group were enrolled in the study.

Eight participants were planned to be enrolled at each dose level group with six participants to receive mRNA-1944 and two to receive placebo. For each dose level group, a sentinel dosing strategy was employed. In each group, mRNA-1944 was administered to three participants (sentinel), one participant at a time with a staggered minimum 7-d interval between each sentinel participant before treating the remaining five participants (expansion) within each dose group. Each sentinel participant was followed up for 7 d after study drug infusion (first 48 h administered as an inpatient) with a review of safety results by the IST. After the confirmation of acceptable safety and tolerability by the IST for all sentinel participants, including the cumulative safety results, at day 7 enrollment was expanded and the remaining 5 participants within each dose level group were randomly assigned to receive mRNA-1944 (0.1, 0.3 and 0.6 mg kg^−1^) or placebo in a 3:2 ratio (active:placebo) for an overall ratio of 3:1. Randomization numbers were assigned in a sequential ascending manner and sealed in an envelope, according to a schedule developed by PPD Laboratories.

The SMC reviewed unblinded safety data of the entire dosed group through 7 d after the study drug infusion and cumulative safety data from all study participants before escalation to the next dose level. Dose escalation to higher-dose level groups could continue until a study pause rule was reached. Per protocol, optional dose level groups for intermediate dose levels could be added for de-escalation of the dose after review of cumulative data by the SMC and would be convened ad hoc in the event of a study pause. Furthermore, the sponsor could stop dose escalation once pharmacological goals were achieved. The start of the study was defined as the first visit for the first participant screened and study completion as the final date on which data for the primary end point were or expected to be collected if the final date was not the same. The end of study was defined as the last visit or last health status follow-up for the last participant discharged from the study. The approximate duration of participation for each participant will be 13 months, which includes a 28-d screening period.

Reasons for study pause included the occurrence of any serious AE, irrespective of assessed relatedness to study drug, or when two or more participants experienced a grade 3 or higher AE including laboratory abnormalities, or a grade 3 or higher IRR, or any clinical event in the opinion of the SMC that contraindicated further dosing of additional participants.

### Outcomes

The primary objective of this study was to evaluate the safety and tolerability of escalating doses of mRNA-1944 administered via intravenous infusion in healthy participants aged 18–50 years.

The secondary objectives included determination of the PK of mRNA encoding for CHKV-24 IgG (heavy and light chain mRNA) and IAL and the PD of mRNA-1944 as assessed by CHKV-24 IgG. Exploratory objectives of the study included evaluations of the formation of anti-PEG and anti-CHKV-24 IgG antibodies, the impact of several baseline characteristics on the PK/PD of mRNA-1944 encoding for CHKV-24 IgG and IAL and the in vitro serum neutralizing antibody titer against a clinically relevant strain of CHIKV, as well as complement and acute phase reactant parameters.

### Safety evaluation

Safety and tolerability were assessed by monitoring and recording AEs, including serious AEs, IRRs and AEs of special interest (AESIs), prior and concomitant medication, clinical laboratory test results (hematology, coagulation, serum chemistry including liver enzymes, urinalysis), vital sign measurements (systolic and diastolic blood pressure, heart rate, respiratory rate, body temperature), ECG results (and cardiac enzymes when obtained per protocol) and physical examination findings.

An AE was defined as any untoward medical occurrence in a participant administered a study drug regardless of a causal relationship with receipt of the study drug. Treatment-emergent AEs were those not present before study drug exposure or any event already present that worsened in intensity or frequency after exposure. A treatment-related AE was any event for which there was a reasonable possibility of its occurrence being causally associated with study treatment as determined by the investigator. An IRR was any AE considered to be related to the infusion of the study drug, including pain, tenderness, erythema/redness and induration/swelling; they were assessed according to the CTCAE v.5 toxicity grading scale, recorded as AEs and followed until resolution^55^. Suspected allergic (hypersensitivity) reactions and anaphylaxis were assessed according to the clinical diagnostic criteria outlined by the National Institute of Allergy and Infectious Diseases and CTCAE v.5 (refs. ^55,59^). AEs leading to study withdrawal or dose modification were also evaluated by the investigator. An AE was considered ‘serious’ if, in the view of either the investigator or study sponsor, it resulted in death, was life-threatening, requiring inpatient hospitalization or prolongation of existing hospitalization, persistent or substantial disability/incapacity, a congenital anomaly/birth defect or deemed a medically important event. AESIs related to the administration of immunostimulatory agents included hypersensitivity (signs and symptoms of generalized rash, generalized pruritus, generalized erythema, cough, dyspnea, respiratory distress, acute bronchospasm, hypotension and chest discomfort), anaphylactic reaction, acute allergic reaction, angioedema and allergic urticaria. AESIs also included gastrointestinal symptoms (nausea, diarrhea, abdominal pain, vomiting) and/or grade 2 or higher liver function test elevation.

AEs were assessed and recorded from the time of participant informed consent through 28 d after study drug infusion (after the second infusion in the 0.3 mg kg^−1^ 2-dose group) and were followed until resolved, stabilized or judged by the investigator to be not clinically important AEs were captured through the study follow-up period of 12 months. All medically attended AEs were recorded for 3 months after study drug infusion and all serious AEs and AESIs were recorded through week 52. Any new serious AE assessed as related to study drug infusion was reported to the sponsor, regardless of when it occurred, throughout the duration of the study.

The severity of AEs and laboratory abnormalities were classified by the investigator according to the toxicity grading scale of mild (grade 1), moderate (grade 2), severe (grade 3) and potentially life-threatening (grade 4) using the CTCAE v.5 grading scale and were used to determine if a pause criterion was met^55^. Vital signs were graded using the toxicity grading scale for healthy adult and adolescent volunteers enrolled in preventive vaccine clinical trials tables for clinical abnormalities^60^. The investigator determined whether there was a reasonable possibility that an event was caused by the study drug for all AEs, serious AEs and AESIs.

### PK, PD and other analyses

Details of the specific assays supporting these analyses are provided in the supplementary material.

### PK analyses

PK data for mRNA encoding for the CHKV-24 IgG and IAL were assessed from day 0 (predose) through 28 d for participants in the safety population who had evaluable mRNA encoding for CHKV-24 IgG. To determine the serum concentrations of mRNA encoding for CHKV-24 IgG and plasma concentrations of IAL, blood samples were collected for the 0.1, 0.3 and 0.3 mg kg^−1^ 2-dose and 0.6 mg kg^−1^ with steroid dose groups, and the sentinel participant in the 0.6 mg kg mg^−1^ group without steroid, at 60 min prestudy drug infusion, at mid-infusion (0.5 h), at the end of infusion (1 h + 5 min) and at 2, 4, 6, 8, 12, 18, 24 and 36 h and on days 7, 14, 21 and 28 postinfusion. For expansion participants in the 0.6 mg kg^−1^ without steroid dose group who underwent a 3-h infusion, blood samples were collected within 60 min predose, mid-infusion 1.5 h, end of infusion (3 h + 5 min), at 4, 6, 8, 12, 18, 24, 36 and 48 h and on days 7, 14, 21 and 28 postdose. Where possible, the area under the concentration versus time curve (AUC) from time 0 to the last measurable concentration (AUC_0-last_), AUC from time 0 extrapolated to infinity (AUC_0–inf_), C_max_, time to maximum observed serum concentration (t_max_), t_1/2_, apparent clearance and volume of distribution at steady state (V_ss_) were calculated as end points for mRNA encoding CHKV-24 IgG and for IAL, respectively, using the actual sampling times relative to the start of infusion rather than the scheduled sampling times.

### PD analyses

PD data for serum CHKV-24 IgG were assessed from day 0 (predose) through 366 d for 28 participants on active treatment in the safety population who had evaluable mRNA encoding for CHKV-24 IgG. Blood samples for the determination of serum concentrations of CHKV-24 IgG were collected for the 0.1, 0.3, 0.3 mg kg^−1^ 2-dose and 0.6 mg kg^−1^ with steroid dose level groups and for the sentinel participant in the 0.6 mg kg^−1^ group without steroid within 60 min before preinfusion, at mid-infusion (0.5 h), at end of infusion (1 h + 5 min) and at 2, 4, 6, 8, 12, 18, 24, 36 and 48 h, on days 7, 14, 21 and 28 and weeks 8, 12, 24, 36, 48 and 52 postinfusion. For the 3 expansion participants in the 0.6 mg kg^−1^ dose group, blood samples were collected within 60 min before study drug infusion, at mid-infusion (1.5 h), at end of infusion (3 h + 5 min), at 4, 6, 8, 12, 18, 24, 36 and 48 h, on days 7, 14, 21 and 28 and weeks 8, 12, 24, 36, 48 and 52 postdose. Baseline-corrected serum PD parameters of E_max_, time to maximum observed effect (TE_max_), t_1/2_, area under the effect curve (AUEC) from time 0 to the last measurable concentration (AUEC_0-last_) and area under the effect curve from time 0 extrapolated to infinity (AUEC_0–inf_) were calculated, where possible, for CHKV-24 IgG concentration using the actual sampling times relative to the start of infusion.

### Other analyses

Blood samples for the determination of in vitro serum neutralizing antibody titer against a clinically relevant strain of CHIKV were collected within 60 min before study drug infusion and at 12, 24 and 48 h postinfusion. For the 2-dose 0.3 mg kg^−1^ group, blood samples were collected within 60 min before study drug infusion and at 12, 24 and 48 h postdose on day 1 for the first dose and day 8 for the second dose. To determine the anti-PEG antibodies, blood samples were collected within 60 min before study drug infusion, on days 7, 14, 21 and 28 and weeks 8, 12, 24, 36, 48 and 52 postdose. Blood samples to determine the anti-CHKV-24 IgG antibodies were collected within 60 min before study drug infusion, on days 7, 14, 21 and 28 and weeks 8, 12, 24, 36, 48 and 52 postdose. Time points were relative to the start of study drug infusion.

### Statistical analysis

Per protocol, it was planned to enroll approximately 56 participants in the study, with 8 in each dose level group. No formal power calculations or hypotheses testing were performed. The planned sample size was regarded as appropriate to meet the objectives of this first-in-human study. The protocol-planned optional 0.45 mg kg^−1^ dose group was not enrolled, nor the planned 1.0 mg kg^−1^ group; thus, 40 participants were planned for the dose level groups included (0.1, 0.3, 0.6 mg kg^−1^ with and without steroid and 0.3 mg kg^−1^ 2-dose).

Safety and exploratory analyses were performed in the safety population, which comprised all enrolled participants who received any dose infusion of mRNA-1944 or placebo. The PK analysis dataset included participants in the safety population who had evaluable mRNA encoding for CHKV-24 IgG and IAL concentrations and no major protocol deviations. The PD analysis dataset included participants in the safety population who had evaluable CHKV-24 IgG concentrations and no major protocol deviations impacting the PD assessments. No protocol deviations occurred in the study.

Data are presented for participants on mRNA-1944 in each treatment group and those on placebo pooled across the different treatment groups. Baseline characteristics and demographics were summarized by descriptive statistics and safety and immunogenicity by summary statistics. Descriptive statistics and changes from baseline in continuous clinical laboratory results were summarized. Serum mRNA encoding for the CHKV-24 IgG and plasma IAL concentrations are listed and summarized descriptively by dose level (*n*, arithmetic mean, s.d., coefficient of variation (CV), median, geometric mean, geometric CV, minimum and maximum).

Noncompartmental PK parameters including AUC and half-life were derived using Phoenix WinNonlin (Certara) v.6.4 or higher or SAS v.9.3 or higher (SAS Institute) and were summarized by dose level using descriptive statistics (*n*, arithmetic mean, s.d., CV, median, geometric mean, geometric CV, minimum and maximum). For T_max_, only *n*, median, minimum and maximum are presented. Concentration values that were below the limit of quantitation were imputed as missing for the derivation mean and s.e.m. descriptive statistics for concentrations at each time point and for the calculation of individual noncompartmental analysis parameters. Concentration versus time profiles for each participant and the mean concentration versus scheduled time profiles are presented graphically. Additionally, concentrations and PK parameters were summarized by dose level using descriptive statistics for participants with a negative antidrug antibody response. The statistical relationship between dose and PK parameters was assessed overall and for participants with a negative antidrug antibody response. Periodic PK and PD analyses were performed after completion of all participants in all dose level groups. All data used for the interim analyses were unblinded to participant-level treatment assignments; related parameter values used for the interim analyses were derived from Phoenix WinNonlin v.6.4 or higher based on nominal dosage and times.

The data used for the interim safety analyses included participant disposition, demographic and baseline characteristics, study drug administration (including infusion rate changes), laboratory test results, AEs (both infusion- and non-infusion-related), previous and concomitant medication, ECGs (and cardiac enzymes when obtained per protocol) and vital signs. The interim results presented in this article are from a data cutoff date of 15 October 2020 and the data are summarized for at least eight weeks postdose per participant. AEs, PK and PD will be followed for 12 months in participants and a final analysis will be performed at end of study after database lock. The trial was anticipated to complete at the end of May 2021; however, due to COVID-19, the trial completion date was 7 June 2021.

### Reporting Summary

Further information on research design is available in the Nature Research Reporting Summary linked to this article.

## Online content

Any methods, additional references, Nature Research reporting summaries, source data, extended data, supplementary information, acknowledgements, peer review information; details of author contributions and competing interests; and statements of data and code availability are available at 10.1038/s41591-021-01573-6.

## Supplementary information

Supplementary Information Supplementary Assay Methods, Tables 1–3, Figs. 1–3 and clinical trial protocol

Reporting Summary

## Data Availability

Moderna is committed to sharing access to patient-level data and supporting clinical documents from eligible studies with external researchers who provide methodologically sound scientific proposals upon request once the trial is complete. Such requests can be made to Moderna. A redacted protocol is available online as supplementary material to this article.
