# Author Correction: A phase 1 trial of lipid-encapsulated mRNA encoding a monoclonal antibody with neutralizing activity against Chikungunya virus

**DOI:** 10.1038/s41591-022-01817-z

**Published:** 2022-04-21

**Authors:** Allison August, Husain Z. Attarwala, Sunny Himansu, Shiva Kalidindi, Sophia Lu, Rolando Pajon, Shu Han, Jean-Michel Lecerf, Joanne E. Tomassini, Marjie Hard, Leon M. Ptaszek, James E. Crowe, Tal Zaks

**Affiliations:** 1 Moderna, Inc., Cambridge, MA USA; 2 Massachusetts General Hospital, Boston, MA USA; 3 Vanderbilt University Medical Center, Nashville, TN USA

**Keywords:** Infectious diseases, Viral infection, Preventive medicine

Correction to: *Nature Medicine* 10.1038/s41591-021-01573-6, published online 9 December 2021.

In the version of this article initially published, there were errors in the inset graph to Fig. 2a, where the inset had the graph lines and symbols for the 0.3 mg kg^–1^ and the 0.6 mg kg^–1^ switched, and the wrong data were shown for the 0.6 mg kg^–1^ in the inset. The labels and plots have been corrected in the HTML and PDF versions of the article. The original and corrected Fig 2a graphs are shown below.

**Fig. 2:**
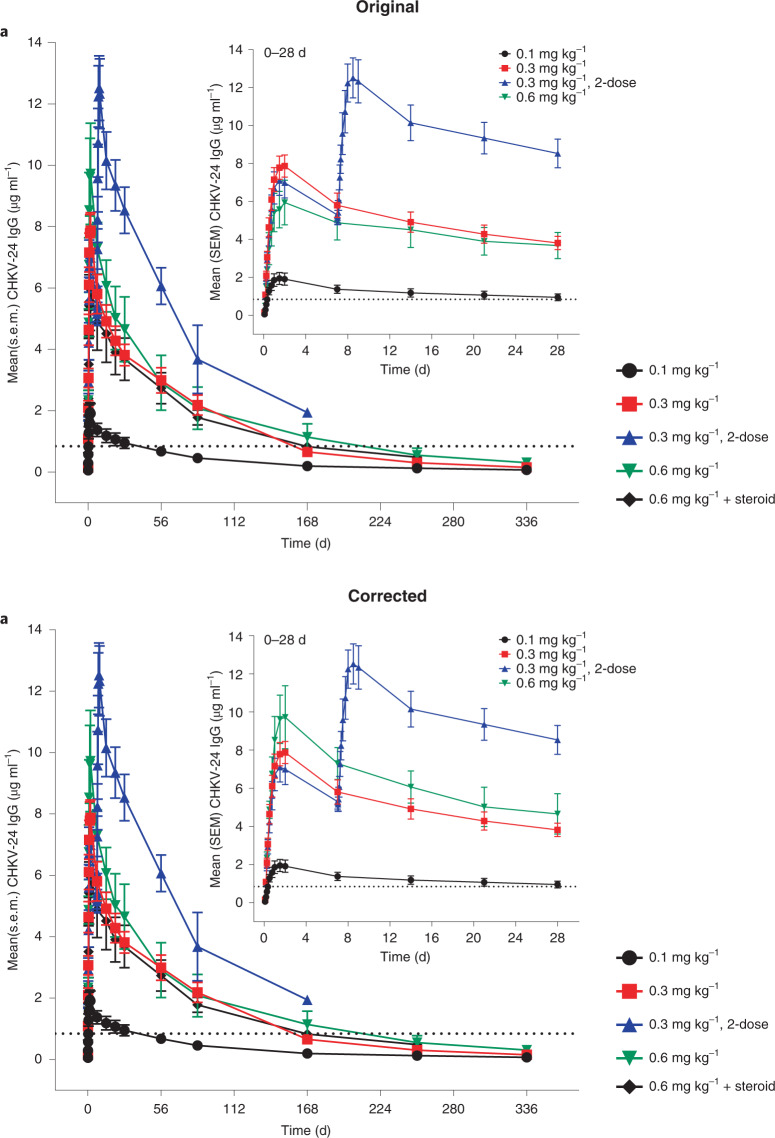
Original and corrected.

