# Supplementary material for: A phase 1 trial of lipid-encapsulated mRNA encoding a monoclonal antibody with neutralizing activity against Chikungunya virus
Source: Nat Med. 2021 Dec 9;27(12):2224–33. doi: 10.1038/s41591-021-01573-6 (PMC8674127; doi:10.1038/s41591-021-01573-6)
Supplement: Supplementary file 1 — Supplementary Assay Methods, Tables 1–3, Figs. 1–3 and clinical trial protocol [file 41591_2021_1573_MOESM1_ESM.pdf]

nature portfolio

https://doi.org/10.1038/s41591-021-01573-6

Supplementary information

A phase 1 trial of lipid-encapsulated mRNA encoding a monoclonal antibody with neutralizing activity against Chikungunya virus

In the format provided by the authors and unedited

SUPPLEMENTAL DATA

Assay methods

CHKV24 IgG mRNA

For the quantitation of CHKV24 IgG heavy and light chain mRNA in human serum, samples (standard calibrators, QCs and study samples) were diluted in lysis mixture prior to being mixed with working beads, capture probes, blocking probes and label extenders in a 96-well dilution plate. Following an overnight incubation and washing, pre-amplifier solution was added to the plate, incubated for 1 h then washed, and the amplifier solution was added to the plate. After a 1 h incubation and washing, Label probe solution was added to the plate, incubated for 1h and washed, after which the streptavidin-PE (SAPE) solution was added and incubated for 30 min. After washing, SAPE wash buffer was added, and the plate was shaken for approximatively 3 min before the beads were read in a BioPlex instrument. The assay sensitivity for the heavy chain and light chain mRNAs was 100 pg/mL and 55.6 pg/mL in neat serum, respectively.

CHKV24 IgG

CHKV24 IgG in human serum was quantitated using an assay that captured free therapeutic antibody (CHKV24 IgG) from patient serum on MDS MULTI-ARRAY® 96-well High Bind plates coated with ChikV181/25 viral lysate. Bound CHKV24 IgG was then detected by rabbit polyclonal anti-CHKV24 antibody (GensCript, Cat# SC1320A customized antibody, Lot A318060195, diluted to 249 ng/mL), followed by MSD Sulfo-tag labeled goat anti-rabbit antibody (MSD, Cat #R32AB-5, diluted to 500 ng/mL). After addition of MSD® 2X Read Buffer T, plates were read in a MSD Sector Imager. The electrochemiluminescence (ECL) intensity was proportional to the amount of CHKV24 IgG that bound to the plate. The sensitivity of the assay was 37.5 ng/mL in neat serum.

Chikungunya Virus Plaque Reduction Neutralization Test

Plaque reduction neutralization test (PRNT) assays were performed in the Galveston National Laboratory at the University of Texas Medical Branch (UTMB). Procedures involving live virus were conducted under Biosafety Level 3 conditions, and all laboratory personnel were blinded to sample identification. Test samples for the PRNT assay were removed from frozen storage (-80°C), thawed under ambient conditions, then heat-inactivated in a 56°C water bath for 60 minutes. For control purposes, naïve pooled human serum was processed in parallel.

Chikungunya virus 37997 (CHIKV) test inoculum was prepared fresh from frozen stock in Minimum Essential Medium (MEM, Gibco) to a final concentration of 1,000 PFU/mL. CHIKV strain 37997 was a clinically relevant strain originally isolated from the mosquito *Ae. furcifer* in Kadougou, Senegal, in 1983 (GenBank accession no. AY726732) and was recently characterized in Ae. aegypti mosquitoes and found to have consistently high infection and dissemination rates (Vanlandingham et al., 2005). Virus stock was initially generated via electroporation in low-passage Vero 76 cells (ATCC® CCL-81TM) using a genome-containing plasmid (generously provided by Dr. Scott Weaver at UTMB). Next generation sequencing of the stock confirmed 100% consensus sequence-level match (GenBank accession AY726732.1). Test and control samples were serially diluted 2-fold in MEM beginning at a 1:5 dilution. An equivalent volume of CHIKV inoculum was added and incubated for 60 minutes at 37°C. Samples were added to confluent Vero 76 cell monolayers in 12-well format (0.1 mL per 3.8 cm^2 well), and incubated at 37°C/5% CO_2 for 60 minutes, after which 1.25% carboxymethylcellulose overlay medium (1 mL) was added to each well. Following an additional 48 h at 37°C/5% CO_2, 10% neutral buffered formalin (1 mL) was added to each well and incubated under ambient conditions for 60 minutes. Overlay/formalin was decanted and wells were stained with 1 mL of 0.25% crystal violet solution for 10 minutes, then thoroughly washed with tap water. Plaques from all wells were enumerated by direct visualization. Using average plaque count data, the $PRNT_{50}$, defined as the reciprocal of the highest dilution resulting in 50% plaque reduction compared to virus-only control, was determined for each sample. The limit of detection was defined as 1 plaque per well. $PRNT_{50}$ GMT >100 represents a level of neutralizing antibody shown previously to be associated with protection from both symptomatic CHIKV infection and subclinical seroconversion in humans.[26,27]

Anti-CHKV24 IgG antibodies

Anti-CHKV24 IgG antibodies in human serum were detected using a bridging assay. CHKV24 mastermix [CHKV24-biotin + CHKV24-SULFO-TAG + assay buffer] was added to a storage plate, followed by the addition of required samples to appropriate wells and incubated at ambient temperature for 1 hour. Anti-CHKV24 rabbit polyclonal antibody was used as a positive control. The samples were transferred to duplicate wells of a pre-blocked and washed MSD® MULTI-ARRAY® Gold 96-well SA-coated plates. The plate was incubated at ambient temperature for 1 hour and washed. Finally, 2X Read Buffer T was added to the wells and the plate was read in a MSD Sector Imager. The assay sensitivity was

19.5 ng/mL. For the confirmatory tier, samples were incubated with CHKV24 IgG to specifically compete the signal.

Anti-PEG antibodies

Anti-PEG antibodies in human serum were detected by capture of human anti-PEG antibodies on mPEG-biotin streptavidin-coated plates. mPEG-biotin was added at 1 µg/mL to wells of streptavidin-coated plates and incubated at ambient temperature for 1 hour. After decantation, the plates were blocked with Blocker Casein in PBS at ambient temperature for 1-4 hours. After washing the plates, serum samples (study samples, positive and negative controls) were added and the plates were incubated at ambient temperature for 1 hour. After washing, the bound anti-PEG antibodies were detected by addition of goat anti-human IgG,M (H+L) polyclonal antibodies conjugated with HRP (Jackson lmmunoResearch, Cat#109-036-127, Lot 113285, diluted 1/20000) at ambient temperature for 75 ± 15 min. Plates were washed, and chromogenic substrate was added to the wells at ambient temperature for 20 ± 2 min, then read on a plate reader. The colorimetric signal was proportional to the amount of anti-PEG antibodies present in patient samples. The assay sensitivity was 24.4 ng/mL. For the confirmatory tier, samples were incubated with mRNA-1944 drug product that contained PEG to specifically compete the signal.

Complement factors

For the assessment of complement factors C5b-9 blood samples were collected pre-dose (within 60 minutes before study drug infusion), and at 90 (±5) minutes (from the start of infusion); at 2, 6, 12, and 24 (±15 minutes) h post-dose (completion of the infusion); and at the time of discharge (within 60 minutes) from the inpatient unit. If the participant experienced an infusion reaction, a blood sample for analysis of complement factors was obtained within 1 h of the start of the reaction. Blood samples for the assessment of C-reactive protein (CRP) were collected pre-dose (within 60 min before study drug infusion) and 6- and 24-h post-dose (completion of infusion), and at the time of discharge (within 60 min) from the inpatient unit. Blood samples for IL 6 and IP-10 were collected pre-dose (within 60 minutes before study drug infusion), and at 90 minutes (±5) (from the start of infusion); at 2, 6, 12, and 24 (±15 min) h post-dose (completion of the infusion); and at the time of discharge (within 60 min) from the inpatient unit. If the participant experienced an infusion reaction, a blood sample for analysis of CRP and cytokine factors would be obtained within 1 h of the start of the reaction.

Table S1. Treatment-related AEs by grade

Treatment-related AEs by SOC, preferred term, CTCAE n (%)	mRNA-1944 0.1 mg/kg[a] N=6	mRNA-1944 0.3 mg/kg[a] N=6	mRNA-1944 0.6 mg/kg[b] N=4	mRNA-1944 0.6 mg/kg +steroid N=6	mRNA-1944 0.3 mg/kg 2 dose[d,e] N=6	mRNA-1944 All N=28	All Placebo N=10
Headache		1 (16.7)	3 (75.0)	4 (66.7)	1 (16.7)	9 (32.1)	
Grade 1		1 (16.7)	2 (50.0)	3 (50.0)	1 (16.7)	7 (25.0)	
Grade 2			1 (25.0)	1 (16.7)		2 (7.1)	
Nausea			2 (50.0)	2 (33.3)	3 (50.0)	7 (25.0)	1 (10.0)
Grade 1				2 (33.3)	2 (33.3)	4 (14.3)	1 (10.0)
Grade 2			2 (50.0)		1 (16.7)	3 (10.7)	
Myalgia				4 (66.7)	1 (16.7)	5 (17.9)	
Grade 1				4 (66.7)	1 (16.7)	5 (17.9)	
Chills			2 (50.0)	1 (16.7)	1 (16.7)	4 (14.3)	1 (10.0)
Grade 1			2 (50.0)	1 (16.7)	1 (16.7)	4 (14.3)	1 (10.0)
Dizziness			2 (50.0)	1 (16.7)	1 (16.7)	4 (14.3)	
Grade 1			2 (50.0)	1 (16.7)	1 (16.7)	4 (14.3)	
Vomiting			2 (50.0)		2 (33.3)	4 (14.3)	
Grade 2			2 (50.0)		2 (33.3)	4 (14.3)	
Increased heart rate				3 (50.0)		3 (10.7)	
Grade 1				3 (50.0)		3 (10.7)	
Pyrexia			2 (50.0)			2 (7.1)	
Grade 1			1 (25.0)			1 (3.6)	
Grade 2			1 (25.0)			1 (3.6	
Back pain				1 (16.7)	1 (16.7)	2 (7.1)	
Grade 1				1 (16.7)	1 (16.7)	2 (7.1)	
Musculoskeletal stiffness			2 (50.0)	1 (16.7)		2 (7.1)	
Grade 1			2 (50.0)	1 (16.7)		2 (7.1)	
Increased white blood cell count			1 (25.0)	1 (16.7)		2 (7.1)	
Grade 1				1 (16.7)		1 (3.6)	
Grade 3			1 (25.0)			1 (3.6)	
Flushing				1 (16.7)	2 (33.3)	3 (10.7)	
Grade 1				1 (16.7)	2 (33.3)	3 (10.7)	
Sinus tachycardia			2 (50.0)			2 (7.1)	
Grade 1			1 (25.0)			1 (3.6)	
Grade 3			1 (25.0)			1 (3.6)	
Decreased appetite					2 (33.3)	2 (7.1)	
Grade 1					2 (33.3)	2 (7.1)	
Hyperhidrosis				1 (16.7)	1 (16.7)	2 (7.1)	
Grade 1				1 (16.7)	1 (16.7)	2 (7.1)	
Dizziness postural					1 (16.7)	1 (3.6)	
Grade 1					1 (16.7)	1 (3.6)	
Dysgeusia					1 (16.7)	1 (3.6)	
Grade 1					1 (16.7)	1 (3.6)	
Lethargy				1 (16.7)		1 (3.6)	
Grade 1				1 (16.7)		1 (3.6)	
Presyncope				1 (16.7)		1 (3.6)	
Grade 2				1 (16.7)		1 (3.6)	

Fatigue				1 (16.7)	1 (3.6)	1 (10.0)
Grade 1				1 (16.7)	1 (3.6)	1 (10.0)
Feeling hot	1 (16.7)				1 (3.6)	
Grade 1	1 (16.7)				1 (3.6)	
Feeling of body temperature change				1 (16.7)	1 (3.6)	
Grade 1				1 (16.7)	1 (3.6)	
Infusion site erythema				1 (16.7)	1 (3.6)	
Grade 1				1 (16.7)	1 (3.6)	
Abdominal discomfort			1 (16.7)		1 (3.6)	
Grade 1			1 (16.7)		1 (3.6)	
Abdominal pain				1 (16.7)	1 (3.6)	
Grade 1				1 (16.7)	1 (3.6)	
Flatulence		1 (16.7)			1 (3.6)	
Grade 1		1 (16.7)			1 (3.6)	
Retching				1 (16.7)	1 (3.6)	
Grade 2				1 (16.7)	1 (3.6)	
Blood pressure increased				1 (16.7)	1 (3.6)	
Grade 1				1 (16.7)	1 (3.6)	
Electrocardiogram T wave inversion		1 (25.0)			1 (3.6)	
Grade 2		1 (25.0)			1 (3.6)	
Hypotension		1 (25.0)			1 (3.6)	
Grade 1		1 (25.0)			1 (3.6)	
Rash Maculo-papular			1 (16.7)		1 (3.6)	
Grade 1			1 (16.7)		1 (3.6)	
Vertigo		1 (16.7)			1 (3.6)	
Grade 1		1 (16.7)			1 (3.6)	
Infusion-related reaction		1 (25.0)			1 (3.6)	
Grade 1		1 (25.0)			1 (3.6)	
Anxiety				1 (16.7)	1 (3.6)	
Grade 1				1 (16.7)	1 (3.6)	
Menstruation irregular		1 (16.7)			1 (3.6)	
Grade 1		1 (16.7)			1 (3.6)	

AE=adverse event, SOC=system organ class. AEs coded using MedDRA Version 23.0 (Grade 1 = Mild; Grade 2 = Moderate; Grade 3 = Severe; Grade 4 = Life Threatening). Participants received loratadine and ranitidine[a] or loratadine, ranitidine (sentinel, expansion) and acetaminophen (expansion)[b] or steroid (dexamethasone), and diphenhydramine and famotidine[c] or diphenhydramine and famotidine[d] 90 minutes prior to infusion. [e]Participants were administered two 0.3 mg/kg doses administered on Days 1 and 8. AEs listed in decreasing frequency >3% for overall active treatment population.

Table S2. Treatment-related Adverse Events by SOC Preferred Term, CTCAE in 2 dose, 0.3 mg/kg mRNA-1944 Group

Treatment-related AEs by SOC, preferred term, CTCAE n (%)	0.3 mg/kg mRNA-1944 N=6						Placebo N=2	
	Dose 1		Dose 2		Total		Dose 1	Dose 2
	Grade 1	Grade 2	Grade 1	Grade 2	Grade 1	Grade 2	Grade 1	
Headache	1 (16.7)				1 (16.7)			
Dizziness	1 (16.7)				1 (16.7)			
Dizziness postural			1 (16.7)		1 (16.7)			
Dysgeusia	1 (16.7)				1 (16.7)			
Chills	1 (16.7)				1 (16.7)		1 (50.0)	
Fatigue	1 (16.7)				1 (16.7)			
Feeling of body temperature change	1 (16.7)		1 (16.7)		2 (33.0)			
Infusion site erythema			1 (16.7)		1 (16.7)			
Nausea		1 (16.7)	3 (50.0)		3 (50.0)	1 (16.7)	1 (50.0)	
Vomiting		2 (33.0)				2 (33.0)		
Abdominal pain	1 (16.7)				1 (16.7)			
Retching				1 (16.7)		1 (16.7)		
Myalgia	1 (16.7)		1 (16.7)		2 (33.0)			
Back pain	1 (16.7)		1 (16.7)		2 (33.0)			
BP increased	1 (16.7)				1 (16.7)			
Flushing			2 (33.0)		2 (33.0)			
Hyperhidrosis			1 (16.7)		1 (16.7)			
Decreased Appetite	1 (16.7)		1 (16.7)		2 (33.0)			
Anxiety			1 (16.7)		1 (16.7)			

AE=adverse event, SOC=system organ class. AEs coded using MedDRA Version 23.0 (Grade 1 = Mild; Grade 2 = Moderate; Grade 3 = Severe; Grade 4 = Life Threatening). Participants received diphenhydramine and famotidine 90 minutes prior to infusion and were administered two 0.3 mg/kg doses on Days 1 and 8.

Table S3. Additional pharmacokinetic and pharmacodynamic parameters

	mRNA-1944	mRNA-1944	mRNA-1944	mRNA-1944	mRNA-1944	mRNA-1944
	0.1 mg/kg[a]	0.3 mg/kg[a]	0.6 mg/kg[b]	0.6 mg/kg +steroid[c]	0.3 mg/kg 2 dose[d,e] 1st dose	0.3 mg/kg 2 dose[d,e] 2nd dose
**Parameter**	N=6	N=6	N=4	N=6	N=6	N=6
**CHKV24 IgG**						
E_{max}, µg/mL						
Mean	2.0	7.9	10.2	6.1	7.2	12.9
SD	0.8	1.4	3.0	2.9	1.9	2.8
CV%	40.4	18.2	29.6	47.0	26.9	21.4
Median	1.9	7.6	9.7	6.1	7.1	13.0
Minimum	1.1	6.3	7.0	2.3	4.0	8.2
Maximum	3.1	10.0	14.2	10.5	9.7	16.7
TE_{max}, h						
Median	36.0	48.0	48.0	48.0	36.0	42.0
Minimum	24.0	36.0	36.0	24.0	24.0	24.0
Maximum	48.0	48.1	48.0	48.0	48.0	48.0
AUECO-last, µg*h/mL						
Mean	2700	11200	12900	9650	965	11700
SD	1590	2720	7290	3470	273	4850
CV%	58.9	24.2	56.7	35.9	28.3	41.6
Median	2330	10500	11900	9400	941	9890
Minimum	1040	7650	5020	4260	535	7640
Maximum	5490	15400	22600	13500	1360	21200
AUECO-168, µg*h/mL						
Mean	260	1070	1310	846	965	1830
SD	104	234	262	408	273	401
CV%	40.0	21.9	20.0	48.2	28.3	21.9
Median	250	992	1360	844	941	1830
Minimum	151	841	957	349	535	1170
Maximum	408	1390	1570	1510	1360	2390
AUEC0-inf, µg*h/mL						
Mean	2820	11600	13400	11200	NA	23400
SD	1660	2850	7790	3360	NA	6300
CV%	58.6	24.6	58.1	30.0	NA	26.9
Median	2390	10900	12200	11500	NA	24700
Minimum	1140	7800	5250	5680	NA	12400
Maximum	5760	16100	24000	14600	NA	31500
$t_{1/2}$, h						
Mean	1470	1930	1570	1720	NA	1520
SD	508	297	764	413	NA	318
CV%	34.6	15.4	48.8	24.1	NA	20.9
Median	1590	1990	1800	1780	NA	1450
Minimum	614	1470	458	1030	NA	1280
Maximum	1980	2320	2210	2200	NA	2140
**mRNA Light chain**						
C_{max}, µg/mL						
Mean	0.1	0.3	1.5	0.9	0.5	0.6
SD	0.1	0.3	0.7	0.4	0.2	0.2
CV%	70.7	89.9	44.4	48.5	45.6	30.3
Median	0.1	0.2	1.7	0.9	0.5	0.6
Minimum	0.1	0.1	0.6	0.3	0.2	0.3
Maximum	0.3	0.7	2.2	1.6	0.9	0.8
tmax, h						
Median	12.1	3.5	2.0	2.0	4.0	2.0
Minimum	4.0	1.0	1.0	2.0	1.0	1.0
Maximum	48.0	18.0	8.0	4.0	6.0	6.0
AUCO-last, µg*h/mL						
Mean	11.2	26.7	89.3	60.2	29.1	30.9
SD	8.8	19.6	16.7	20.3	14.3	7.6
CV%	78.8	73.3	18.7	33.8	49.1	24.5
Median	8.4	25.8	83.0	62.3	27.0	27.6
Minimum	3.4	4.7	77.5	25.6	14.1	22.4
Maximum	27.2	54.2	114	84.6	50.3	41.4
AUCO-168, µg*h/mL						
Mean	8.0	17.7	70.4	49.4	32.2	26.9
SD	6.1	13.8	19.4	17.7	13.7	8.2
CV%	75.6	77.8	27.6	35.8	42.6	30.3
Median	6.7	13.5	67.7	52.0	31.6	24.1
Minimum	3.1	3.5	49.8	17.3	16.1	18.3
Maximum	20.0	35.7	96.7	69.7	50.3	37.3

AUC0-inf, µg*h/mL						
Mean	11.6	27.2	89.9	60.4	37.9	NA
SD	8.9	20.0	16.6	20.4	16.6	NA
CV%	77.4	73.6	18.4	33.8	43.8	NA
Median	8.8	26.6	84.0	62.4	37.0	NA
Minimum	3.4	4.7	77.5	25.8	19.3	NA
Maximum	27.6	55.1	114	84.9	60.8	NA
$t_{1/2,}$ h						
Mean	87.4	88.8	82.5	68.6	62.6	61.2
SD	46.0	28.0	29.6	21.2	5.92	14.0
CV%	52.6	31.5	35.9	30.9	9.4	22.9
Median	82.4	88.4	78.2	71.8	63.7	63.9
Minimum	39.0	49.6	51.8	35.5	56.9	44.3
Maximum	155	127	122	91.1	71.2	78.3
CL, mL/h/kg						
Mean	4.6	7.4	2.3	3.8	3.1	3.4
SD	3.2	7.3	0.4	2.0	1.4	0.8
CV%	69.6	99.4	16.0	51.6	45.9	22.5
Median	3.9	4.8	2.4	3.2	2.7	3.6
Minimum	1.2	1.8	1.8	2.4	1.6	2.4
Maximum	9.9	21.1	2.6	7.7	5.2	4.4
**mRNA Heavy chain**						
$C_{max,}$ µg/mL						
Mean	0.2	0.5	2.7	1.5	0.8	0.9
SD	0.2	0.4	0.9	0.8	0.4	0.3
CV%	78.4	86.6	32.1	53.7	45.5	29.5
Median	0.2	0.4	2.8	1.4	0.8	1.0
Minimum	0.1	0.2	1.6	0.5	0.3	0.5
Maximum	0.5	1.3	3.6	2.9	1.3	1.2
tmax, h						
Median	18.0	1.0	2.0	4.0	4.0	1.5
Minimum	2.0	1.0	1.0	2.0	1.0	1.0
Maximum	24.0	18.0	8.0	24.0	6.0	6.0
AUCO-last, µg*h/mL						
Mean	18.5	46.0	153	102	45.0	48.0
SD	15.6	36.8	16.4	36.0	23.8	13.8
CV%	84.1	80.0	10.7	35.2	52.9	28.9
Median	13.9	41.5	151	107	41.9	41.4
Minimum	5.31	6.7	137	41.3	21.1	34.9
Maximum	47.6	96.9	173	148	81.1	67.8
AUCO-168, µg*h/mL						
Mean	13.4	31.3	121	84.9	49.8	42.0
SD	11.4	27.3	18.7	33.6	23.2	14.4
CV%	85.2	87.1	15.4	39.6	46.5	34.3
Median	10.7	22.4	115	82.1	51.1	36.1
Minimum	4.9	5.0	107	27.9	22.7	28.9
Maximum	36.1	68.3	149	125	81.1	61.0
AUC0-inf, µg*h/mL						
Mean	18.9	46.5	154	102	60.1	NA
SD	15.8	37.2	16.8	36.0	29.7	NA
CV%	83.7	80.0	10.9	35.2	49.3	NA
Median	14.0	42.4	153	107	60.4	NA
Minimum	5.4	6.7	137	41.6	27.4	NA
Maximum	48.1	98.0	173	149	103	NA
$t_{1/2,}$ h						
Mean	85.9	85.0	79.6	67.2	64.8	60.7
SD	44.5	23.3	24.6	20.3	6.3	13.7
CV%	51.9	27.4	31.0	30.2	9.7	22.5
Median	82.8	87.7	77.2	71.2	63.1	63.9
Minimum	37.8	48.9	52.6	36.9	58.0	43.9
Maximum	150	117	111	89.9	74.9	77.6
CL, mL/h/kg						
Mean	5.8	10.2	2.6	4.6	4.1	4.4
SD	4.1	10.8	0.3	2.5	2.2	1.1
CV%	70.2	106.4	10.8	54.9	52.2	24.9
Median	4.8	5.9	2.6	3.7	3.3	4.8
Minimum	1.4	2.0	2.3	2.7	1.9	3.0
Maximum	12.4	30.0	2.9	9.6	7.3	5.7
**IAL**						
$C_{max,}$ µg/mL						
Mean	NA	7.1	15.3	16.3	5.8	8.0
SD	NA	2.6	7.8	4.6	1.3	4.0
CV%	NA	37.1	51.2	28.3	22.2	50.9
Median	NA	6.9	18.5	14.9	5.9	6.1
Minimum	NA	4.6	3.7	10.9	4.1	5.5

Maximum	NA	9.8	20.6	23.3	7.3	15.9
tmax, h						
Median	NA	1.0	1.0	1.0	1.0	1.0
Minimum	NA	0.5	0.5	0.5	0.5	0.5
Maximum	NA	1.1	3.0	1.0	1.0	1.0
AUCO-last, µg*h/mL						
Mean	9.5	20.7	28.6	7.7	10.1	NA
SD	4.1	5.0	9.1	1.2	4.1	NA
CV%	43.5	24.2	32.0	15.6	40.3	NA
Median	8.9	22.4	26.5	8.1	8.8	NA
Minimum	5.6	13.4	19.3	5.4	6.8	NA
Maximum	13.8	24.6	40.8	8.9	17.9	NA
AUCO-168, µg*h/mL						
Mean	NA	9.5	20.7	28.7	7.8	10.1
SD	NA	4.1	5.0	9.1	1.2	4.1
CV%	NA	43.4	24.3	31.7	15.6	40.1
Median	NA	8.9	22.4	27.0	8.1	8.8
Minimum	NA	5.7	13.4	19.4	5.4	6.9
Maximum	NA	13.8	24.6	40.8	8.91	17.9
AUC0-inf, µg*h/mL						
Mean	NA	9.5	20.7	28.7	7.8	9.5
SD	NA	4.1	5.0	9.1	1.2	4.1
CV%	NA	43.4	24.3	31.8	15.6	43.4
Median	NA	8.9	22.4	27.0	8.1	8.9
Minimum	NA	5.7	13.4	19.4	5.4	5.7
Maximum	NA	13.8	24.6	40.8	8.9	13.8
$t_{1/2,}$ h						
Mean	NA	7.97	6.67	13.3	8.05	9.8
SD	NA	3.14	2.26	3.41	3.23	2.6
CV%	NA	39.3	33.9	25.7	40.1	26.8
Median	NA	8.86	6.73	13.5	8.15	9.3
Minimum	NA	4.49	4.57	9.33	3.43	6.2
Maximum	NA	10.6	8.66	18.5	13.0	13.2
CL, mL/h/kg						
Mean	NA	369	313	232	404	334
SD	NA	162	96.2	71.0	80.7	98.5
CV%	NA	43.8	30.8	30.7	20.0	29.5
Median	NA	343	273	228	377	346
Minimum	NA	222	249	150	344	171
Maximum	NA	542	455	316	566	445

AUCO-inf, -last and 168= area under the concentration curve from time 0 extrapolated to infinity, from time 0 to the last measurable concentration, from time 0 to 168 days; AUECO-inf, -last and 168= area under the effect curve from time 0 extrapolated to infinity, from time 0 to the last measurable concentration, from time 0 to 168 days; CL=apparent clearance; C_{max}=maximum serum concentration; CV=coefficient of variance; E_{max}= maximum observed effect; NA=Not assessed; $t_{1/2}$=half-life; t_{max}= time to maximum observed serum concentration. TE_{max} = time to maximum observed effect. Participants received loratadine and ranitidine[a] or loratadine, ranitidine (sentinel, expansion) and acetaminophen (expansion)[b] or steroid (dexamethasone), and diphenhydramine and famotidine[c] or diphenhydramine and famotidine[d] 90 minutes prior to infusion. [e]Participants were administered two 0.3 mg/kg doses administered on days 1 and 8.

Fig. S1. Serum concentration of CHKV24 IgG, mRNA heavy and light chains of CHKV24 IgG and IAL for 0.6 mg/kg dose with and without steroid.

Mean serum concentration-time profiles of CHKV24 IgG following administration of single doses of 0.6 mg/kg and 0.6 mg/kg plus steroid during 366 days (a) and for mRNA (heavy and light chains) and IAL following single dose administration of 0.6 mg/kg plus steroid (dexamethasone) during 28 days (b). Error bars represent the standard error of each mean (SEM). Dotted line represents serum target concentration of 1 µg/mL antibody expected to provide protection against CHIKV infection. n=4 participants at each time point for the 0.6 mg/kg group and n=6 participants at each time point for the 0.6 mg/kg+steroid group examined over 366 days.

a.

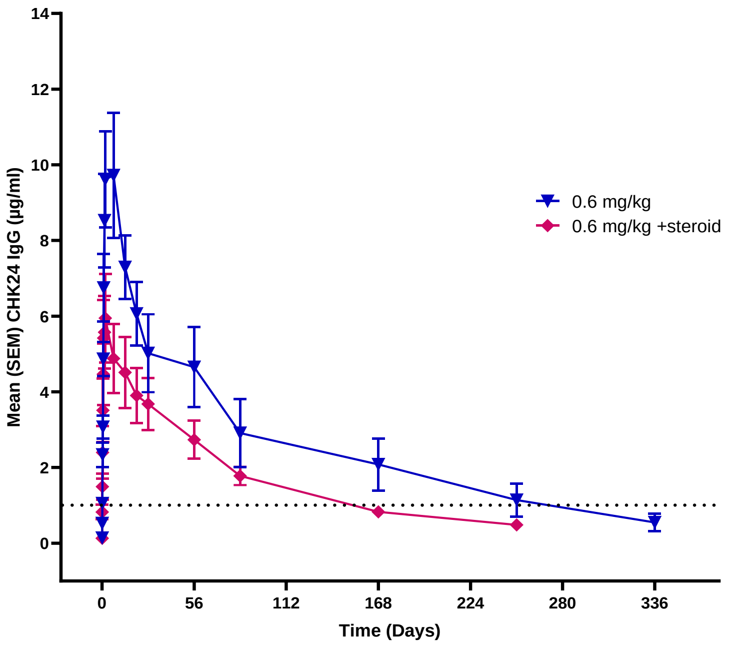

b.

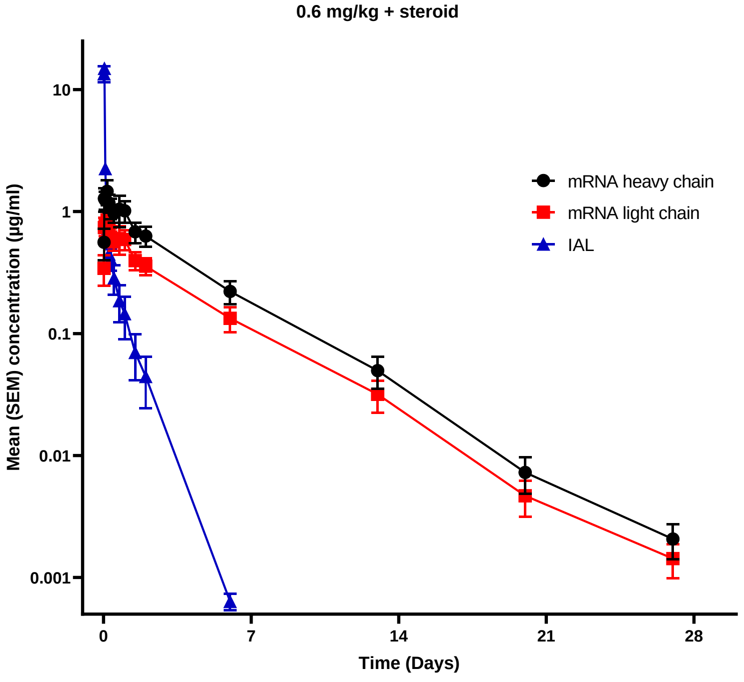

Figure S2. Dose-dependent neutralizing antibody titers in single-dose groups.
Serum neutralizing titers of CHKV24 IgG against CHIKV were assessed using the plaque reduction neutralization test (PRNT) assay at 12, 24 and 48 h following administration of 0.1, 0.3 and 0.6 mg/kg (without steroid) doses of mRNA-1944. Geometric mean titers (GMT) at the serum dilution at which CHIKV infection is reduced by 50% (ID50) and standard deviations are provided for each dose group and time point. n=6 participants in the 0.1 mg/kg and 0.3 mg/kg, and n=4 participants in the 0.6 mg/kg groups treated with mRNA-1944.

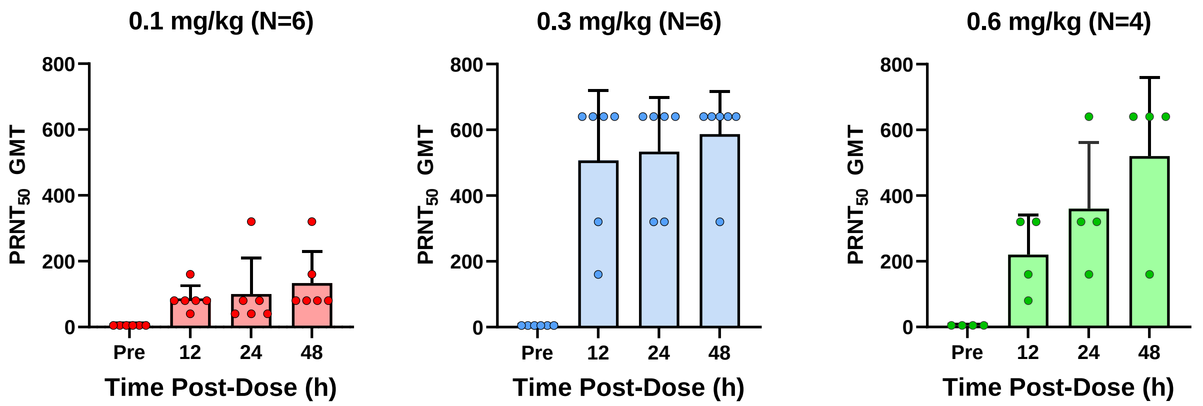

Fig. S3. Complement and Acute phase reactants

Serum concentration-time profiles of CRP and acute phase reactants of participants following administration of single doses in the 0.6 mg/kg (2 placebo, 1 mRNA-1944) group (a), and in the 0.6 mg/kg with steroid (2 placebo, 6 mRNA-1944) group (b) during 50 h, and 0.3 mg/kg 2-dose group (2 placebo, 6 mRNA-1944) group (c) during 216 hours. Profiles of participants on placebo are represented by dotted lines and those on mRNA-1944 by solid lines.

0. 6 mg/kg mRNA-1944

a.

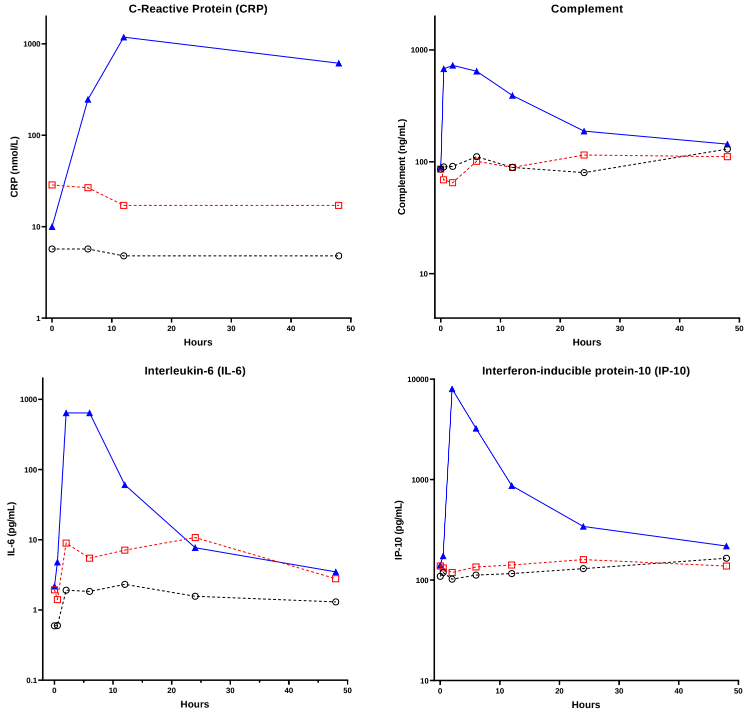

0. 6 mg/kg + steroid mRNA-1944

b.

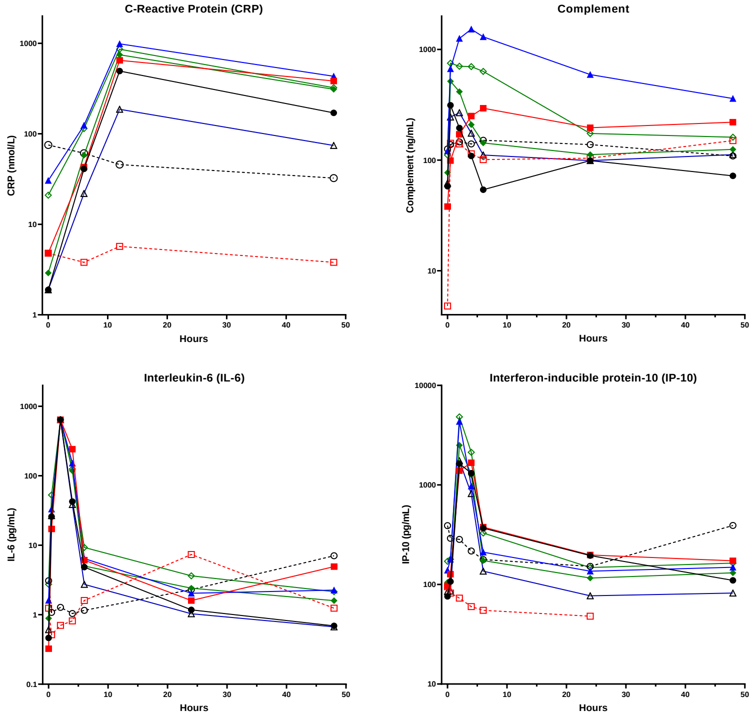

0.3 mg/kg 2-dose mRNA-1944

c.

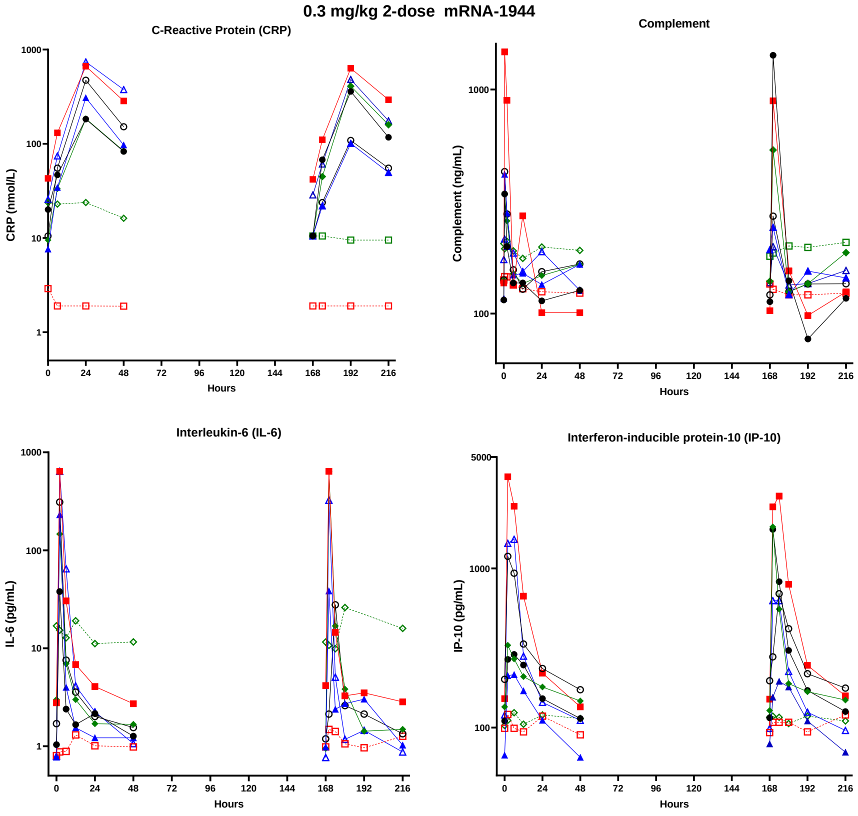

CLINICAL STUDY PROTOCOL

A PHASE 1, RANDOMIZED, PLACEBO-CONTROLLED, DOSE-RANGING STUDY TO EVALUATE THE SAFETY, TOLERABILITY, PHARMACOKINETICS, AND PHARMACODYNAMICS OF mRNA-1944, ENCODING FOR AN ANTI-CHIKUNGUNYA VIRUS MONOCLONAL ANTIBODY, IN HEALTHY ADULTS

PROTOCOL NO. mRNA-1944-P101

**Sponsor:**	*ModernaTX, Inc.*<br>*200 Technology Square*<br>*Cambridge, MA 02139*
**Sponsor Contact:**	*Allison August, MD*<br>*Senior Director, Clinical Development*<br>*Telephone: 617-209-5845*
**Version of Protocol:**	*7.0*
**Date of Protocol:**	*31 July 2020*

CONFIDENTIAL

The concepts and information contained in this document or generated during the study are considered proprietary and may not be disclosed in whole or in part without the expressed, written consent of *ModernaTX, Inc.*

The study will be conducted according to the International Council for Harmonisation harmonised tripartite guideline E6(R2): Good Clinical Practice.

Signature Page

**PROTOCOL TITLE:**	A Phase 1, Randomized, Placebo-Controlled, Dose-Ranging Study to Evaluate the Safety, Tolerability, Pharmacokinetics, and Pharmacodynamics of mRNA-1944, Encoding for an Anti-Chikungunya Virus Monoclonal Antibody, in Healthy Adults
**PROTOCOL NUMBER:**	*mRNA-1944-P101*

Tal Zaks, MD, PhD<br>*Chief Medical Officer*<br>ModernaTX, Inc.	Date
Allison August, MD<br>*Senior Director, Clinical Development*<br>*ModernaTX, Inc.*	Date

Investigator Protocol Agreement Page

I agree to conduct the study as outlined in the protocol titled "A Phase 1, Randomized, Placebo-Controlled, Dose-Ranging Study to Evaluate the Safety, Tolerability, Pharmacokinetics, and Pharmacodynamics of mRNA-1944, Encoding for an Anti-Chikungunya Virus Monoclonal Antibody, in Healthy Adults" in accordance with all applicable government regulations and International Council for Harmonisation (ICH) guidelines.

____ ____________________________

Signature of Investigator Date

____

Printed Name of Investigator

Table of Contents

Title Page .... Error! Bookmark not defined.
Signature Page 15
Investigator Protocol Agreement Page 16
Table of Contents 17
List of Tables 21
List of Figures 21
AMENDMENT 6.0 22
AMENDMENT 5.0 22
AMENDMENT 4.0 23
AMENDMENT 3.0 26
AMENDMENT 2.0 28
AMENDMENT 1.0 32
Protocol Synopsis 33
1. Schedule of Events 48
1.1 Dose Level Cohorts 1 Through 6 48
1.2 Dose Level Cohort 7 (0.3 Mg/Kg × 2 Doses, Administered at a 7-Day Interval) 52
2. Introduction 56
2.1 Background 56
2.2 Nonclinical Studies in Development of mRNA-1944 57
2.3 Clinical Studies with mRNA-1944 59
2.4 Rationale for Study and Study Design 59
2.4.1 Rationale for Study 59
2.4.2 Rationale for Study Population and Design 59
2.4.3 Rationale for Dose Selection 59
3. Study Objectives 60
3.1 Primary Objective 60
3.2 Secondary Objectives 60
3.3 Exploratory Objectives 60
4. Investigational Plan 60
4.1 Study Design 60
4.2 Dosing 66
4.2.1 Sentinel Dosing Strategy 66
4.2.2 Internal Safety Team and Safety Monitoring Committee 67
4.2.3 Dose Expansion 68
4.2.4 Dose Escalation 68
4.2.5 Pause Rules 68
4.3 End of Study Definition 69
5. Study Population 69

5.1 Inclusion Criteria 69
5.2 Exclusion Criteria 70
5.3 Withdrawal of Subjects from the Study 72
5.3.1 Reasons for Withdrawal 72
5.3.2 Handling of Withdrawals 72
5.3.3 Replacements 73
6. Study Treatments 73
6.1 Treatments Administered 73
6.1.1 Dose Modifications 74
6.2 Investigational Products 74
6.2.1 Study Drug Packaging and Storage 75
6.2.2 Study Drug Accountability 75
6.3 Method of Assigning Subjects to Treatment Groups 76
6.4 Blinding 76
6.4.1 Blinding Procedures 76
6.4.2 Breaking the Blind 77
6.5 Treatment Compliance 77
6.6 Prior and Concomitant Medications 77
6.6.1 Prior Medications 77
6.6.2 Concomitant Medications 78
7. Study Procedures 78
7.1 Pharmacokinetic Assessments and Endpoints 78
7.1.1 Pharmacokinetic Sample Collection 79
7.2 Pharmacodynamic Assessments and Endpoints 79
7.2.1 Pharmacodynamic Sample Collection 79
7.3 Exploratory Assessments and Endpoints 80
7.4 Safety Assessments and Endpoints 80
7.4.1 Adverse Events 80
7.4.1.1 Adverse Event Definitions 80
7.4.1.2 Serious Adverse Event Definitions 81
7.4.1.3 Adverse Events of Special Interest 81
7.4.1.4 Pregnancy 82
7.4.1.5 Eliciting and Documenting Adverse Events 82
7.4.1.6 Reporting Adverse Events 83
7.4.1.7 Assessment of Severity 83
7.4.1.8 Assessment of Causality 84
7.4.1.9 Follow-Up of Adverse Events 84
7.4.2 Infusion-Related Reaction Assessments 85
7.4.3 Clinical Laboratory Testing 85
7.4.4 Vital Sign Measurements 86

7.4.5 12-Lead Electrocardiograms 87
7.4.6 Physical Examinations 87
8. Statistical Analysis Plans, Data analysis, and statistical methods 88
8.1 Sample Size Calculations 88
8.2 Analysis Sets 88
8.3 Statistical Analysis 88
8.3.1 Pharmacokinetic Analyses 89
8.3.2 Pharmacodynamic Analyses 89
8.3.3 Exploratory Analysis 90
8.3.4 Safety Analyses 90
8.4 Handling of Missing Data 90
8.5 Periodic Pharmacokinetic/Pharmacodynamic Analyses 91
8.6 Interim Analyses 91
9. Reference List 92
10. Appendices 94
10.1 Appendix 1: List of Abbreviations 94
10.2 Appendix 2: Study Governance 96
10.2.1 Data Quality Assurance 96
10.2.2 Investigator Obligations 96
10.2.2.1 Confidentiality 96
10.2.2.2 Institutional Review 96
10.2.2.3 Subject Consent 97
10.2.2.4 Study Reporting Requirements 97
10.2.2.5 Financial Disclosure and Obligations 97
10.2.2.6 Investigator Documentation 98
10.2.2.7 Study Conduct 98
10.2.2.8 Case Report Forms and Source Documents 98
10.2.2.9 Adherence to Protocol 99
10.2.2.10 Reporting Adverse Events 99
10.2.2.11 Investigator’s Final Report 99
10.2.2.12 Records Retention 99
10.2.2.13 Publications 99
10.2.3 Study Management 100
10.2.3.1 Monitoring 100
10.2.3.2 Management of Protocol Amendments and Deviations 100
10.2.3.3 Study Termination 101
10.2.3.4 Final Report 102
10.3 Appendix 3: Glossary of Terms 103
10.4 Appendix 4: Toxicity Grading Scale Tables 103

10.5 Appendix 5: Second Symposium on the Definition and Management of Anaphylaxis Summary Report - Second National Institute of Allergy and Infectious Disease/Food Allergy and Anaphylaxis Network Symposium 109

List of Tables

Table 10-1: Tables for Clinical Abnormalities - Vital Signs Only 104
Table 10-2: Study-Specific Normal Ranges and Associated Laboratory Abnormalities .. 105
Table 10-3: Study-Specific Normal Ranges - Hematology 107
Table 10-4: Study-Specific Normal Ranges - Urine 108
Table 10-5: Clinical Criteria for Diagnosing Anaphylaxis 110

List of Figures

Figure 4-1 Study Flow Diagram 61

AMENDMENT 6.0

Changes to the protocol

Clarified that interim analyses henceforth will be unblinded as to the subject level treatment assignment (Section 8.6). Added language to specify that a final analysis will be performed at end of study following database lock (Section 8.5)

AMENDMENT 5.0

Changes to the protocol

Editorial changes were made throughout the protocol.

Throughout the protocol a dose level cohort 7 was added. The dose level cohort 7 will be administered two infusions of 0.3 mg/kg, one infusion administered on Day 1 and the second infusion administered on Day 8 to assess comparability in protein exposure and tolerability as compared to a single 0.6 mg/kg dose. Sentinel dosing will be utilized. The IST will review safety data on each sentinel subject approximately 48 hours after receipt of the first 0.3 mg/kg dose and approve receipt of the second 0.3 mg/kg dose on Day 8.

The IST will review safety data approximately 48 hours after each sentinel subject receives their second 0.3 mg/kg dose to approve dosing of the subsequent sentinel subject (second and third sentinel subjects).

The IST will also review safety data on all sentinel subjects approximately 48 hours after the third sentinel subject receives their second dose and approve dosing of the expansion group (5 subjects).

The 5 expansion subjects will be dosed in parallel. The IST will review safety data approximately 48 hours after all 5 expansion subjects receive their first 0.3 mg/kg dose to approve dosing of the second 0.3 mg/kg dose.

Cohort 7 may run in parallel with dose level cohort 5, regardless of whether dosing has begun in cohort 6.

All subjects in dose level cohort 7 will receive the premedication regimen of diphenhydramine (50 mg, oral) and famotidine (20 mg, oral).

Cohort was updated to dose level cohort throughout the protocol.

The number of subjects was increased from 48 to 56.

Clarified that because 1 subject in the 0.6 mg/kg dose level cohort (after receiving the oral premedication regimen) developed a persistent grade 2 or higher IRR all subsequent subjects in the study who receive >0.3 mg/kg as a single dose will be administered dexamethasone (10 mg, IV) in addition to the premedication regimen in the following sections:

- PROTOCOL SYNOPSIS, STUDY DESIGN

- SECTION 4.1, STUDY DESIGN
- SECTION 6.6.2, Concomitant Medications

A separate schedule of events for dose level 7 was created in SECTION 1.2.

AMENDMENT 4.0

Changes to the protocol

Editorial changes were made throughout the protocol.

Added "to evaluate complement and acute phase reactant parameters" as an exploratory objective and removed "complement and acute phase reactants" from the safety assessment in the following sections:

- PROTOCOL SYNOPSIS, OBJECTIVES
- PROTOCOL SYNOPSIS, STUDY PROCEDURES, Exploratory Assessments and Endpoints
- PROTOCOL SYNOPSIS, STUDY PROCEDURES, Safety Assessments and Endpoints
- SECTION 3.3, EXPLORATORY OBJECTIVE
- SECTION 7.3, EXPLORATORY ASSESSMENTS AND ENDPOINTS
- SECTION 7.4, SAFETY ASSESSMENTS AND ENDPOINTS
- SECTION 8.3.3, Exploratory Analysis

Study flow diagram was updated to reflect 6 dose levels in the following sections:

- PROTOCOL SYNOPSIS, STUDY DESIGN
- SECTION 4.1, STUDY DESIGN, FIGURE 4-1 STUDY FLOW DIAGRAM

Updated to reflect 6 dose level cohorts instead of 4 dose level cohorts; 0.45 mg/kg dose level cohort was added, dose level cohorts were updated to 0.45 mg/kg for dose level cohort 4 (optional cohort), 0.6 mg/kg for dose level cohort 5, and 1.0 mg/kg for dose level cohort 6, and the number of subjects was updated from 32 to 48 in the following sections:

- PROTOCOL SYNOPSIS, STUDY DESIGN
- PROTOCOL SYNOPSIS, STATISTICAL ANALYSIS PLANS, Sample Size
- SECTION 2.4.3, Rationale for Dose Selection
- SECTION 4.1, STUDY DESIGN
- SECTION 4.2.3, Dose Expansion
- SECTION 5, STUDY POPULATION

- SECTION 6.1, TREATMENTS ADMINISTERED
- SECTION 6.3, METHOD OF ASSIGNING SUBJECTS TO TREATMENT GROUPS
- SECTION 8.1, SAMPLE SIZE CALCULATIONS

Additional information regarding expansion subject enrollment for dose level cohorts 5 and 6 (and optional dose level cohort 4), the fact that dose level cohort 4 is an optional intermediate dose level cohort, and clarification on when the internal safety team will meet were added to the following sections:

- PROTOCOL SYNOPSIS, STUDY DESIGN
- PROTOCOL SYNOPSIS, STUDY DESIGN, Internal Safety Team and Dose Expansion
- SECTION 4.1, STUDY DESIGN
- SECTION 4.2.2, Internal Safety Team and Safety Monitoring Committee

Clarification of which dose level cohort received or will receive dexamethasone premedication, the dose amount, and the reason why dexamethasone was added to dose level cohorts 5 and 6 (and optional dose level cohort 4), to the following sections:

- PROTOCOL SYNOPSIS, STUDY DESIGN
- SECTION 1, SCHEDULE OF EVENTS TABLE, footnote “l”
- SECTION 4.1, STUDY DESIGN
- SECTION 6.1, TREATMENTS ADMINISTERED
- SECTION 6.6.2, Concomitant Medications

Decreased the total infusion time from 3 hours to 1 hour in the following sections:

- PROTOCOL SYNOPSIS, STUDY DESIGN
- PROTOCOL SYNOPSIS, STUDY TREATMENTS
- SECTION 4.1, STUDY DESIGN
- SECTION 6.1, TREATMENTS ADMINISTERED

Added dexamethasone (10 mg, IV; dose level cohorts 5 and 6 [optional dose level cohort 4]) to the list of premedications, replaced loratadine (10 mg, oral) with diphenhydramine (50 mg, oral) and replaced ranitidine (150 mg, oral) with famotidine (20mg, oral) in the list of premedications received on Day 1, removed acetaminophen 650 mg oral, and clarified which dose level cohort received which premedication regimen in the following sections:

- PROTOCOL SYNOPSIS, STUDY DESIGN
- SECTION 1, SCHEDULE OF EVENTS TABLE, footnote "l"
- SECTION 4.1, STUDY DESIGN
- SECTION 6.6.2, Concomitant Medications

Updated and edited existing text to "Because 1 subject (after receiving the oral premedication regimen) developed a persistent grade 2 or higher IRR, all subsequent subjects in the study will be administered dexamethasone (10 mg, IV), in addition to the above oral regimen, as premedication prior to the study drug infusion." regarding the management of infusion-related reactions in the following sections:

- PROTOCOL SYNOPSIS, STUDY DESIGN
- SECTION 4.1, STUDY DESIGN

Added "(and cardiac enzymes when obtained per protocol)" to the safety assessments and safety data in the following sections:

- PROTOCOL SYNOPSIS, STUDY DESIGN
- PROTOCOL SYNOPSIS, STUDY PROCEDURES, Safety Assessments and Endpoints
- PROTOCOL SYNOPSIS, STASTICAL ANALYSIS PLANS, Safety Analyses
- SECTION 4.1, STUDY DESIGN
- SECTION 6.4.1, Blinding Procedures
- SECTION 7.4, SAFETY ASSESSMENTS AND ENDPOINTS
- SECTION 7.4.1.5, Eliciting and Documenting Adverse Events
- SECTION 8.3.4, Safety Analyses
- SECTION 8.6, INTERIM ANALYSES
- SECTION 10.2.3.2.1, Modification of the Protocol

Updated the blood sample collection time points for serum mRNA encoding for the CHIKV24 IgG and plasma IAL and serum (mRNA expressed) CHIKV24 IgG to separate all subjects in dose level cohorts 1, 2, 4, 5, and 6 and the sentinel subjects in dose level cohort 3 from the 3 expansion subjects in dose level cohort 3 in the following sections:

- PROTOCOL SYNOPSIS, STUDY DESIGN
- SECTION 1, SCHEDULE OF EVENTS TABLE, footnotes "n" and "o"
- SECTION 4.1, STUDY DESIGN

Updated 4 dose levels to 6 dose levels of mRNA-1944 in the following sections:

- PROTOCOL SYNOPSIS, STUDY DESIGN, Safety Monitoring Committee and Dose Escalation
- SECTION 4.2.4, Dose Escalation

Lowered the upper weight limit from 100 kg to ≤ 90 kg and added a maximum body mass index of ≤ 33 kg/m^2 for inclusion criteria 2 in the following sections:

- PROTOCOL SYNOPSIS, STUDY POPULATION, Inclusion Criteria
- SECTION 5.1, INCLUSION CRITERIA

Added "If a clinically significant change from screening is noted on the ECG, blood samples for the assessment of cardiac enzymes (troponin I and creatine kinase-MB) will be drawn within 1 hour and at 6 and 24 hours after the noted abnormal electrocardiogram." to the following sections:

- SECTION 1, SCHEDULE OF EVENTS TABLE, footnote "e"
- SECTION 7.4.5, 12-Lead Electrocardiograms

Clarified when subjects in the expansion group of dose level cohorts 5 and 6 (and optional dose level cohort 4) will be enrolled in the follow section:

- SECTION 4.2.3, Dose Expansion

Added time parameter to the collection of Infusion Related Reactions (IRR), "will include reactions assessed as related to study drug occurring during or within 24 hours after the infusion" to the following sections:

- PROTOCOL SYNOPSIS, STUDY DESIGN
- SECTION 4.1, STUDY DESIGN

AMENDMENT 3.0

Changes to the protocol

Editorial changes were made throughout the protocol.

The exploratory objectives were revised to delete the evaluation of the genotype and concentration levels of apolipoprotein E (Apo E) and to delete the language that specifies "half

maximal inhibitory concentration (IC_{50})" when evaluation of the in vitro serum neutralizing antibody titer is performed and IC_{50} abbreviation was removed in the following sections:

- PROTOCOL SYNOPSIS, OBJECTIVES
- SECTION 1, SCHEDULE OF EVENTS TABLE, abbreviation, and footnote "r"
- SECTION 3.3, EXPLORATORY OBJECTIVES
- SECTION 10, APPENDIX 1, LIST OF ABBREVIATION

Increased the total infusion time from 1 hour to 3 hours and revised the text to indicate that infusion time may be further extended due to the occurrence of an adverse reaction assessed as related to infusion of study drug experienced by any subject in a given dose level cohort in the following sections:

- PROTOCOL SYNOPSIS, STUDY DESIGN
- SECTION 4.1, STUDY DESIGN

Blood sample collection times modified to correspond to lengthened total infusion time in the following sections:

- PROTOCOL SYNOPSIS, STUDY DESIGN
- SECTION 1, SCHEDULE OF EVENTS TABLE, abbreviation, and footnote "n" and "o"
- SECTION 4.1, STUDY DESIGN

The sentence in the protocol synopsis and study design section that specifies "The Sponsor may stop further dose escalation once pharmacologic goals have been achieved" was reiterated in the full protocol in the following section:

- SECTION 4.2.4, DOSE ESCALATION

The time point for the analyses of the periodic PK and PD analyses was clarified to specify data through at least 7 days after study drug infusion in the following section:

- SECTION 8.5, PERIODIC PHARMACOKINETIC/PHARMACODYNAMIC ANALYSES

The data used for the interim safety analyses was further specified in the following section:

- SECTION 8.6, INTERIM ANALYSES

Details regarding the category of AE to be captured and the length of time for which each category of AE is to be captured that is outlined in Section 7.4.1.5 of the protocol was reiterated in footnote "t" of Section 1, Schedule of Events. The study-specific normal ranges and associated laboratory abnormalities table was added into Section 10.4, Appendix 4 Toxicity

Grading Scale Tables and text was updated to reflect that the toxicity grading scales for laboratory abnormalities are presented in Table 10-2.

Acetaminophen (650 mg, oral) was added to the premedication list in the following sections:

- PROTOCOL SYNOPSIS, STUDY DESIGN
- SECTION 1, SCHEDULE OF EVENTS TABLE, abbreviation, and footnote “l”
- SECTION 4.1, STUDY DESIGN
- SECTION 6.6.2, CONCOMITANT MEDICATIONS

The specifications for enrollment of the five subjects in the expansion group of each dose level cohort was revised to “subjects in the expansion group of each dose level cohort level will be enrolled with a minimum of 24 hours between each of the 5 subjects for dose level cohorts 3 and 4.” in the following sections:

- PROTOCOL SYNOPSIS, STUDY DESIGN
- SECTION 4.1, STUDY DESIGN
- SECTION 4.2.3, DOSE EXPANSION

The addition of complement and acute phase reactants to the safety assessments in the following sections:

- PROTOCOL SYNOPSIS, SAFETY ASSESSMENTS AND ENDPOINTS
- SECTION 7.4, SAFETY ASSESSMENTS AND ENDPOINTS
- SECTION 7.4.3, CLINICAL LABORATORY TESTING
- LIST OF ABBREVIATIONS
- SECTION 1, SCHEDULE OF EVENTS TABLE, abbreviation, and footnote “u” and “v”

AMENDMENT 2.0

Changes to the protocol

Editorial changes were made throughout the protocol.

The pharmacokinetics of mRNA1944 were revised to include mRNA encoding for CHIKV24 IgG and IAL and to clarify, where needed, serum concentrations of mRNA encoding for CHIKV24 IgG and plasma concentrations of IAL in the following sections:

- PROTOCOL SYNOPSIS, OBJECTIVES
- PROTOCOL SYNOPSIS, STUDY PROCEDURES, Pharmacokinetic Assessments and Endpoints

- PROTOCOL SYNOPSIS, STATISTICAL ANALYSIS PLANS, Analysis Sets
- SECTION 1, SCHEDULE OF EVENTS TABLE and footnote “n”
- SECTION 3.2, SECONDARY OBJECTIVES
- SECTION 3.3, EXPLORATORY OBJECTIVES
- SECTION 4.1, STUDY DESIGN
- SECTION 7.1, PHARMACOKINETIC ASSESSMENT AND ENDPOINTS
- SECTION 8.2, ANALYSIS SETS
- SECTION 8.3.1, Pharmacokinetic Analyses
- SECTION 8.3.3, Exploratory Analysis

Blood sample collection window added to mRNA-encoding for the CHIKV24 IgG and IAL samples to PROTOCOL SYNOPSIS, STUDY DESIGN

“A subject may be rescreened for study eligibility if their originally intended sentinel or expansion group is filled and their 28-day screening window is surpassed before another group opens. The subject will be assigned a new screening number and all screening procedures will be repeated. Subjects who did not meet all enrollment criteria at their first screening will not be allowed to rescreen. Screen failures are defined as subjects who sign the consent form but who are not subsequently randomly assigned to the study intervention or entered in the study. Information on eligibility, demographics, serious adverse events (SAEs), and informed consent will be collected for all screen failures.” was added to the following sections:

- PROTOCOL SYNOPSIS, STUDY DESIGN
- SECTION 4.1 STUDY DESIGN

Addition of the following language: Note: if the total infusion time is extended beyond 1 hour the blood sample should be collected at EOI; and if it overlaps with pre-specified timepoints sampling should continue at the next scheduled timepoint relative to the start of infusion.

- PROTOCOL SYNOPSIS, STUDY DESIGN
- SECTION 4.1, STUDY DESIGN

"Blood sample for the determination of in vitro serum neutralizing antibody titer (IC_{50}) against a clinically relevant strain of CHIKV will be collected within 60 minutes before study drug infusion; and at" was added to the following sections:

- PROTOCOL SYNOPSIS, STUDY DESIGN
- SECTION 1, SCHEDULE OF EVENTS TABLE and footnote "r"
- SECTION 4.1, STUDY DESIGN

Removal of "All study time points are relative to the start of the study drug infusion" in the following sections:

- PROTOCOL SYNOPSIS, STUDY DESIGN
- SECTION 4.1, STUDY DESIGN

Safety assessments will now include prior and concomitant medication in the following sections:

- PROTOCOL SYNOPSIS, STUDY DESIGN
- PROTOCOL SYNOPSIS, STUDY PROCEDURES, Safety Assessments and Endpoints
- SECTION 4.1, STUDY DESIGN
- SECTION 7.4, SAFETY ASSESSMENTS AND ENDPOINTS

Text to indicate that a blood sample for DNA isolation in order to determine genotyping for apolipoprotein E will be collected from subjects who have consented to participate in the genetic analysis component of the study, that participation is optional, and to add the blood sample will be collected within 60 minutes before study drug infusion was added, as needed, to the following sections:

- PROTOCOL SYNOPSIS, STUDY DESIGN
- PROTOCOL SYNOPSIS, STUDY PROCEDURES, Exploratory Assessments and Endpoints
- SECTION 3.3, EXPLORATORY OBJECTIVES
- SECTION 4.1, STUDY DESIGN
- SECTION 7.3, EXPLORATORY ASSESSMENTS AND ENDPOINTS

Addition of new vital sign time point of 60 minutes before study drug infusion on Day 1 to SECTION 1, SCHEDULE OF EVENTS TABLE footnote "d"

Language added concerning the use of retained serum/plasma samples for future analysis to SECTION 4.1, STUDY DESIGN

Removal of the language for the characterization of mRNA expressed antibody including FC array, antibody-dependent cell-mediated cytotoxicity (ADCC) activity, and glycosylation from the following sections:

- PROTOCOL SYNOPSIS, OBJECTIVES
- PROTOCOL SYNOPSIS, STUDY DESIGN
- PROTOCOL SYNOPSIS, STUDY PROCEDURES, Exploratory Assessments and Endpoints
- SECTION 1, SCHEDULE OF EVENTS, abbreviation, and footnote
- SECTION 3.3, EXPLORATORY OBJECTIVES
- SECTION 4.1, STUDY DESIGN
- SECTION 7.3, EXPLORATORY ASSESSMENTS AND ENDPOINTS
- SECTION 10.1, APPENDIX 1: LIST OF ABBREVIATIONS

Add placebo language in SECTION 6.2.2, Study Drug Accountability

Removal of Table 7.1 Total Blood Volume, footnotes, and the reference to the table in SECTION 7, STUDY PROCEDURES.

Added “and baseline” to Exclusion Criteria 5 in the following sections:

- PROTOCOL SYNOPSIS, STUDY POPULATION, Exclusion Criteria 5
- SECTION 5.2 EXCLUSION CRITERIA 5

Added “a seasonal influenza vaccine is permissible.” to Exclusion Criteria 8 in the following sections:

- PROTOCOL SYNOPSIS, STUDY POPULATION, Exclusion Criteria 5
- SECTION 5.2 EXCLUSION CRITERIA 5

Physical examination was added to Day 7 in SECTION 1, SCHEDULE OF EVENTS

“Prior and concomitant medication” added to the following sections:

- SECTION 6.4.1 Blinding Procedures
- SECTION 8.6, INTERIM ANALYSES

Addition of symptoms of hypersensitivity to SECTION 7.4.1.3, Adverse Events of Special Interest.

Vital signs will be graded using the toxicity grading scale for healthy adult and adolescent volunteers enrolled in preventative vaccine clinical trials; tables for clinical abnormalities (DHHS 2007) was added to SECTION 7.4.1.7, Assessment of Severity.

Addition of "serum" to SECTION 8.4, HANDLING OF MISSING DATA.

Change the collection of adverse events from time of the first study drug infusion of Day 1 to "from the time of informed consent" in the following sections:

- SECTION 1, SCHEDULE OF EVENTS and footnote "t"
- SECTION 7.4.1, Adverse Events
- SECTION 7.4.1.5, Eliciting and Documenting Adverse Events.

Addition of SECTION 8.5, PERIODIC PHARMACOKINETIC/PHARMACODYNAMIC ANALYSES.

Text added regarding when periodic PK and PD analysis will be performed to SECTION 8.6, INTERIM ANALYSIS.

Vaccination was updated to "study drug infusion" in the definition of Grade 1 laboratory abnormality without potential clinical significance in APPENDIX 3: GLOSSARY OF TERMS.

Text added to clarify toxicity grading of clinical and laboratory abnormalities. Clinical and laboratory abnormalities was removed from Table 10.1 (page 76). Table 10.1 was updated to specify "Vital Signs Only" in APPENDIX 4: TOXICITY GRADING SCALE TABLES.

AMENDMENT 1.0

Changes to the protocol

Maximum infusion time changed from 6 hours to 4 hours in the following sections:

- PROTOCOL SYNOPSIS, STUDY DESIGN
- SECTION 4.1, STUDY DESIGN
- SECTION 6.1.1, Dose Modifications

Protocol Synopsis

PROTOCOL NO.: *mRNA-1944-P101*

TITLE: A Phase 1, Randomized, Placebo-Controlled, Dose-Ranging Study to Evaluate the Safety, Tolerability, Pharmacokinetics, and Pharmacodynamics of mRNA-1944, Encoding for an Anti-Chikungunya Virus Monoclonal Antibody, in Healthy Adults

STUDY PHASE: 1

STUDY SITE: *1* study site

OBJECTIVES:

The primary objective of this study is to evaluate the safety and tolerability of escalating doses of mRNA-1944 administered via intravenous (IV) infusion in subjects 18 to 50 years of age.

The secondary objectives of this study are the following:

- To determine the pharmacokinetics (PK) of mRNA encoding for CHIKV24 immunoglobulin (IgG) (heavy and light chain mRNA) and IAL
- *To determine the pharmacodynamics (PD) of mRNA-1944 as assessed by chikungunya virus (CHIKV24) IgG*

The exploratory objectives of this study are the following:

- To evaluate the formation of anti-polyethylene glycol (PEG) antibodies
- To evaluate the formation of anti-CHIKV24 IgG antibodies
- To evaluate the impact of several baseline characteristics on the PK/PD of mRNA-1944 of mRNA encoding for CHIKV24 IgG and IAL
- To evaluate the in vitro serum neutralizing antibody titer against a clinically relevant strain of CHIKV
- To evaluate complement and acute phase reactant parameters

STUDY DESIGN:

This is a Phase 1, first-in-human, single-center, randomized, investigator-blinded, placebo-controlled, dose-escalation study to evaluate the safety, tolerability, PK, and PD (CHIKV24 IgG) of mRNA-1944 in healthy adult subjects. Investigator blind means the investigator, study subjects, site monitors, and study site personnel will be blinded to the study drug administered with the following exceptions: the 3 sentinel subjects in each dose level are not blinded, unblinded pharmacy personnel, unblinded study monitor, unblinded clinical trial manager, and an unblinded team to provide safety data to the SMC.

The study flow diagram is presented below.

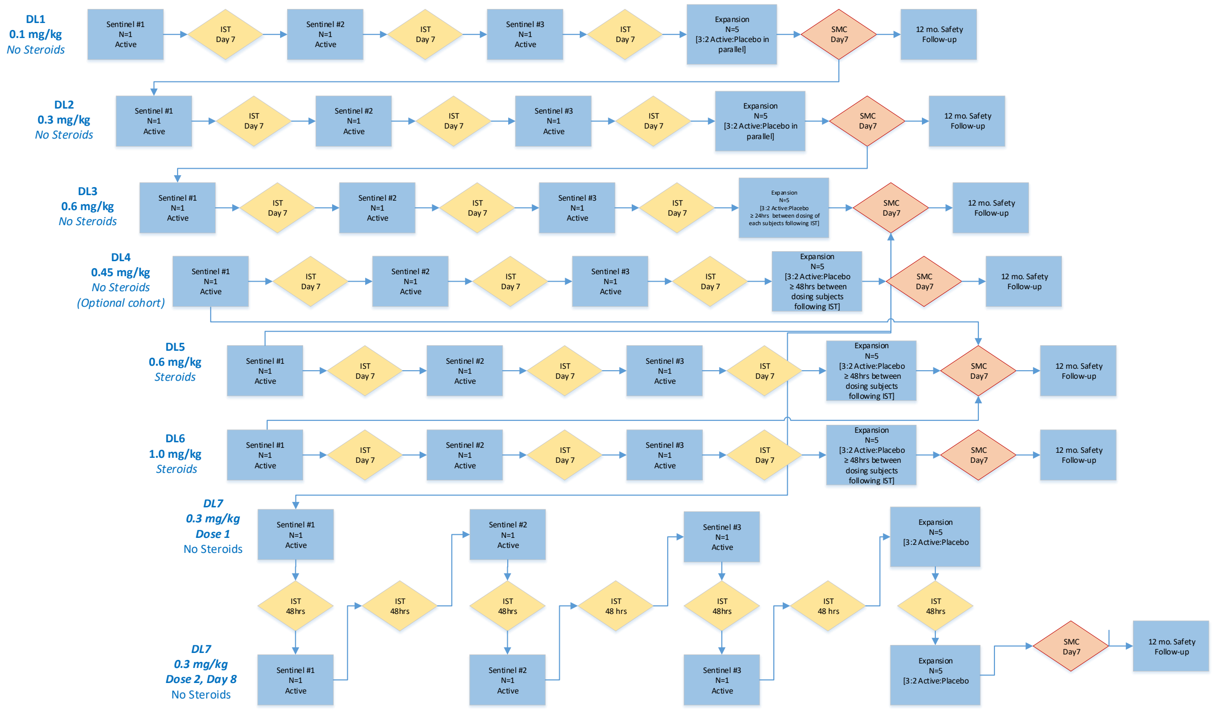

Abbreviations: DL, dose level; hrs, hours; IST, internal safety team; mo, month; SMC, safety monitoring committee.

Approximately 56 subjects (8 per cohort) are planned to be enrolled (including the optional intermediate dose level cohort of 0.45 mg/kg); 6 subjects will receive mRNA-1944 and 2 subjects will receive placebo within each cohort.

Among the 6 subjects receiving mRNA-1944, 3 are the sentinel subjects. The remaining 5 subjects within each dose level cohort will be randomly assigned 3:2 to receive mRNA-1944 or placebo.

Subjects in the expansion group of dose level cohorts 5 and 6 (and optional dose level cohort 4) will be enrolled with a minimum of 48 hours between the 5 subjects. For expansion subject enrollment in dose level cohorts 5 and 6 (and optional dose level cohort 4), the internal safety team (IST) will meet approximately 48 hours after each subject in the expansion group completes their infusion to review the clinical course and safety data of the immediate post-infusion period and to approve the dosing of the next expansion cohort subject. Subjects in the expansion group of dose level 7 will be enrolled and dosed in parallel.

Seven cohorts of mRNA-1944 are planned to be investigated in a dose-escalation manner; one of the dose level cohorts, the 0.45 mg/kg dose level cohort, is an optional intermediate dose level cohort. The first 3 dose level cohorts are doses of 0.1, 0.3, and 0.6 mg/kg and these cohorts are dosed without dexamethasone included in the premedication regimen. In order to enable a more robust characterization of the PK, PD, and adverse reaction profile and to ensure that all subjects in a complete dose level cohort receive the identical premedication regimen, a second dose level cohort at 0.6 mg/kg has been added. This dose level cohort will receive the prespecified premedication regimen including criteria of when steroids should be added. Dexamethasone premedication (10 mg, IV) will be added to the premedication regimen for all

subjects in the 0.6 mg/kg and 1.0 mg/kg dose level cohorts (and the 0.45 mg/kg optional dose level cohort).

Subjects in dose level cohort 7 will be administered two IV infusions of 0.3 mg/kg, one infusion on Day 1 and another subsequent infusion on Day 8. The premedication regimen for dose level cohort 7 will include diphenhydramine (50 mg, oral) and famotidine (20 mg, oral) and will be administered prior to the planned start of study drug infusion on Days 1 and 8. Dexamethasone will not be included in the premedication regimen for dose level cohort 7.

Study drug will be administered to dose level cohorts 1, 2, 4, 5, 6, and 7 and the sentinel subjects in dose level cohort 3 as a single IV infusion over 1 hour by a controlled infusion device. Study drug will be administered to the expansion subjects in dose level cohort 3 as a single IV infusion over approximately 3 hours by a controlled infusion device. The infusion time may be further extended up to 4 hours in the event of an infusion reaction or the occurrence of an adverse reaction assessed as related to infusion of the study drug experienced by any subject in a given cohort.

Subjects in dose level cohorts 1 through 6 will remain inpatient at the study site to be observed for safety assessments and PK/PD sampling for 48-hours following completion of dosing on Day 1. Subjects in dose level cohort 7 will remain inpatient at the study site from Day –1, prior to the first dose of the 2-dose regimen, through 48-hours following completion of the second dose on Day 8. All subjects in dose level cohort 7 will be observed for safety assessments and undergo PK/PD sampling for 48-hours following completion of dosing of each of the 0.3 mg/kg doses. Adverse events (AEs) will be captured through the study follow-up period of 12 months and additional plasma PK samples will be collected through end of study (Week 52; 12 months). Optional dose level cohorts for intermediate dose levels may be added for de-escalation of the dose after review of cumulative data by the safety monitoring committee (SMC). The Sponsor may stop further dose escalation once pharmacologic goals have been achieved.

Consent and screening will occur over the 28-day period before Day 1. All subjects will be screened for the presence of antibodies (IgG) to CHIKV, and those subjects who are positive will be excluded from participation in the study. Eligible subjects will be admitted to the clinic on the day before study drug infusion.

A subject may be rescreened for study eligibility if their originally intended sentinel or expansion group is filled and their 28-day screening window is surpassed before another group opens. The subject will be assigned a new screening number and all screening procedures will be repeated. Subjects who did not meet all enrollment criteria at their first screening will not be allowed to rescreen. Screen failures are defined as subjects who sign the consent form but who are not subsequently randomly assigned to the study intervention or entered in the study. Information on eligibility, demographics, serious AEs (SAEs), and informed consent will be collected for all screen failures.

Two safety monitoring boards, the IST, and an independent unblinded SMC, will be organized to oversee the safety of the study. The IST will oversee the safety of the entire study.

In dose level cohorts 1 through 6, the IST will approve dosing of the second and third sentinel subjects, determine advancement to dose expansion in each dose level cohort, and approve the dosing of each subject in the expansion group after review of the clinical course and safety data approximately 24 hours after each subject in the expansion group of dose level cohort 3 completes their infusion and approximately 48 hours after each subject in the expansion group of dose level cohorts 5 and 6 (and optional dose level cohort 4) completes their infusion.

In dose level cohort 7, the IST will review safety data on each sentinel subject approximately 48 hours after receipt of the first 0.3 mg/kg dose and approve receipt of the second 0.3 mg/kg dose on Day 8. The IST will review safety data approximately 48 hours after each sentinel subject receives their second 0.3 mg/kg dose to approve dosing of the subsequent sentinel subject (second and third sentinel subjects). The IST will also review safety data on all sentinel subjects approximately 48 hours after the third sentinel subject receives their second dose and approve dosing of the expansion group (5 subjects). The 5 expansion subjects will be dosed in parallel. The IST will review safety data approximately 48 hours after all 5 expansion subjects receive their first 0.3 mg/kg dose to approve dosing of the second 0.3 mg/kg dose.

The SMC will review ongoing safety data, including cumulative safety data from all study subjects, and determine if it is acceptable to escalate the dose after each dose level cohort. The SMC will be convened ad hoc in the event that study pause rules are met.

All subjects will be premedicated prior to dosing with mRNA-1944 or placebo. Premedication and the management of any suspected infusion-related reactions (IRRs) will be implemented as described below. Suspected IRRs will be assessed according to the National Cancer Institute Common Terminology Criteria for Adverse Events Version 5 (CTCAE v5, November 2017) and will include reactions assessed as related to study drug occurring during or within 24 hours after the infusion.

- On Day 1, all subjects in dose level cohorts 1 and 2, and sentinel subjects in dose level cohort 3 will be premedicated with loratadine (10 mg, oral) and ranitidine (150 mg, oral) approximately 90 minutes prior to the planned start of study drug infusion.
- On Day 1, all subjects in the expansion group of dose level cohort 3 will be premedicated with loratadine (10 mg, oral), ranitidine (150 mg, oral), and acetaminophen (650 mg, oral) approximately 90 minutes prior to the planned start of study drug infusion.
- On Day 1, all subjects in dose level cohorts 5 and 6 (and the optional dose level cohort 4) will be premedicated with dexamethasone (10 mg, IV), diphenhydramine (50 mg, oral), and famotidine (20 mg, oral) approximately 90 minutes prior to the planned start of study drug infusion.

- On Day 1 and Day 8, all subjects in dose level cohort 7 will be premedicated with diphenhydramine (50 mg, oral) and famotidine (20 mg, oral) approximately 90 minutes prior to the planned start of study drug infusion.
- Should any 1 subject develop a grade 2 IRR, their infusion will be slowed or briefly interrupted until resolution of symptoms.

Note: Management of IRRs should be based on the grade of the reaction and include slowing the rate, or temporary interruption, of the infusion (up to 4 hours). Mild to moderate IRRs (ie, CTCAE v5 grades 1 and 2 and infusion reactions that do not involve symptoms of anaphylaxis) can usually be managed with slowing, or temporary interruption, of the infusion and symptom management with the administration of additional antihistamines, antipyretics, and/or corticosteroids. Per judgment of the investigator, after all symptoms have resolved, the infusion may be resumed at a reduced rate with the administration of additional premedications as indicated.

- Because 1 subject in the 0.6 mg/kg dose level cohort (after receiving the oral premedication regimen) developed a persistent grade 2 or higher IRR, all subsequent subjects in the study who receive >0.3 mg/kg as a single dose will be administered dexamethasone (10 mg, IV), in addition to the above oral regimen, as premedication prior to the study drug infusion.
- To monitor for potential IRR, vital signs will be assessed at check-in, approximately every 15 minutes during study drug infusion, and every 15 minutes thereafter for the first hour after completion of the infusion. Continuous pulse oximetry monitoring will be implemented for the duration of the study drug infusion.
- Should any 1 subject develop a grade 3 or higher IRR (including an allergic, hypersensitivity, or anaphylactic reaction), study drug infusion will be immediately and permanently discontinued for this subject. Such an occurrence constitutes a study pause criterion and no further dosing can occur until reviewed by the SMC.

Reactions with any features of anaphylaxis (such as shortness of breath, urticaria, or angioedema) or severe IRRs (CTCAE v5 grade 3 or higher) require immediate and permanent discontinuation of the drug infusion for that subject and the convening of an SMC meeting. Suspected allergic (hypersensitivity) reactions and anaphylaxis will be assessed according to the clinical diagnostic criteria outlined by the National Institute of Allergy and Infectious Diseases and CTCAE v5.

Subjects in dose level cohorts 1 through 6 will be monitored inpatient for 48 hours following the end of infusion (EOI) on Day 1 and followed up on an outpatient basis for approximately 12 months. Subjects in dose level cohort 7, will be monitored inpatient prior to the first dose on Day 1 of the 2-dose regimen, through 48-hours following completion of the second dose on Day 8 and followed up on an outpatient basis for approximately12 months.

All subjects will return to the clinic for visits on Days 7 (±2) (dose level cohorts 1 through 6), 14 (±2), 21(±2), and 28 (±3) and Weeks 8 (±1), 12 (±2), 24 (±2), 36 (±2), 48 (±2), and 52 (±2) after the study drug infusion.

Safety assessments will include monitoring and recording of AEs, prior and concomitant medication, clinical laboratory test results, electrocardiogram (ECG) results (and cardiac enzymes when obtained per protocol), vital sign measurements, and physical examination findings.

Blood samples for the determination of serum mRNA-1944 encoding for the CHIKV24 IgG and plasma IAL concentrations will be collected for all subjects in dose level cohorts 1, 2, 4, 5, and 6 and for the sentinel subjects in dose level cohort 3, within 60 minutes before study drug infusion; at mid-infusion (0.5 hours); at EOI (1 hour [+5 minutes]); at 2, 4, 6, 8, 12, 18, 24, 36, and 48 hours post-dose; and during visits on Days 7, 14, 21 and 28 post-dose. For the 3 expansion subjects in dose level cohort 3 for whom the infusion rate is 3 hours, blood samples will be collected within 60 minutes before study drug infusion; at mid-infusion (1.5 hours); at EOI (3 hours [+5 minutes]); at 4, 6, 8, 12, 18, 24, 36, and 48 hours post-dose; and during visits on Days 7, 14, 21, and 28 post-dose. For subjects in dose level cohort 7, blood samples will be collected on Day 1 within 60 minutes before study drug infusion, at mid-infusion (0.5 hours), and at EOI (1 hour [+5 minutes]); at 2, 4, 6, 8, 12, 18, 24, 36, and 48 hours post-dose; on Day 7; again on Day 8 within 60 minutes before study drug infusion, at mid-infusion (0.5 hours), and at EOI (1 hour [+5 minutes]); at 2, 4, 6, 8, 12, 18, 24, 36, and 48 hours post-dose; and during visits on Days 14, 21, and 28 post-dose. All of these time points are with respect to the start of infusion (for the 2-, 4-, 6-, 8-, 12-, and 18-hour time points, a window of ±15 minutes is acceptable; for the 24-, 36-, and 48-hour time points, a window of ±30 minutes is acceptable).

Note: if the total infusion time is extended beyond 1 hour, the blood sample should be collected at EOI; if it overlaps with pre-specified time points, sampling should continue at the next scheduled timepoint relative to the start of infusion.

Blood samples for the determination of serum (mRNA expressed) CHIKV24 IgG concentrations will be collected for all subjects in dose level cohorts 1, 2, 4, 5, and 6 and for the sentinel subjects in dose level cohort 3 within 60 minutes before study drug infusion; at mid-infusion (0.5 hours); at EOI (1 hour [+5 minutes]); at 2, 4, 6, 8, 12, 18, 24, 36, and 48 hours post-dose; during visits on Days 7, 14, 21, and 28 post-dose; and at Weeks 8, 12, 24, 36, 48, and 52 post-dose. For the 3 expansion subjects in dose level cohort 3, for whom the infusion rate is 3 hours blood samples will be collected within 60 minutes before study drug infusion; at mid-infusion (1.5 hours); at EOI (3 hours [+5 minutes]); at 4, 6, 8, 12, 18, 24, 36, and 48 hours post-dose; during visits on Days 7, 14, 21, and 28 post-dose; and at Weeks 8, 12, 24, 36, 48, and 52 post-dose. For subjects in dose level cohort 7, blood samples will be collected on Day 1 within 60 minutes before study drug infusion, at mid-infusion (0.5 hours), and at EOI (1 hour [+5 minutes]); at 2, 4, 6, 8, 12, 18, 24, 36, and 48 hours post-dose; on Day 7; and again on Day 8 within 60 minutes before study drug infusion; at mid-infusion (0.5 hours), and at EOI

(1 hour [+5 minutes]); at 2, 4, 6, 8, 12, 18, 24, 36, and 48 hours post-dose; during visits on Days 14, 21 and 28 post-dose; and at Weeks 8, 12, 24, 36, 48, and 52 post-dose. All of these time points are with respect to the start of infusion (for the 2-, 4-, 6-, 8-, 12-, and 18-hour time points, a window of ±15 minutes is acceptable; for the 24-, 36-, and 48- hour time points, a window of ±30 minutes is acceptable).

Blood samples for the determination of anti-PEG antibodies will be collected for dose level cohorts 1 through 6 within 60 minutes before study drug infusion; during visits on Days 7, 14, 21, and 28 post-dose; and Weeks 8, 12, 24, 36, 48, and 52 post-dose. For dose level cohort 7, these samples will be collected within 60 minutes before study drug infusion on Day 1; on Day 7; within 60 minutes before study drug infusion on Day 8; during visits on Days 14, 21, and 28 post-dose; and at Weeks 8, 12, 24, 36, 48, and 52 post-dose.

Blood samples for the determination of anti-CHIKV24 IgG antibodies will be collected for dose level cohorts 1 through 6 within 60 minutes before study drug infusion; during visits on Days 7, 14, 21, and 28 post-dose; and at Weeks 8, 12, 24, 36, 48, and 52 post-dose. For dose level cohort 7, these samples will be collected within 60 minutes before study drug infusion on Day 1; on Day 7; within 60 minutes before study drug infusion on Day 8; during visits on Days 14, 21, and 28 post-dose; and at Weeks 8, 12, 24, 36, 48, and 52 post-dose.

Blood samples for the determination of in vitro serum neutralizing antibody titer against a clinically relevant strain of CHIKV will be collected for dose level cohorts 1 through 6 within 60 minutes before study drug infusion; and at 12, 24, and 48 hours post-dose. For dose level cohort 7, these samples will be collected within 60 minutes before study drug infusion on Day 1; at 12, 24, and 48 hours post-dose; within 60 minutes before study drug infusion on Day 8; and at 12, 24, and 48 hours post-dose.

A blood sample for DNA isolation in order to determine genotyping and to determine the concentration levels of Apo E will be collected within 60 minutes before study drug infusion on Day 1 from subjects who have consented to participate in the genetic analysis component of the study.

Blood samples for the assessment of complement factors C5b-9 will be collected pre-dose (within 60 minutes before study drug infusion), and at 90 (±5) minutes (from the start of infusion); at 2, 6, 12, and 24 (±15 minutes) hours post-dose (completion of the infusion); and at the time of discharge (within 60 minutes) from the inpatient unit. If the subject experiences an infusion reaction, a blood sample for analysis of complement factors should be obtained within 1 hour of the start of the reaction.

Blood samples for the assessment of CRP will be collected pre-dose (within 60 minutes before study drug infusion) and 6- and 24-hours post-dose (completion of infusion), and at the time of discharge (within 60 minutes) from the inpatient unit. Blood samples for IL-6 and IP-10 will be collected pre-dose (within 60 minutes before study drug infusion), and at 90 minutes (±5) (from the start of infusion); at 2, 6, 12, and 24 (±15 minutes) hours post-dose (completion of

the infusion); and at the time of discharge (within 60 minutes) from the inpatient unit. If the subject experiences an infusion reaction, a blood sample for analysis of CRP and cytokine factors should be obtained within 1 hour of the start of the reaction.

Sentinel Dosing Strategy

Eight subjects are planned to be enrolled at each dose level cohort. For each dose level cohort, a sentinel dosing strategy will be employed.

For dose level cohorts 1 through 6, a safety group of 3 subjects will be enrolled; all will receive mRNA-1944, with a staggered minimum 7-day interval between each subject prior to treating the remaining subjects within a dose cohort. Each sentinel subject will be followed up for 7 days after the study drug infusion (the first 48 hours will be inpatient) with an IST review of the safety results through 7 days following the study drug infusion prior to the enrollment of each subsequent sentinel subject. The IST will then review all safety data, including cumulative safety data, for all sentinel subjects prior to randomly assigning the remainder of the dose level cohort (5 subjects, 3 active:2 placebo).

In dose level cohort 7, a safety group of 3 subjects will be enrolled; all will receive two doses of 0.3 mg/kg, one infusion on Day 1 and another subsequent infusion on Day 8. Each sentinel subject will remain inhouse from Day –1 to Day 10 (48 hours after the second 0.3 mg/kg infusion). The IST will review the safety results approximately 48 hours after the first 0.3 mg/kg dose to approve receipt of the second 0.3 mg/kg dose. The IST will also review data 48 hours after each sentinel subject receives their second dose to approve dosing of the subsequent sentinel subject (second and third sentinel subjects). The IST will also review all safety data on all sentinel subjects approximately 48 hours after the third sentinel subject receives their second dose and approve dosing of the expansion subjects (5 subjects, 3 active:2 placebo). The 5 expansion subjects will be dosed in parallel.

Internal Safety Team and Dose Expansion

The IST will oversee the safety of the study and will evaluate ongoing safety data throughout the study to ensure adherence to the protocol. For dose level cohorts 1 through 6, the IST will monitor safety data to approve dosing of the second and third sentinel subjects in each dose level cohort, to determine advancement to dose expansion in each dose level cohort, and to approve the dosing of each subject in the expansion group after review of the clinical course and safety data approximately 24 hours after each subject in the expansion group of dose level cohort 3 completes their infusion and approximately 48 hours after each subject in the expansion group of dose level cohorts 5 and 6 (and optional dose level cohort 4) completed their infusion. The IST will review the cumulative safety data weekly during the active enrollment of subjects into dose level cohorts in addition to reviewing prior to each dose level expansion. After review by the IST of the safety data through 7 days after the study drug

infusion of all sentinel safety lead-ins for each cohort, approval may be given to proceed with enrollment of the remainder of that dose level cohort.

For dose level cohort 7, the IST will review safety data on each sentinel subject approximately 48 hours after receipt of the first 0.3 mg/kg dose and approve receipt of the second 0.3 mg/kg dose on Day 8. The IST will review safety data approximately 48 hours after each sentinel subject receives their second 0.3 mg/kg dose to approve dosing of the subsequent sentinel subject (second and third sentinel subjects). The IST will also review safety data on all sentinel subjects approximately 48 hours after the third sentinel subject receives their second dose and approve dosing of the expansion group (5 subjects). The 5 expansion subjects will be dosed in parallel. The IST will review safety data approximately 48 hours after all 5 expansion subjects receive their first 0.3 mg/kg dose to approve dosing of the second 0.3 mg/kg dose. Cohort 7 may run in parallel with dose level cohort 5, regardless of whether dosing has begun in cohort 6.

If the IST determines that a pre-established pause rule is met or identifies any other potential safety concern, the independent unblinded SMC will be notified. In such cases, study enrollment and all further dosing will be paused, the SMC will be convened to determine whether treatment may be resumed, whether any revisions to the protocol are warranted, or if the study should be stopped.

Safety Monitoring Committee and Dose Escalation

Seven dose levels of mRNA-1944 are planned to be investigated in this study (one optional). The SMC will review the unblinded safety data of the entire currently dosed cohort through 7 days after the study drug infusion, and cumulative safety data from all study subjects prior to escalation to the next dose level. Dose escalation to higher dose level cohorts may continue until a study pause rule is reached or upon adequate pharmacological characterization of the mRNA-1944 encoded antibody is achieved. The SMC may also be convened ad hoc to support the conduct of the study. For all enrollment pauses in the study that are triggered by the pause rules, the SMC will be convened to determine whether treatment may be resumed, whether any revisions to the protocol are warranted, or if the study should be stopped. Optional dose level cohorts for intermediate dose levels may be added for de-escalation of the dose after review of cumulative data by the SMC.

Pause Rules

The occurrence of any of the following criteria results in a pause and no further dosing can occur until reviewed by the SMC:

- Any SAE, irrespective of assessed relatedness to study drug*.
- Two or more subjects experience a grade 3 or higher AE, including a laboratory abnormality, of the same nature.

- Any subject develops a grade 3 or higher IRR (including an allergic, hypersensitivity, or anaphylactic reaction).
- Any clinical event that, in the opinion of the SMC, is a contraindication to further dosing of additional subjects.

*Serious AEs that are the result of external causes and clearly not related to study drug, as assessed by both the investigator and the Sponsor, eg, subject involved in an automobile accident or a sports-related injury, will not result in a pause.

For any enrollment pauses to the study that are triggered by the pause rules, the SMC will be convened to determine whether treatment can be resumed, whether any revisions to the protocol are warranted, or if the study should be stopped.

STUDY POPULATION:

Inclusion Criteria:

Each subject must meet all of the following criteria to be enrolled in this study:

1. The subject is male or female ≥ 18 and ≤ 50 years of age.
2. The subject has a weight of 50 to ≤ 90 kg, inclusive and a maximum body mass index ≤ 33 kg/m^2.
3. The subject is considered by the investigator to be in good general health as determined by medical history, clinical laboratory assessments, ECG results, vital sign measurements, and physical examination findings at screening.
4. Female subjects of nonchildbearing potential may be enrolled in the study. Nonchildbearing potential is defined as bilateral tubal ligation >1 year prior to screening, bilateral oophorectomy, or hysterectomy or menopause. Follicle-stimulating hormone level may be measured, at the discretion of the investigator, to confirm menopausal status.
5. Female subjects of childbearing potential may be enrolled in the study, if the subject meets the following criteria: 1) has a negative pregnancy test at screening and at check-in, 2) has practiced adequate contraception or abstained from all activities that could lead to pregnancy for 30 days prior to study drug infusion, and 3) has agreed to continue adequate contraception through 36 weeks after study drug infusion.
6. Male subjects must agree to consistently use adequate contraception and refrain from sperm donation through 36 weeks after study drug infusion.
7. The subject understands and agrees to comply with the study procedures and provides written informed consent before any study procedures are performed.
8. The subject agrees to stay in contact with the study site for the duration of the study and to provide updated contact information, as necessary for the duration of the study.

Exclusion Criteria:

Subjects meeting any of the following criteria will be excluded from the study:

1. Has an acute or chronic clinically significant disease, as determined by physical examination or laboratory screening tests. Asymptomatic conditions or findings (eg, mild hypertension, dyslipidemia) are not exclusionary if they are being appropriately managed, are clinically stable, and are unlikely to progress within the study period in the opinion of the investigator.
2. If female and of childbearing potential, has a positive serum pregnancy test at screening or on Day –1.
3. If female and of childbearing potential, is pregnant or lactating, has not adhered to an adequate contraception method for at least 30 days before study entry, or is unwilling to use adequate contraception for at least 36 weeks after the study drug infusion.
4. Has elevated liver function tests, defined as aspartate aminotransferase, alanine aminotransferase, or alkaline phosphatase, as well as elevated creatinine, or decreased platelets, with a toxicity score of grade 1 or higher at screening. Retesting of these parameters is not allowed.
5. Has safety laboratory test results (hematology, chemistry, and coagulation) of grade 2 or higher at screening and baseline (Section 10.4). The inclusion of subjects with non-clinically significant grade 1 laboratory abnormalities (other than those noted in Exclusion Criterion 4) is allowed based on the investigator's discretion.
6. Has a positive screening test for the presence of anti-CHIKV IgG.
7. Has participated in another investigational study involving any investigational product (ie, study drug, biologic, device) within 60 days or 5 half-lives, whichever is longer, before the planned date of study drug infusion (Day 1).
8. Has received any live attenuated or inactive vaccines within 4 weeks prior to check-in or plans to receive any vaccine during the study; a seasonal influenza vaccine is permissible.
9. Has received (at any time) a vaccine for CHIKV, dengue, yellow fever, tick-borne encephalitis, or Japanese encephalitis.
10. Has previously participated in an investigational study involving lipid nanoparticles.
11. Has a known or suspected immune-mediated disease or immunosuppressive condition (including lymphoproliferative disorders) as determined by medical history and/or physical examination (Section 7.4.1.3).
12. Has a neurologic disorder (eg, history of seizures, Guillain-Barre syndrome, dementia, vasculitis, or any known congenital or acquired disorder).
13. Has a history of idiopathic urticaria.
14. Has any bleeding disorder that is considered a contraindication to study drug infusion or blood collection.

15. Has any medical, psychiatric, or occupational condition that, in the opinion of the investigator, might pose an additional risk to the subject if they participate in the study or might interfere with the evaluation of mRNA-1944 or the interpretation of study results.
16. Has received immunoglobulins, a monoclonal antibody or any blood products within the 6 months preceding administration of the study drug or plans to receive such products at any time during the study.
17. Has received heparin treatment within the last 30 days prior to study drug infusion or plans to receive any heparin treatment during the study.
18. Is currently receiving antipyretic or analgesic medication on a daily or every other day basis (a daily dose of ≤100 mg of aspirin given under the guidance of a physician is not a contraindication to enrollment).
19. Has any acute disease or fever at the time of screening. Fever is defined as body temperature ≥ 38.0°C/100.4°F by the oral, axillary, or tympanic route. Subjects with an acute disease or fever at the time of screening may be re-evaluated at a later date. Subjects with a minor illness without fever can be enrolled at the discretion of the investigator (defined as the presence of a moderate or severe illness with or without fever, or an oral temperature > 38.0°C on the planned day of infusion). In such cases, subjects may be re-evaluated during the screening period for resolution of the illness to allow at least 3 days of wellness prior to the planned study drug infusion.
20. Has a positive urine drug screen for any of the following nonprescription drugs of abuse at screening or at check-in: alcohol, opiates, cocaine, phencyclidine, amphetamines, benzodiazepines, or methadone. Positive drug screens for amphetamines, benzodiazepines, or opiates will not be exclusionary if prescribed concomitant medications can justify the result.
21. Has known allergies to any components or excipients of the study drug, as detailed in Section 6.2.
22. Has any condition that, in the opinion of the investigator, would pose a health risk to the subject if they enrolled or could interfere with evaluation of the study drug or the interpretation of study results (including neurologic or psychiatric conditions deemed likely to impair the quality of safety reporting).
23. Has a positive test result for hepatitis B surface antigen, hepatitis C virus antibody, or human immunodeficiency virus type 1 or 2 antibodies.
24. Has a history of active cancer (malignancy) in the last 3 years. An exception is a subject with adequately treated nonmelanomatous skin carcinoma, who may participate in the study.
25. Has donated ≥ 450 mL blood or blood products within 30 days of study drug infusion.
26. Has any grade 2 or higher vital sign measurement (systolic or diastolic blood pressure, heart rate, or respiratory rate) at screening after 2 measurements.

27. Has a prolonged Fridericia-corrected QT interval at screening or check-in, defined as > 450 millisecond for males and > 460 millisecond for females, confirmed by repeat.
28. Is an employee or first-degree relative of Sponsor, contract research organization, or study site personnel.

STUDY TREATMENTS:

Dose level cohorts 1, 2, 4, 5, and 6 and the sentinel subjects of dose level cohort 3: A single *IV* infusion of 100 mL of mRNA-1944 or placebo administered over 1 hours on Day 1.

For the 3 expansion subjects in dose level cohort 3: A single *IV* infusion of 100 mL of mRNA-1944 or placebo administered over 3 hours on Day 1.

Dose level cohort 7: Two single *IV* infusions of 100 mL of mRNA-1944 or placebo administered over 1 hour on Day 1 and Day 8.

STUDY PROCEDURES:

Pharmacokinetic Assessments and Endpoints:

In all dose level cohorts, blood samples will be collected at prespecified times outlined in the schedule of events (SOE) (Section 1) to determine the serum concentrations of mRNA encoding for CHIKV24 IgG and the plasma concentrations of IAL.

The following PK parameters, where possible, will be calculated as endpoints for serum mRNA encoding for the CHIKV24 IgG and plasma IAL using the actual sampling times relative to the start of infusion rather than the scheduled sampling times: area under the concentration versus time curve (AUC) from time 0 to the last measurable concentration (AUC_{last}), AUC from time 0 extrapolated to infinity (AUC_{inf}), maximum observed serum concentration (C_{max}), time to maximum observed serum concentration (t_{max}), terminal elimination half-life ($t_{1/2}$), apparent clearance (CL), and volume of distribution at steady state (V_{ss}).

Pharmacodynamic Assessments and Endpoints:

In all dose level cohorts, blood samples will be collected at the prespecified times outlined in the SOE (Section 1) to determine the serum concentrations of CHIKV24 IgG.

The following baseline corrected serum PD parameters will be calculated as endpoints for CHIKV24 IgG concentration using the actual sampling times relative to the start of infusion: maximum observed effect (E_{max}), time to the maximum observed effect (TE_{max}), $t_{1/2}$, area under the effect curve (AUEC) from time 0 to the last measurable concentration ($AUEC_{last}$), and AUEC from time 0 extrapolated to infinity ($AUEC_{inf}$).

Exploratory Assessments and Endpoints:

In all dose level cohorts, blood samples will be collected at the prespecified times outlined in the SOE (Section 1) to evaluate for the formation of anti-PEG, anti-CHIKV24 IgG titers, the

assessment of in vitro serum neutralizing antibody titer, and complement and acute phase reactants.

A blood sample for DNA isolation in order to determine genotyping of Apo E will be collected from subjects who have consented to participate in the genetic analysis component of the study. Participation is optional. Subjects who do not wish to participate in the genetic research may still participate in the study.

Safety Assessments and Endpoints:

Safety and tolerability will be assessed by the following endpoints: monitoring and recording of AEs (including SAEs, IRRs, and AEs of special interest [AESI]), prior and concomitant medication, clinical laboratory test results (hematology, coagulation, serum chemistry including liver enzymes, and urinalysis), vital sign measurements (systolic and diastolic blood pressure, heart rate, respiratory rate, and body temperature), ECG results (and cardiac enzymes when obtained per protocol), and physical examination findings.

STATISTICAL ANALYSIS PLANS:

Sample Size:

Approximately 56 subjects (8 per cohort) are planned to be enrolled; 6 subjects will receive mRNA-1944 and 2 subjects will receive placebo in each dose level cohort. Among the 6 subjects receiving mRNA-1944, 3 are the sentinel subjects. The remaining 5 subjects within each dose level cohort will be randomly assigned in a 3:2 ratio to receive mRNA-1944 or placebo. Formal sample size calculations were not performed, and no formal hypotheses are being tested.

Analysis Sets:

The Safety Population will include all subjects who enrolled and received any dose infusion of mRNA-1944 or placebo. The Safety Population will be used in safety and exploratory analyses.

The PK analysis dataset will include subjects in the Safety Population who have evaluable mRNA encoding for CHIKV24 IgG and IAL concentrations and do not have any major protocol deviations impacting the PK assessments.

The PD analysis dataset will include subjects in the Safety Population who have evaluable CHIKV24 IgG concentrations and do not have any major protocol deviations impacting the PD assessments.

Pharmacokinetic Analyses:

Serum mRNA encoding for CHIKV24 IgG and plasma IAL concentrations will be listed, presented graphically, and summarized descriptively. Pharmacokinetic parameters (C_{max}, t_{max}, AUC_{last}, AUC_{inf}, $t_{1/2}$, CL, and V_{ss}) will be listed and summarized by dose level and treatment.

Furthermore, the relationship between dose and systemic exposure to mRNA- encoding for CHIKV24 IgG and IAL will be assessed.

Pharmacodynamic Analyses:

CHIKV24 IgG concentrations will be listed, presented graphically, and summarized descriptively. Pharmacodynamic parameters (E_{max}, TE_{max}, $t_{1/2}$, $AUEC_{last}$ and $AUEC_{inf}$) derived from sample concentrations will be listed and summarized by dose level and treatment. Furthermore, the relationship between mRNA-1944 dose exposure to the extent of CHIKV24 IgG levels will be assessed.

Additional analyses may be conducted to evaluate the effect of subject characteristics on the PK and PD of mRNA and IAL, as appropriate.

Safety Analyses:

Adverse events will be coded by preferred term and system organ class using the latest version of the Medical Dictionary for Regulatory Activities (MedDRA). All AE data will be presented in a data listing. Treatment-emergent AEs will be summarized by treatment group and overall, as well as by severity (toxicity grading) and relationship to study drug. Serious AEs, IRRs, AESIs AEs leading to study withdrawal or dose modification, and deaths will be presented in the data listings and summarized by treatment, dose, and overall. Data for subjects receiving placebo will be pooled across all dose levels.

Actual values and changes from baseline for clinical laboratory test results, ECG results (and cardiac enzymes when obtained per protocol), and vital sign measurements will be summarized by dose level and treatment at each time point using descriptive statistics (number of subjects, mean, SD, median, minimum, and maximum). Clinical laboratory test results and vital sign measurements will be assessed by toxicity grading. All data will be presented in data listings.

1. SCHEDULE OF EVENTS

1.1 Dose Level Cohorts 1 through 6

Phase	Screening	Check-in	Study Day							Study Week					
Procedure[a] Day/Week	−28 to −1	−1	1	2	3	7	14	21	28	8	12	24	36	48	52 (EOS/ ET)
Window						±2 Days	±2 Days	±2 Days	±3 Days	±1 Week	±2 Weeks	±2 Weeks	±2 Weeks	±2 Weeks	±2 Weeks
Informed consent	X														
Inclusion/exclusion criteria	X	X													
Medical history	X														
Physical examination[b]	X	X		X	X	X	X								X
Vital sign measurements[c]	X	X	X[d]	X	X	X	X			X			X		X
12-Lead electrocardiogram	X	X	X[e]	X	X										X
Serology[f]	X														
Anti-CHIKV IgG screening	X[g]														
Clinical laboratory testing[h]	X	X		X	X	X	X		X	X			X		X
Urine drug screen[i]	X	X													
Serum pregnancy test (all female subjects of childbearing potential)	X	X													
Serum FSH[j]	X														
Admission to the clinic		X													
Randomization[k]			X												
Pretreatment [l]			X												
Study drug administration			X												
Infusion site assessments[m]			X	X	X	X									

Phase	Screening	Check-in	Study Day							Study Week					
Procedure[(a)] Day/Week	−28 to −1	−1	1	2	3	7	14	21	28	8	12	24	36	48	52 (EOS/ ET)
Window						±2 Days	±2 Days	±2 Days	±3 Days	±1 Week	±2 Weeks	±2 Weeks	±2 Weeks	±2 Weeks	±2 Weeks
Discharge from clinic					X										
Outpatient visit						X	X	X	X	X	X	X	X	X	X
Blood sample for CHIKV24 IgG mRNA level[(n)]			X	X	X	X	X	X	X						
Blood sample for IAL level[(n)]			X	X	X	X	X	X	X						
Blood sample for CHIKV24 IgG quantification[(o)]			X	X	X	X	X	X	X	X	X	X	X	X	X
Blood sample for anti-PEG[(p)]			X			X	X	X	X	X	X	X	X	X	X
Blood sample for anti-CHIKV24 IgG[(q)]	X[(g)]		X			X	X	X	X	X	X	X	X	X	X
Blood sample for neutralizing antibody titer[(r)]			X	X	X										
Blood sample for Apolipoprotein E and lipid panel			X												
Prior and concomitant medication[(s)]	X	X	X	X	X	X	X	X	X	X	X	X	X	X	X
Adverse events assessments[(t)]	X	X	X	X	X	X	X	X	X	X	X	X	X	X	X
Complement (C5b-9)[(u)]			X	X	X										
Cytokines and CRP (IP-10, IL-6)[(v)]			X	X	X										

Abbreviations: AEs, adverse events; AESI, adverse event of special interest; CHIKV, chikungunya virus; CRP, C-reactive protein; EOS, end of study; EOI, end of infusion; ET, early termination; FSH, follicle-stimulating hormone; IgG, immunoglobulin G; IL-6, interleukin-6; IP-10, interferon-inducible protein-10; mRNA, messenger RNA; PEG, polyethylene glycol; PK, pharmacokinetic; SAE, serious adverse event.

[a] When procedures overlap or occur at the same time point, all blood collections should follow vital sign measurements and PK sampling and be timed to occur last and to be as close as possible to the nominal time point. Activities listed at the EOI, will be collected after the completion of the study drug infusion.

[b] Full physical examination at screening and at check-in; symptom directed (targeted) physical examination at all other scheduled time points. Interim physical examinations will be performed at the discretion of the investigator, if necessary. Height will be measured at screening only. Weight will be measured at screening as well at check-in, for dose calculation.

[c] Vital sign measurements (systolic and diastolic blood pressures, heart rate, respiratory rate, and body temperature) will be collected after the subject has been resting in a semi-recumbent position for at least 5 minutes.

[d] Vital signs on Day 1 will be measured within 60 minutes before study drug infusion and every 15 minutes during study drug infusion and for the first hour following the EOI. Continuous pulse oximetry monitoring will be implemented for the duration of the infusion.

[e] 12-Lead electrocardiogram will be collected on Day 1 at 1, 4, and 5 hours after the start of study drug infusion. If a clinically significant change from screening is noted on the electrocardiogram, blood samples for the assessments of cardiac enzymes (troponin I and creatine kinase-MB) will be drawn within 1 hour and at 6 and 24 hours after the noted abnormal electrocardiogram.

[f] Serology testing will include hepatitis B surface antigen, hepatitis C virus antibody, and human immunodeficiency virus type 1 and 2 antibodies.

[g] Subjects with a positive result will be excluded from participation in the study.

[h] Hematology, serum chemistry, coagulation, and urinalysis assessments. Urinalysis will be collected at screening, Day 2, and Day 7.

[i] The drug screen will include alcohol, opiates, cocaine, phencyclidine, amphetamines, benzodiazepines, and methadone.

[j] To confirm postmenopausal status, as needed.

[k] Within each dose level cohort, 6 subjects will receive mRNA-1944 and 2 subjects will receive placebo. For each dose level cohort, a sentinel group of 3 subjects will be enrolled, and all will receive mRNA-1944, with a staggered minimum 7-day interval between each subject prior to treating the remaining subjects within a dose level cohort. *PPD* will generate the randomization schedule. The 5 remaining subjects in each dose level cohort will be assigned a randomization number and randomly assigned to mRNA-1944 (0.1, 0.3, 0.45, 0.6, and 1.0 mg/kg) or placebo in a 3:2 ratio (active:placebo) for an overall ratio of 3:1 according to the randomization schedule. Randomization numbers will be assigned in a sequential, ascending fashion and sealed in an envelope.

[l] Pretreatment with dexamethasone (10 mg, IV [dose level cohorts 5 and 6 and optional dose level cohort 4), diphenhydramine(50 mg, oral), and famotidine (20 mg, oral) will occur approximately 90 minutes prior to the planned start of study drug infusion.

[m] Infusion site assessments will be performed for all subjects within 15 minutes prior to initiation of the study drug infusion; at 1, 3, 24, and 48 hours after the start of the study drug infusion; and on Day 7.

[n] Blood samples for the determination of serum mRNA-1944 encoding for CHIKV24 IgG and plasma IAL concentrations will be collected for dose level cohorts 1, 2, 4, 5, and 6 and for the sentinel subjects in dose level cohort 3 within 60 minutes before study drug infusion; at mid-infusion (0.5 hours); at EOI (1 hour [+5 minutes]); at 2, 4, 6, 8, 12, 18, 24, 36, and 48 hours post-dose; and during visits on Days 7, 14, 21 and 28 post-dose. For the 3 expansion subjects in dose level cohort 3, for whom the infusion rate was 3 hours, blood samples will be collected within 60 minutes before study drug infusion; at mid-infusion (1.5 hours); at EOI (3 hours [+5 minutes]); at 4, 6, 8, 12, 18, 24, 36, and 48 hours post-dose; and during visits on Days 7, 14, 21 and 28 post-dose. All sampling time points are relative to the start of study drug infusion.

[o] Blood samples for the determination of serum (mRNA expressed) CHIKV24 IgG concentrations will be collected for dose level cohorts 1, 2, 4, 5, and 6 and for the sentinel subject in dose level cohort 3 within 60 minutes before study drug infusion; at mid-infusion (0.5 hours); at EOI (1 hour [+5 minutes]); t 2, 4, 6, 8, 12, 18, 24, 36, and 48 hours post-dose; during visits on Days 7, 14, 21, and 28 post-dose; and Weeks 8, 12, 24, 36, 48, and 52 post-dose. For the 3 expansion subject in dose level cohort 3 blood samples will be collected within 60 minutes before study drug infusion; at mid-infusion (1.5 hours); at EOI (3 hours [+5 minutes]); at 4, 6, 8, 12, 18, 24, 36, and 48 hours post-dose; during visits on Days 7, 14, 21, and 28 post-dose; and Weeks 8, 12, 24, 36, 48, and 52 post-dose. Time points are relative to the start of study drug infusion.

[p] Blood samples for the determination of anti-PEG antibodies will be collected within 60 minutes before study drug infusion; during visits on Days 7, 14, 21, and 28 post-dose; and Weeks 8, 12, 24, 36, 48, and 52 post-dose. Time points are relative to the start of study drug infusion.

[q] Blood samples for the determination of anti-CHIKV24 IgG antibodies will be collected within 60 minutes before study drug infusion; during visits on Days 7, 14, 21, and 28 post-dose; and Weeks 8, 12, 24, 36, 48, and 52 post-dose. Time points are relative to the start of study drug infusion.

[r] Blood samples for determination of in vitro serum neutralizing antibody titer against a clinically relevant strain of CHIKV will be collected within 60 minutes before study drug infusion and at 12, 24, and 48 hours post-dose. Timepoints are relative to the start of study drug infusion.

[s] Prior medications taken by the subject within the 30 days before providing informed consent will be collected. Concomitant medications include all medications taken by the subject from the time of signing the informed consent and through the Week 52 visit.

[t] Adverse events will be assessed from the time of informed consent until EOS/ET and should be followed until they resolve, stabilize, or judged by the investigator to be not clinically significant. All AEs will be recorded through 28 days after study drug infusion, all medically attended AEs will be recorded for 3 months after study drug infusion, and all SAEs and AESIs (Section 7.4.1.3) will be recorded through Week 52.

[u] Blood samples for the assessment of complement factors C5b-9 will be collected pre-dose (within 60 minutes before study drug infusion), and at 90 (±5) minutes (from the start of infusion); at 2, 6, 12, and 24 (±15 minutes) hours post-dose (completion of the infusion); and at the time of discharge (within 60 minutes) from the inpatient unit. If the subject experiences an infusion reaction, a blood sample for analysis of complement factors should be obtained within 1 hour of the start of the reaction.

[v] Blood samples for the assessment of CRP will be collected pre-dose (within 60 minutes before study drug infusion) and 6- and 24-hours post-dose (completion of infusion), and at the time of discharge (within 60 minutes) from the inpatient unit. Blood samples for IL-6 and IP-10 will be collected pre-dose (within 60 minutes before study drug infusion), and at 90 minutes (±5) (from the start of infusion); at 2, 6, 12, and 24 (±15 minutes) hours post-dose (completion of the infusion); and at the time of discharge (within 60 minutes) from the inpatient unit. If the subject experiences an infusion reaction, a blood sample for analysis of CRP and cytokine factors should be obtained within 1 hour of the start of the reaction.

1.2 Dose Level Cohort 7 (0.3 mg/kg × 2 Doses, Administered at a 7-day Interval)

**Phase**	**Screening**	**Check-in**	**Study Day**										**Study Week**					
**Procedure[(0)]** **Day/Week**	**−28 to −1**	**−1**	**1**	**2**	**3**	**7**	**8**	**9**	**10**	**14**	**21**	**28**	**8**	**12**	**24**	**36**	**48**	**52 (EOS/ ET)**
**Window**										**±2 Days**	**±2 Days**	**±3 Days**	**±1 Week**	**±2 Weeks**	**±2 Weeks**	**±2 Weeks**	**±2 Weeks**	**±2 Weeks**
Informed consent	X																	
Inclusion/exclusion criteria	X	X																
Medical history	X																	
Physical examination[(b)]	X	X		X	X	X		X	X	X								X
Vital sign measurements[(c)]	X	X	X[(d)]	X	X	X	X[(d)]	X	X	X			X			X		X
12-Lead electrocardiogram	X	X	X[(e)]	X	X	X	X[(e)]	X	X	X								X
Serology[(f)]	X																	
Anti-CHIKV IgG screening	X[(g)]																	
Clinical laboratory testing[(h)]	X	X		X	X	X		X	X	X		X	X			X		X
Urine drug screen[(i)]	X	X																
Serum pregnancy test (all female subjects of childbearing potential)	X	X																
Serum FSH[(j)]	X																	
Admission to the clinic		X																
Randomization[(k)]			X															
Pretreatment [(l)]			X				X											
Study drug administration			X				X											
Infusion site assessments[(k)]			X	X	X	X	X	X	X	X								
Discharge from clinic									X									
Outpatient visit										X	X	X	X	X	X	X	X	X

Deleted:

Deleted:

Phase		Screening	Check-in	Study Day										Study Week					
Procedure[(0)]	Day/Week	−28 to −1	−1	1	2	3	7	8	9	10	14	21	28	8	12	24	36	48	52 (EOS/ ET)
	Window										±2 Days	±2 Days	±3 Days	±1 Week	±2 Weeks	±2 Weeks	±2 Weeks	±2 Weeks	±2 Weeks
Blood sample for CHIKV24 IgG mRNA level[(n)]				X	X	X	X	X	X	X	X	X	X						
Blood sample for IAL level[(n)]				X	X	X	X	X	X	X	X	X	X						
Blood sample for CHIKV24 IgG quantification[(o)]				X	X	X	X	X	X	X	X	X	X	X	X	X	X	X	X
Blood sample for anti-PEG[(p)]				X			X	X			X	X	X	X	X	X	X	X	X
Blood sample for anti-CHIKV24 IgG[(q)]		X[(g)]		X			X	X			X	X	X	X	X	X	X	X	X
Blood sample for neutralizing antibody titer[(r)]				X	X	X		X	X	X									
Blood sample for apolipoprotein E and lipid panel				X															
Prior and concomitant medication[(s)]		X	X	X	X	X	X	X	X	X	X	X	X	X	X	X	X	X	X
Adverse events assessments[(t)]		X	X	X	X	X	X	X	X	X	X	X	X	X	X	X	X	X	X
Complement (C5b-9)[(u)]				X	X	X		X	X	X									
Cytokines and CRP (IP-10, IL-6)[(v)]				X	X	X		X	X	X									

Deleted:

Abbreviations: AEs, adverse events; AESI, adverse event of special interest; CHIKV, chikungunya virus; CRP, C-reactive protein; EOS, end of study; EOI, end of infusion; ET, early termination; FSH, follicle-stimulating hormone; IgG, immunoglobulin G; IL-6, interleukin-6; IP-10, interferon-inducible protein-10; mRNA, messenger RNA; PEG, polyethylene glycol; PK, pharmacokinetic; SAE, serious adverse event.

[a] When procedures overlap or occur at the same time point, all blood collections should follow vital sign measurements and PK sampling and be timed to occur last and to be as close as possible to the nominal time point. Activities listed at the EOI, will be collected after the completion of the study drug infusion.

[b] Full physical examination at screening and at check-in; symptom directed (targeted) physical examination at all other scheduled time points. Interim physical examinations will be performed at the discretion of the investigator, if necessary. Height will be measured at screening only. Weight will be measured at screening as well as at check-in, for dose calculation.

[c] Vital sign measurements (systolic and diastolic blood pressures, heart rate, respiratory rate, and body temperature) will be collected after the subject has been resting in a semi-recumbent position for at least 5 minutes.

[d] Vital signs on Day 1 and Day 8 will be measured within 60 minutes before study drug infusion and every 15 minutes during study drug infusion and for the first hour following the EOI. Continuous pulse oximetry monitoring will be implemented for the duration of the infusion.

[e] 12-Lead electrocardiogram will be collected on Days 1, 2, 3, 7, 8, 9, 10, and 14at 1, 4, and 5 hours after the start of study drug infusion. If a clinically significant change from screening is noted on the electrocardiogram, blood samples for the assessments of cardiac enzymes (troponin I and creatine kinase-MB) will be drawn within 1 hour and at 6 and 24 hours after the noted abnormal electrocardiogram.

[f] Serology testing will include hepatitis B surface antigen, hepatitis C virus antibody, and human immunodeficiency virus type 1 and 2 antibodies.

[g] Subjects with a positive result will be excluded from participation in the study.

[h] Hematology, serum chemistry, coagulation, and urinalysis assessments. Urinalysis will be collected at screening, and Days 2, 7, 9, and 14.

[i] The drug screen will include alcohol, opiates, cocaine, phencyclidine, amphetamines, benzodiazepines, and methadone.

[j] To confirm postmenopausal status, as needed.

[k] Six subjects will receive mRNA-1944 and 2 subjects will receive placebo for an overall ratio of 3:1 (active:placebo) according to the randomization schedule. Randomization numbers will be assigned in a sequential, ascending fashion and sealed in an envelope.

[l] Pretreatment with dexamethasone (10 mg, IV [dose level cohorts 5 and 6 and optional dose level cohort 4), diphenhydramine(50 mg, oral), and famotidine (20 mg, oral) will occur approximately 90 minutes prior to the planned start of study drug infusion.

[m] Infusion site assessments will be performed for all subjects on Day 1 and Day 8 within 15 minutes prior to initiation of the study drug infusion and at 1, 3, 24, and 48 hours after the start of the study drug infusion; and on Days 7 and 14.

[n] Blood samples for the determination of serum mRNA-1944 encoding for CHIKV24 IgG and plasma IAL concentrations will be collected on Day 1 within 60 minutes before study drug infusion, at mid-infusion (0.5 hours), and at EOI (1 hour [+5 minutes]); at 2, 4, 6, 8, 12, 18, 24, 36, and 48 hours post-dose; on Day 7; again on Day 8 within 60 minutes before study drug infusion, at mid-infusion (0.5 hours), and at EOI (1 hour [+5 minutes]); at 2, 4, 6, 8, 12, 18, 24, 36, and 48 hours post-dose; and during visits on Days 14, 21 and 28 post-dose. All sampling time points are relative to the start of study drug infusion.

[o] Blood samples for the determination of serum (mRNA expressed) CHIKV24 IgG concentrations will be collected on Day 1 within 60 minutes before study drug infusion; at mid-infusion (0.5 hours), and at EOI (1 hour [+5 minutes]); at 2, 4, 6, 8, 12, 18, 24, 36, and 48 hours post-dose; on Day 7; again on Day 8 within 60 minutes before study drug infusion; at mid-infusion (0.5 hours); at EOI (1 hour [+5 minutes]); at 2, 4, 6, 8, 12, 18, 24, 36, and 48 hours post-dose; during visits on Days 14, 21 and 28 post-dose; and at Weeks 8, 12, 24, 36, 48, and 52 post-dose. Time points are relative to the start of study drug infusion.

[p] Blood samples for the determination of anti-PEG antibodies will be collected within 60 minutes before study drug infusion on Day 1; on Day 7; within 60 minutes before study drug infusion on Day 8; during visits on Days 14, 21, and 28 post-dose; and at Weeks 8, 12, 24, 36, 48, and 52 post-dose. Time points are relative to the start of study drug infusion.

[q] Blood samples for the determination of anti-CHIKV24 IgG antibodies will be collected within 60 minutes before study drug infusion on Day 1; on Day 7; within 60 minutes before study drug infusion on Day 8; during visits on Days 14, 21, and 28 post-dose; and at Weeks 8, 12, 24, 36, 48, and 52 post-dose. Time points are relative to the start of study drug infusion.

[r] Blood samples for determination of in vitro serum neutralizing antibody titer against a clinically relevant strain of CHIKV will be collected within 60 minutes before study drug infusion on Day 1; at 12, 24, and 48 hours post-dose; within 60 minutes before study drug infusion on Day 8; and at 12, 24, and 48 hours post-dose. Timepoints are relative to the start of study drug infusion.

[s] Prior medications taken by the subject within the 30 days before providing informed consent will be collected. Concomitant medications include all medications taken by the subject from the time of signing the informed consent and through the Week 52 visit.

[t] Adverse events will be assessed from the time of informed consent until EOS/ET and should be followed until they resolve, stabilize, or are judged by the investigator to be not clinically significant. All AEs will be recorded through 28 days after study drug infusion on Day 8, all medically attended AEs will be recorded for 3 months after study drug infusion, and all SAEs and AESIs (Section 7.4.1.3) will be recorded through Week 52.

[u] Blood samples for the assessment of complement factors C5b-9 will be collected on Day 1 pre-dose (within 60 minutes before study drug infusion) and at 90 (±5) minutes (from the start of infusion); at 2, 6, 12, 24 (±15 minutes), and 48 hours (±15 minutes) hours post-dose (completion of the infusion); on Day 8 predose (within 60 minutes before study drug infusion) and at 90 (±5) minutes (from the start of infusion); at 2, 6, 12, and 24 (±15 minutes) hours post-dose (completion of the infusion); at the time of discharge (within 60 minutes) from the inpatient unit. If the subject experiences an infusion reaction, a blood sample for analysis of complement factors should be obtained within 1 hour of the start of the reaction.

[v] Blood samples for the assessment of CRP will be collected on Day 1 pre-dose (within 60 minutes before study drug infusion) and 6-, 24-, and 48-hours post-dose (completion of infusion), and on Day 8 pre-dose (within 60 minutes before study drug infusion) and 6-, and 24-hours post-dose (completion of infusion); and at the time of discharge (within 60 minutes) from the inpatient unit. Blood samples for IL-6 and IP-10 will be collected on Day 1 pre-dose (within 60 minutes before study drug infusion) and at 90 minutes (±5) (from the start of infusion); at 2, 6, 12, 24 (±15 minutes), and 48 (within 60 minutes) hours postdose (completion of the infusion); on Day 8 pre-dose (within 60 minutes before study drug infusion) and at 90 minutes (±5) (from the start of infusion); at 2, 6, 12, and 24 (±15 minutes); and at the time of discharge (within 60 minutes) from the inpatient unit. If the subject experiences an infusion reaction, a blood sample for analysis of CRP and cytokine factors should be obtained within 1 hour of the start of the reaction.

2. INTRODUCTION

2.1 Background

Chikungunya virus (CHIKV) is a positive-sense, single-stranded RNA virus, of the alphavirus family and in the genus of the togavirus family. It is a mosquito-borne alphavirus that poses a significant public health problem in tropical and subtropical regions. While chikungunya has been present in Africa for centuries, it has more recently caused outbreaks and epidemics in new regions, thus reflecting the increasing distribution of the *Aedes* mosquito. In 2004, a chikungunya epidemic that started in Kenya spread through the Indian Ocean islands and India and was subsequently exported to nearly all regions of the world via infected travelers, resulting in millions of infected individuals, which brought chikungunya to the attention of the western world. As of April 2016, chikungunya cases had been reported in 103 countries and territories around the world, including 46 countries and territories throughout the Americas (Centers for Disease Control and Prevention 2016). Between March of 2016 and February of 2018, Chikungunya outbreaks have occurred in the US, Kenya, Italy, France, and Argentina (World Health Organization 2017) with over 1 million cases of chikungunya reported in the Americas in 2017 (PAHO 2017). There are an estimated 3 million cases of chikungunya globally.

Chikungunya virus infection causes chikungunya, an illness characterized by acute onset of fever, rash, myalgia, and debilitating polyarthralgia (Schwartz and Albert 2010; Weaver et al 2012). An additional hallmark feature of Chikungunya disease is a debilitating and prolonged arthralgic syndrome, primarily affecting the peripheral small joints. The pain associated with Chikungunya infection of the joints persists for months causing significant economic and social impact on both the individual and the affected communities (Powers et al 2007). While it is rarely fatal (1 in 1000 cases), neurological sequelae such as Guillain-Barre syndrome and chronic arthritides have been increasingly recognized.

Although the detailed mechanism of protection against CHIKV infection in humans is not completely understood, it is clear that neutralizing antibodies play an important role (Couderc et al 2009). Natural CHIKV infection of humans induces high neutralizing antibody titers (Lanciotti et al 2007), and immunoglobulin G (IgG) levels correlate with viral clearance and protection (Kam et al 2012a, Kam et al 2012b). Human CHIKV-specific neutralizing monoclonal antibodies have also been isolated and protect animals from experimental CHIKV infection (Smith et al 2015). The majority of these monoclonal antibodies recognize an important envelope glycoprotein (E2) and are able to cross-neutralize CHIKV from all genotypes (Smith et al 2015). Passive immunotherapy of convalescent sera has also been shown to protect animals from CHIKV infection (Partidos et al 2011).

Currently, no vaccines or therapeutics are approved for the prevention of, or as treatment for, disease associated with CHIKV infection. ModernaTX, Inc. (Sponsor) has developed messenger RNA (mRNA)-1944, a novel, intravenous (IV) administered, lipid-encapsulated mRNA encoding a human neutralizing monoclonal antibody against a chikungunya viral protein. The proposed indication for the administration of mRNA-1944 is passive immunization of individuals via the production of circulating human antibody IgG1 levels that are anticipated to prevent chikungunya infection and the consequent disease for a minimum of 2 months.

2.2 Nonclinical studies in Development of mRNA-1944

In support of development of mRNA-1944 and the initiation of a Phase 1, single ascending dose clinical trial in heathy adults, several nonclinical pharmacology, distribution, toxicology, and safety studies have been performed.

mRNA-1944 encodes a fully human IgG (CHIKV24, antibody clone expressed by mRNA-1944) isolated from the B cells of a patient with prior history of chikungunya infection. Variable region amino acid sequences of clone CHIKV24 were used to construct the heavy and light chain mRNA sequences in mRNA-1944 on an IgG1 backbone. This antibody has an affinity for chikungunya envelope E2 protein and very high in vitro neutralizing activity against virus strains from African, Asian, and American lineages (EC_{50} of < 10 ng/mL).

The prophylactic efficacy of recombinant ChikV24 IgG protein and ChikV24 mRNA was demonstrated in nonclinical proof-of-concept primary pharmacology studies in a lethal mouse model of CHIKV infection in AG129 mice, which lack the IFN α/β and γ receptors and are acutely sensitive to CHIKV infection. The expression of total hIgG was evaluated in plasma after a single IV dose of mRNA-1944 at 0.1 mg/kg and 0.5 mg/kg in cynomolgus monkeys. Quantification of functional ChikV24 IgG was measured 24 hours after a single IV infusion of mRNA-1944 at 0.5 mg/kg in male cynomolgus monkeys by a neutralization assay (PRNT50) and ELISA. The cardiovascular safety of mRNA-1944 was demonstrated in a Good Laboratory Practice (GLP)-compliant cardiovascular safety pharmacology study in telemetered cynomolgus monkeys.

The tissue persistence and distribution of mRNA-1944 was evaluated in a non-GLP tissue distribution study in male Sprague Dawley rats. The expression of hIgG was evaluated in plasma after a single IV infusion of mRNA-1944 at 0.3 and 0.6 mg/kg in Sprague Dawley rats.

To help identify the potential cross-reactivity of ChikV24 IgG antibody with other tissues or organ systems, ChikV24 IgG was evaluated in a GLP-compliant study using cryosections of human, cynomolgus monkey, and Sprague Dawley rat tissues.

The safety and tolerability of mRNA-1944 were evaluated in a GLP-compliant repeat-dose (1 week, 2 doses) toxicity study with a 4-week recovery period in Sprague Dawley rats and a

GLP-compliant repeat-dose (1 week, 2 doses) toxicity study with a 12-week recovery period in the cynomolgus monkey.

Repeat administration of mRNA-1944 by IV infusion on Day 1 and Day 8 was well tolerated in rats up to 5.0 mg/kg/dose. Target organ effects were limited to the spleen at ≥ 1.75 mg/kg/dose. At the end of the recovery period, all changes were fully recovered. The no observed adverse effect level (NOAEL) for mRNA-1944 in Sprague Dawley rats was considered to be 5.0 mg/kg/dose, based on the low severity, incidence, and reversibility of the target organ effects and microscopic findings at this dose.

Administration of mRNA-1944 by IV infusion on Day 1 and Day 8 was well tolerated in cynomolgus monkeys up to 3.0 mg/kg/dose. mRNA-1944-related findings were limited to increased splenic weight without microscopic correlate at ≥ 1.0 mg/kg/dose, infusion site reaction at all doses, and increases in MCP-1 and/or complement factors, suggestive of mild systemic inflammation at all doses. With the exception of minimal fibrosis at the infusion site observed in 1 recovery animal at the end of the recovery period, all changes were considered recovered. The NOAEL for mRNA-1944 in cynomolgus monkeys was considered to be 3.0 mg/kg/dose, based on the low incidence, low severity, and reversibility of the target organ effects and systemic inflammation at this dose.

The LNPs of mRNA-1944 contain 4 lipid components including a proprietary high purity polyethylene glycol-2k-stearate monoester, IAL (a proprietary ionizable amino lipid), cholesterol, and 1,2-distearoyl-sn-glycero-3-phosphocholine [DSPC]). The proprietary polyethylene glycol-2k-stearate monoester and IAL are novel lipid excipients that are not contained in approved medicinal products, whereas both cholesterol and DSPC have been well characterized and are incorporated into approved products intended for parenteral delivery. Therefore, the polyethylene glycol-2k-stearate monoester and IAL, the novel lipids used in the mRNA-1944 formulation, were evaluated as individual agents for mutagenic and clastogenic activity in genotoxicity studies using a standard International Council for Harmonisation (ICH) S2 (R1) approach, including GLP-compliant bacterial reverse mutation (Ames) tests and GLP-compliant in vitro micronucleus tests. Both polyethylene glycol-2k-stearate monoester and IAL were negative in the bacterial reverse mutation test and in vitro micronucleus test. Additionally, to support the use of these novel lipids in the formulated drug product, a GLP-compliant in vivo micronucleus test conducted in Sprague Dawley rats administered a single IV injection of mRNA-1944 showed that mRNA-1944 did not induce chromosomal damage in rat bone marrow immature erythrocytes, when tested up to 20 mg/kg of mRNA-1944 (26.5 mg/kg of high-purity polyethylene glycol-2k-stearate monoester and 204.0 mg/kg of IAL), in accordance with regulatory guidelines. The absence of an in vivo response to mRNA-1944 and in vitro responses to polyethylene glycol-2k-stearate monoester and IAL indicates that the risk of mRNA-1944 genotoxicity in patients is low.

A detailed review of the nonclinical experience with mRNA-1944 is provided in the Investigator's Brochure.

2.3 Clinical Studies with mRNA-1944

This is a first-in-human (FIH) study. No previous clinical studies have been performed.

2.4 Rationale for Study and Study Design

2.4.1 Rationale for Study

Currently, no vaccines or therapeutics are approved for the prevention of, or as treatment for, the disease associated with CHIKV infection. mRNA-1944 is a novel, IV administered, lipid-encapsulated, mRNA which encodes a human neutralizing monoclonal antibody against a chikungunya viral protein. The proposed indication for the administration of mRNA-1944 is passive immunization of individuals via the production of circulating human antibody IgG1 levels that are anticipated to prevent chikungunya infection and the consequent disease.

2.4.2 Rationale for Study Population and Design

As a FIH study, study treatment will be administered at the study site to healthy adult volunteers. A placebo group will be used as a control for the safety, pharmacokinetic (PK), and pharmacodynamic (PD) assessments.

As mRNA-1944 will be administered for the first time to humans, safety precautions such as sequential enrollment, premedication, dose escalation, and continuous safety evaluations will be taken. During each cohort, mRNA-1944 will be initially administered to 1 subject at a time for the first 3 subjects (sentinel dosing), and then, following the confirmation of acceptable safety and tolerability, enrollment will be expanded and the remaining 5 subjects within each dose level cohort will be randomly assigned 3:2 to receive mRNA-1944 or placebo. Study pause rules are defined (Section 4.2.5), and the safety evaluation from this study will be overseen by an internal safety team (IST) and an unblinded independent safety monitoring committee (SMC). Additional information regarding the IST and SMC is provided in Section 4.2.2.

2.4.3 Rationale for Dose Selection

The clinical safety and tolerability of mRNA-1944 have yet to be determined. The proposed dose levels of mRNA-1944 to be evaluated in this study (0.1, 0.3, 0.45 [optional], 0.6, and 1.0 mg/kg) are based on nonclinical pharmacology and safety studies (Section 2.2) in mice, rats, and nonhuman primates.

The starting dose of this Phase 1 study is one tenth of the human equivalent dose, using the NOAEL identified in the most sensitive toxicology species.

3. STUDY OBJECTIVES

3.1 Primary Objective

The primary objective of this study is to evaluate the safety and tolerability of escalating doses of mRNA-1944 administered via IV infusion in subjects 18 to 50 years of age.

3.2 Secondary Objectives

The secondary objectives of this study *are the following:*

- To determine the PK of mRNA encoding for CHIKV24 IgG (heavy and light chain mRNA) and IAL
- *To determine the PD of mRNA-1944 as assessed by CHIKV24 IgG*

3.3 Exploratory Objectives

The exploratory objectives of this study are the following:

- To evaluate the formation of anti-polyethylene glycol (PEG) antibodies
- To evaluate the formation of anti-CHIKV24 IgG antibodies
- To evaluate the impact of several baseline characteristics on the PK/PD of mRNA encoding for CHIKV24 IgG and IAL
- To evaluate the in vitro serum neutralizing antibody against a clinically relevant strain of CHIKV
- To evaluate complement and acute phase reactant parameters

4. INVESTIGATIONAL PLAN

4.1 Study Design

This is a Phase 1, FIH, single-center, randomized, investigator-blinded, placebo-controlled, dose-escalation study to evaluate the safety, tolerability, PK, and PD of mRNA-1944 in healthy adult subjects. Investigator blind means the investigator, study subjects, site monitors, and study site personnel will be blinded to the study drug administered with the following exceptions: the 3 sentinel subjects in each dose level cohort are not blinded, unblinded pharmacy personnel, unblinded study monitor, unblinded clinical trial manager, and an unblinded team to provide safety data to the SMC.

The study flow diagram is presented in Figure 4-1.

Figure 4-1 Study Flow Diagram

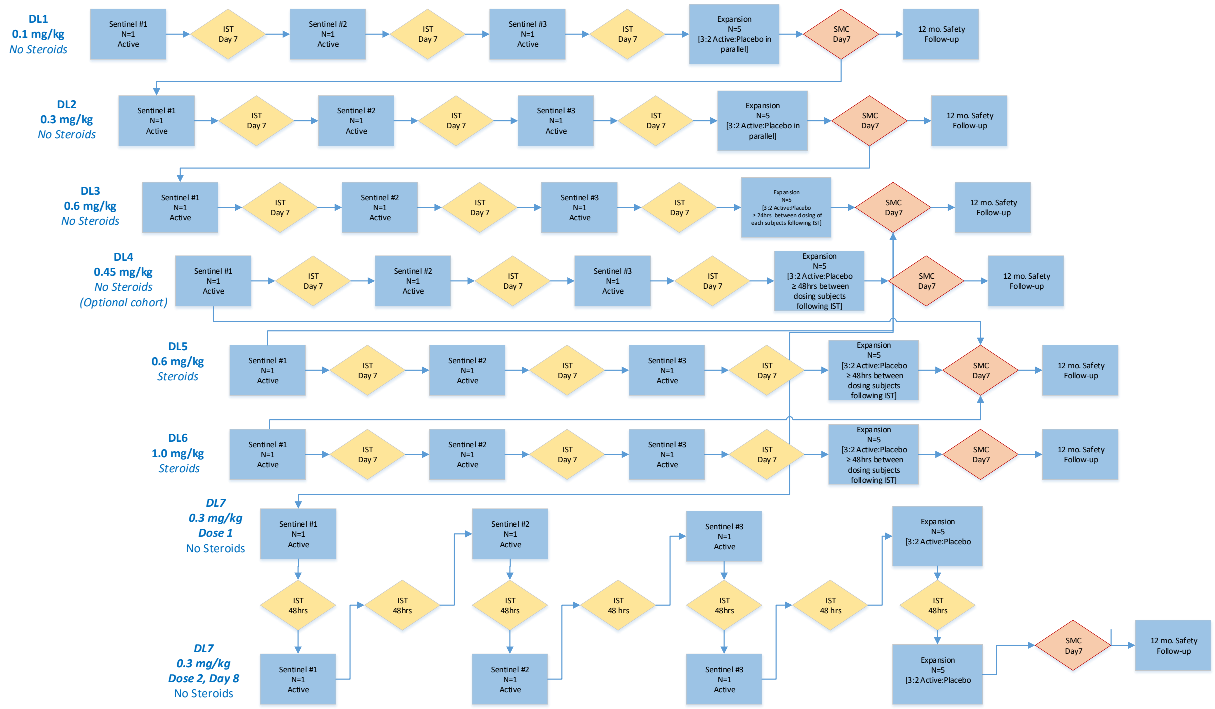

Abbreviations: DL, dose level; hrs, hours; IST, internal safety team; mo, month; SMC, safety monitoring committee.

Approximately 56 subjects (8 per cohort) are planned to be enrolled (including the optional intermediate dose level cohort of 0.45 mg/kg); 6 subjects will receive mRNA-1944 and 2 subjects will receive placebo within each cohort.

Among the 6 subjects receiving mRNA-1944, 3 are the sentinel subjects. The remaining 5 subjects within each dose level cohort will be randomly assigned 3:2 to receive mRNA-1944 or placebo.

Subjects in the expansion group of dose level cohorts 5 and 6 (and optional dose level cohort 4) will be enrolled with a minimum of 48 hours between the 5 subjects. For expansion subject enrollment in dose level cohorts 5 and 6 (and optional dose level cohort 4), the IST will meet approximately 48 hours after each subject in the expansion group completes their infusion to review the clinical course and safety data of the immediate post-infusion period and to approve the dosing of the next expansion cohort subject. Subjects in the expansion group of dose level cohort 7 will be enrolled and dosed in parallel.

Seven cohorts of mRNA-1944 are planned to be investigated in a dose-escalation manner; one of the dose level cohorts, the 0.45 mg/kg dose level cohort, is an optional intermediate dose level cohort. The first 3 dose level cohorts are doses of 0.1, 0.3, and 0.6 mg/kg and these dose level cohorts are dosed without dexamethasone included in the premedication regimen. In order to enable a more robust characterization of the PK, PD, and adverse reaction profile and to ensure that all subjects in a complete dose level cohort receive the identical premedication regimen a second dose level cohort at the 0.6 mg/kg dose level has been added. This dose level cohort will receive the prespecified premedication regimen including criteria of when steroids should be added to the premedication regimen. Dexamethasone premedication (10 mg, IV) will be added to the premedication regimen for all subjects in the 0.6 mg/kg and 1.0 mg/kg dose level cohorts (and the 0.45 mg/kg optional dose level cohort).

Subjects in dose level cohort 7 will be administered two IV infusions of 0.3 mg/kg, one infusion on Day 1 and another subsequent infusion on Day 8. The premedication regimen for dose level cohort 7 will include diphenhydramine (50 mg, oral) and famotidine (20 mg, oral) and will be administered prior to the planned start of study drug infusion on Days 1 and 8. Dexamethasone will not be included in the premedication regimen for dose level cohort 7.

Study drug will be administered to dose level cohorts 1, 2, sentinel subjects in dose level cohort 3, 4, 5, 6, and 7 as a single IV infusion over 1 hour by a controlled infusion device. Study drug will be administered to the expansion subjects in dose level cohort 3 as a single IV infusion over approximately 3 hours by a controlled infusion device. The infusion time may be further extended up to 4 hours in the event of an infusion reaction or the occurrence of an adverse reaction assessed as related to infusion of the study drug experienced by any subject in a given cohort.

Subjects in dose level cohorts 1 through 6, will remain inpatient at the study site to be observed for safety assessments and PK/PD sampling for 48-hours following completion of dosing on Day 1. Subjects in dose level cohort 7, will remain inpatient at the study site from Day –1, prior to the first dose of the 2-dose regimen, through 48-hours following completion of the second dose on Day 8. All subjects in dose level cohort 7 will be observed for safety assessments and undergo PK/PD sampling for 48-hours following completion of dosing of each of the 0.3 mg/kg doses. Adverse events (AEs) will be captured through the study follow-up period of 12 months and additional plasma PK samples will be collected through end of study (Week 52; 12 months). Optional dose level cohorts for intermediate dose levels may be added for de-escalation of the dose after review of cumulative data by the SMC. The Sponsor may stop further dose escalation once pharmacologic goals have been achieved.

Consent and screening will occur over the 28-day period prior to Day 1. All subjects will be screened for the presence of antibodies (IgG) to CHIKV, and those subjects who are positive will be excluded from participation in the study. Eligible subjects will be admitted to the clinic the day prior to study drug infusion. Within each dose level cohort, 6 subjects will receive mRNA-1944 and 2 subjects will receive placebo. Among the 6 subjects receiving mRNA-1944, 3 are the sentinel subjects. The remaining 5 subjects in each dose level cohort will be randomly assigned 3:2 to receive mRNA-1944 or placebo. This study will be conducted with the oversight of both an IST and an independent SMC (Section 4.2.2).

A subject may be rescreened for study eligibility if their originally intended sentinel or expansion group is filled and their 28-day screening window is surpassed before another group opens. The subject will be assigned a new screening number and all screening procedures will be repeated. Subjects who did not meet all enrollment criteria at their first screening will not be allowed to rescreen. Screen failures are defined as subjects who sign the consent form but who are not subsequently randomly assigned to the study intervention or entered in the study. Information on

eligibility, demographics, serious AEs (SAEs), and informed consent will be collected for all screen failures.

All subjects will be premedicated prior to dosing with mRNA-1944 or placebo. Premedication and the management of any suspected infusion-related reactions (IRR) will be implemented as described below. Suspected IRRs will be assessed according to the National Cancer Institute Common Terminology Criteria for Adverse Events Version 5 (CTCAE 2017) and will include reactions assessed as related to study drug occurring during or within 24 hours after the infusion.

- On Day 1, all subjects in dose level cohorts 1 and 2 and sentinel subjects in dose level cohort 3 will be premedicated with loratadine (10 mg, oral) and ranitidine (150 mg, oral) approximately 90 minutes prior to the planned start of study drug infusion.
- On Day 1, all subjects in the expansion group of dose level cohort 3 will be premedicated with loratadine (10 mg, oral), ranitidine (150 mg, oral), and acetaminophen (650 mg, oral) approximately 90 minutes prior to the planned start of study drug infusion.
- On Day 1, all subjects in dose level cohorts 5 and 6 (and the optional dose level cohort 4) will be premedicated with dexamethasone (10 mg, IV), diphenhydramine (50 mg, oral), and famotidine (20 mg, oral) approximately 90 minutes prior to the planned start of study drug infusion.
- On Day 1 and Day 8, all subjects in dose level cohort 7 will be premedicated with diphenhydramine (50 mg, oral) and famotidine (20 mg, oral) approximately 90 minutes prior to the planned start of study drug infusion.
- Should any 1 subject develop a grade 2 IRR, their infusion will be slowed or briefly interrupted until resolution of symptoms.

Note: Management of IRRs should be based on the grade of the reaction and include slowing the rate, or temporary interruption, of the infusion (up to 4 hours). Mild to moderate IRRs (ie, CTCAE v5 grades 1 and 2 and infusion reactions that do not involve symptoms of anaphylaxis) can usually be managed with slowing, or temporary interruption, of the infusion and symptom management with the administration of additional antihistamines, antipyretics, and/or corticosteroids. Per judgment of the investigator, after all symptoms have resolved, the infusion may be resumed at a reduced rate with the administration of additional premedications as indicated.

- Because 1 subject in the 0.6 mg/kg dose level cohort (after receiving the oral premedication regimen) developed a persistent grade 2 or higher IRR all subsequent subjects in the study who receive >0.3 mg/kg as a single dose will be administered dexamethasone (10 mg, IV), in addition to the above oral regimen, as premedication prior to study drug infusion.

- To monitor for potential IRR, vital signs will be assessed at check-in, approximately every 15 minutes during study drug the infusion, and every 15 minutes thereafter for the first hour after completion of the infusion. Continuous pulse oximetry monitoring will be implemented for the duration of the study drug infusion Section 1).
- Should any 1 subject develop a grade 3 or higher IRR (including an allergic, hypersensitivity, or anaphylactic reaction), study drug infusion will be immediately and permanently discontinued for this subject. Such an occurrence constitutes a pause criterion and no further dosing can occur until reviewed by the SMC.

Reactions with any features of anaphylaxis (such as shortness of breath, urticaria, or angioedema) or severe IRRs (CTCAE v5 grade 3 or higher) require immediate and permanent discontinuation of the drug infusion for that subject and the convening of an SMC meeting. Suspected allergic (hypersensitivity) reactions and anaphylaxis will be assessed according to the clinical diagnostic criteria outlined by the National Institute of Allergy and Infectious Diseases (Section 10.5) and CTCAE v5.

Subjects in dose level cohorts 1 through 6 will be monitored inpatient for 48 hours following the end of infusion (EOI) on Day 1 and followed on an outpatient basis for approximately 12 months. Subjects in dose level cohort 7, will be monitored inpatient prior to the first dose on Day 1, through 48 hours following completion of the second dose on Day 8 and followed up on an outpatient basis for approximately 12 months.

All subjects will return to the clinic for visits on Days 7 (±2) (dose level cohorts 1 through 6), 14 (±2), 21 (±2), and 28 (±3), and Weeks 8 (±1), 12 (±2), 24 (±2), 36 (±2), 48 (±2), and 52 (±2) after the study drug infusion.

Safety assessments will include monitoring and recording of AEs and prior and concomitant medication, clinical laboratory test results, electrocardiogram (ECG) results (and cardiac enzymes when obtained per protocol), vital sign measurements, and physical examination findings.

Blood samples for the determination of serum mRNA-1944 encoding for CHIKV24 IgG and plasma IAL concentrations will be collected for all subjects in dose level cohorts 1, 2, 4, 5, and 6 and for the sentinel subjects in dose level cohort 3 within 60 minutes before study drug infusion; at mid-infusion (0.5 hours); at EOI (1 hour [+5 minutes]); at 2, 4, 6, 8, 12, 18, 24, 36, and 48 hours post-dose; and during visits on Days 7, 14, 21 and 28 post-dose. For the 3 expansion subjects in dose level cohort 3, blood samples will be collected within 60 minutes before study drug infusion; at mid-infusion (1.5 hours); at EOI (3 hours [+5 minutes]); at 4, 6, 8, 12, 18, 24, 36, and 48 hours post-dose; and during visits on Days 7, 14, 21, and 28 post-dose. For subjects in dose level cohort 7, blood samples will be collected on Day 1 within 60 minutes before study drug infusion; at mid-infusion (0.5 hours), and at EOI (1 hour [+5 minutes]); at 2, 4, 6, 8, 12, 18, 24, 36, and

48 hours post-dose; on Day 7; again on Day 8 within 60 minutes before study drug infusion, at mid-infusion (0.5 hours), and at EOI (1 hour [+5 minutes]); at 2, 4, 6, 8, 12, 18, 24, 36, and 48 hours post-dose; and during visits on Days 14, 21 and 28 post-dose. All of these time points are with respect to the start of infusion (for the 2-, 4-, 6-, 8-, 12-, and 18-hour time points, a window of ±15 minutes is acceptable; for the 24-, 36-, and 48--hour time points, a window of ±30 minutes is acceptable).

Note: if the total infusion time is extended beyond 1 hour the blood sample should be collected at EOI; and if it overlaps with pre-specified time points sampling should continue at the next scheduled time point relative to the start of infusion.

Blood samples for the determination of serum (mRNA expressed) CHIKV24 IgG concentrations, will be collected for all subjects in dose level cohorts 1, 2, 4, 5, and 6 and for the sentinel subjects in dose level cohort 3 within 60 minutes before study drug infusion; at mid-infusion (0.5 hours); at EOI (1 hour [+5 minutes]); at 2, 4, 6, 8, 12, 18, 24, 36, and 48 hours post-dose; during visits on Days 7, 14, 21, and 28 post-dose; and at Weeks 8, 12, 24, 36, 48, and 52 post-dose. For the 3 expansion subjects in dose level cohort 3 blood samples will be collected within 60 minutes before study drug infusion; at mid-infusion (1.5 hours), at EOI (3 hours [+5 minutes]); at 4, 6, 8, 12, 18, 24, 36, and 48 hours post-dose; during visits on Days 7, 14, 21, and 28 post-dose; and at Weeks 8, 12, 24, 36, 48, and 52 post-dose. For subjects in dose level cohort 7, blood samples will be collected on Day 1 within 60 minutes before study drug infusion; at mid-infusion (0.5 hours), and at EOI (1 hour [+5 minutes]); at 2, 4, 6, 8, 12, 18, 24, 36, and 48 hours post-dose; on Day 7; again on Day 8 within 60 minutes before study drug infusion, at mid-infusion (0.5 hours); and at EOI (1 hour [+5 minutes]); at 2, 4, 6, 8, 12, 18, 24, 36, and 48 hours post-dose; during visits on Days 14, 21 and 28 post-dose; and at Weeks 8, 12, 24, 36, 48, and 52 post-dose. All of these time points are with respect to the start of infusion (for the 2-, 4-, 6-, 8-, 12-, and 18-hour time points, a window of ±15 minutes is acceptable; for the 24-, 36-, and 48-hour time points, a window of ±30 minutes is acceptable).

Blood samples for the determination of anti-PEG antibodies will be collected for dose level cohorts 1 through 6 within 60 minutes before study drug infusion; during visits on Days 7, 14, 21, and 28 post-dose; and at Weeks 8, 12, 24, 36, 48, and 52 post-dose. For dose level cohort 7, these samples will be collected within 60 minutes before study drug infusion on Day 1; on Day 7; within 60 minutes before study drug infusion on Day 8; during visits on Days 14, 21, and 28 post-dose; and at Weeks 8, 12, 24, 36, 48, and 52 post-dose

Blood samples for the determination of anti-CHIKV24 IgG antibodies will be collected for dose level cohorts 1 through 6 within 60 minutes before study drug infusion; during visits on Days 7, 14, 21, and 28 post-dose; and at Weeks 8, 12, 24, 36, 48, and 52 post-dose. For dose level cohort 7, these samples will be collected within 60 minutes before study drug infusion on Day 1; on Day 7;

within 60 minutes before study drug infusion on Day 8; during visits on Days 14, 21, and 28 postdose; and at Weeks 8, 12, 24, 36, 48, and 52 post-dose.

Blood samples for the determination of in vitro serum neutralizing antibody titer against a clinically relevant strain of CHIKV will be collected for dose level cohorts 1 through 6 within 60 minutes before study drug infusion and at 12, 24, and 48 hours post-dose. For dose level cohort 7, these samples will be collected within 60 minutes before study drug infusion on Day 1; at 12, 24, and 48 hours post-dose; within 60 minutes before study drug infusion on Day 8; and at 12, 24, and 48 hours post-dose.

A blood sample for DNA isolation in order to determine genotyping and to determine the concentration levels of Apo E will be collected within 60 minutes before study drug infusion on Day 1 from subjects who have consented to participate in the genetic analysis component of the study.

Retained Samples for Future Analysis:

Retained serum and plasma samples may be used to further understand immune responses to the treatment or for further research related to vaccines against CHIKV and/or other infectious diseases.

4.2 Dosing

4.2.1 Sentinel Dosing Strategy

Eight subjects are planned to be enrolled at each dose level cohort. For each dose level cohort, a sentinel dosing strategy will be employed.

For dose level cohorts 1 through 6, a safety group of 3 subjects will be enrolled, all will receive mRNA-1944, with a staggered minimum 7-day interval between each subject prior to treating the remaining subjects within a dose cohort. Each sentinel subject will be followed for 7 days after the study drug infusion (the first 48 hours will be inpatient) with an IST review of the safety results through 7 days following the study drug infusion prior to the enrollment of each subsequent sentinel subject. The IST will then review all safety data, including cumulative safety data, on all sentinel subjects prior to randomly assigning the remainder of dose level cohort (5 subjects, 3 active:2 placebo).

In dose level cohort 7, a safety group of 3 subjects will be enrolled; all will receive two doses of 0.3 mg/kg, one infusion on Day 1 and another subsequent infusion on Day 8. Each sentinel subject will remain inhouse from Day –1 to Day 10 (48 hours after the second 0.3 mg/kg infusion). The IST will review the safety results approximately 48 hours after the first 0.3 mg/kg dose to approve receipt of the second 0.3 mg/kg dose. The IST will also review data 48 hours after each sentinel

subject receives their second dose to approve dosing of the subsequent sentinel subject (second and third sentinel subjects). The IST will also review all safety data on all sentinel subjects approximately 48 hours after the third sentinel subject receives their second dose and approve dosing of the expansion subjects (5 subjects, 3 active:2 placebo). The 5 expansion subjects will be dosed in parallel.

4.2.2 Internal Safety Team and Safety Monitoring Committee

Two safety monitoring boards, the IST, and an independent unblinded SMC, will be organized to oversee the safety of the study. Details regarding composition, responsibilities, and procedures of the IST and SMC will be outlined in their respective charters.

Internal Safety Team

The IST will include the site principal investigator, the Sponsor medical monitor, and an additional Sponsor physician representative. The IST will oversee the safety of the study and will evaluate ongoing safety data throughout the study to ensure adherence to the protocol. For dose level cohorts 1 through 6, the IST will monitor safety data to approve dosing of the second and third sentinel subjects in each dose level cohort, to determine advancement to dose expansion in each dose level cohort, and to approve the dosing of each subject in the expansion group after review of the clinical course and safety data approximately 24 hours after each subject in the expansion group of dose level cohort 3 completes their infusion and approximately 48 hours after each subject in the expansion group of dose level cohorts 5 and 6 (and optional dose level cohort 4) completes their infusion (Section 4.2.3). The IST will review cumulative safety data weekly during the active enrollment of subjects into dose level cohorts in addition to reviewing prior to each dose level expansion.

For dose level cohort 7, the IST will review safety data on each sentinel subject approximately 48 hours after receipt of the first 0.3 mg/kg dose and approve receipt of the second 0.3 mg/kg dose on Day 8. The IST will review safety data approximately 48 hours after each sentinel subject receives their second 0.3 mg/kg dose to approve dosing of the subsequent sentinel subject (second and third sentinel subjects). The IST will also review safety data on all sentinel subjects approximately 48 hours after the third sentinel subject receives their second dose and approve dosing of the expansion group (5 subjects). The 5 expansion subjects will be dosed in parallel. The IST will review safety data approximately 48 hours after all 5 expansion subjects receive their first 0.3 mg/kg dose to approve dosing of the second 0.3 mg/kg dose. Cohort 7 may run in parallel with dose level cohort 5, regardless of whether dosing has begun in cohort 6.

If the IST determines that a pre-established pause rule (Section 4.2.5) is met or identifies any other potential safety concern, the independent unblinded SMC will be notified. In such cases, study enrollment and all further dosing will be paused.

Safety Monitoring Committee

The study will be overseen by an unblinded SMC. The role of the SMC includes the review and protection of data integrity and safety of study subjects throughout the study period. The independent SMC will review ongoing safety data, including cumulative safety data from all study subjects. The independent SMC will determine if it is acceptable to dose escalate (Section 4.2.4) per their safety review charter after each dose level cohort based on pre-established pause rules (Section 4.2.5). The SMC may also be convened ad hoc to support the conduct of the study. For any enrollment pauses to the study that are triggered by the pause rules, the SMC will be convened to determine whether treatment may be resumed, whether any revisions to the protocol are warranted, or if the study should be stopped.

4.2.3 Dose Expansion

After review by the IST of the safety data through 7 days after the study drug infusion of all sentinel safety lead-ins for each dose level cohort, approval may be given to proceed with enrollment of the remainder of that dose level cohort. Subjects in the expansion group of dose level cohorts 1, 2, and 3 will be enrolled with a minimum of 24 hours between each of the 5 expansion group subjects. Subjects in the expansion group of dose level cohorts 5 and 6 (and optional dose level cohort 4) will be enrolled with a minimum of 48 hours between each of the 5 expansion group subjects. Subjects in the expansion group of dose level cohort 7 will be enrolled and dosed in parallel.

4.2.4 Dose Escalation

Seven dose level cohorts of mRNA-1944 are planned to be investigated in this study (one optional). The SMC will review unblinded safety data of the entire currently dosed cohort through 7 days after the study drug infusion, and cumulative safety data from all study subjects prior to escalation to the next dose level. Dose escalation to higher dose level cohorts may continue until a study pause rule is reached (Section 4.2.5). Optional dose level cohorts for intermediate dose levels, eg, a 0.45 mg/kg dose level, may be added for de-escalation of the dose after review of cumulative data by the SMC. The Sponsor may stop further dose escalation once pharmacologic goals have been achieved.

4.2.5 Pause Rules

The occurrence of any of the following criteria results in a pause and no further dosing can occur until reviewed by the SMC:

- Any SAE, irrespective of assessed relatedness to study drug*.
- Two or more subjects experience a grade 3 or higher AE, including a laboratory abnormality, of the same nature.
- Any subject develops a grade 3 or higher IRR (including an allergic, hypersensitivity, or anaphylactic reaction).
- Any clinical event that, in the opinion of the SMC, is a contraindication to further dosing of additional subjects.

*Serious AEs that are the result of external causes and clearly not related to study drug, as assessed by both the investigator and the Sponsor, eg, subject involved in an automobile accident or a sports-related injury, will not result in a pause.

For any enrollment pauses to the study that are triggered by the pause rules, the SMC will be convened to determine whether treatment can be resumed, whether any revisions to the protocol are warranted, or if the study should be stopped.

4.3 End of Study Definition

The start of the study is defined as the first visit for the first subject screened. The end of study is defined as the last visit or last health status follow-up for the last subject discharged from the study. Study completion is defined as the final date on which data for the primary endpoint was or is expected to be collected, if the final date is not the same.

The approximate duration of participation for each subject will be 13 months, which includes a 28-day screening period.

5. STUDY POPULATION

Approximately *56* healthy male and female subjects will be enrolled at a *single* center.

5.1 Inclusion Criteria

Each subject must meet all of the following criteria to be enrolled in this study:

1. The subject is male or female ≥ 18 and ≤ 50 years of age.
2. The subject has a weight of 50 to ≤ 90 kg, inclusive and a maximum body mass index ≤ 33 kg/m^2.
3. The subject is considered by the investigator to be in good general health as determined by medical history, clinical laboratory assessments, ECG results, vital sign measurements, and physical examination findings at screening.
4. Female subjects of nonchildbearing potential may be enrolled in the study. Nonchildbearing potential is defined as bilateral tubal ligation >1 year prior to screening, bilateral

oophorectomy, or hysterectomy or menopause (Glossary of Terms). Follicle-stimulating hormone level may be measured, at the discretion of the investigator, to confirm menopausal status.

5. Female subjects of childbearing potential may be enrolled in the study, if the subject meets the following criteria: 1) has a negative pregnancy test at screening and at check-in, 2) has practiced adequate contraception (Glossary of Terms) or abstained from all activities that could lead to pregnancy for 30 days prior to study drug infusion, and 3) has agreed to continue adequate contraception through 36 weeks after study drug infusion.
6. Male subjects must agree to consistently use adequate contraception (Glossary of Terms) and refrain from sperm donation through 36 weeks after study drug infusion.
7. The subject understands and agrees to comply with the study procedures and provides written informed consent before any study procedures are performed.
8. The subject agrees to stay in contact with the study site for the duration of the study and to provide updated contact information, as necessary for the duration of the study.

5.2 Exclusion Criteria

Subjects meeting any of the following criteria will be excluded from the study:

1. Has an acute or chronic clinically significant disease, as determined by physical examination or laboratory screening tests. Asymptomatic conditions or findings (eg, mild hypertension, dyslipidemia) are not exclusionary if they are being appropriately managed, are clinically stable, and are unlikely to progress within the study period in the opinion of the investigator.
2. If female and of childbearing potential, has a positive serum pregnancy test at screening or on Day –1.
3. If female and of childbearing potential, is pregnant or lactating, has not adhered to an adequate contraception method for at least 30 days before study entry, or is unwilling to use adequate contraception for at least 36 weeks after the study drug infusion.
4. Has elevated liver function tests, defined as aspartate aminotransferase, alanine aminotransferase, or alkaline phosphatase, as well as elevated creatinine, or decreased platelets, with a toxicity score of grade 1 or higher at screening (Section 10.4). Retesting of these parameters is not allowed.
5. Has safety laboratory test results (hematology, chemistry, and coagulation) of grade 2 or higher at screening and baseline (Section 10.4). The inclusion of subjects with non-clinically significant grade 1 laboratory abnormalities (other than those noted in Exclusion Criterion 4) is allowed based on the investigator's discretion.
6. Has a positive screening test for the presence of anti-CHIKV IgG.

7. Has participated in another investigational study involving any investigational product (ie, study drug, biologic, device) within 60 days or 5 half-lives, whichever is longer, before the planned date of study drug infusion (Day 1).
8. Has received any live attenuated or inactive vaccines within 4 weeks prior to check-in or plans to receive any vaccine during the study; a seasonal influenza vaccine is permissible.
9. Has received (at any time) a vaccine for CHIKV, dengue, yellow fever, tick-borne encephalitis, or Japanese encephalitis.
10. Has previously participated in an investigational study involving lipid nanoparticles.
11. Has a known or suspected immune-mediated disease or immunosuppressive condition (including lymphoproliferative disorders) as determined by medical history and/or physical examination (Section 7.4.1.3).
12. Has a neurologic disorder (eg, history of seizures, Guillain-Barre syndrome, dementia, vasculitis, or any known congenital or acquired disorder).
13. Has a history of idiopathic urticaria.
14. Has any bleeding disorder that is considered a contraindication to study drug infusion or blood collection.
15. Has any medical, psychiatric, or occupational condition that, in the opinion of the investigator, might pose an additional risk to the subject if they participate in the study or might interfere with the evaluation of mRNA-1944 or the interpretation of study results.
16. Has received immunoglobulins, a monoclonal antibody or any blood products within the 6 months preceding administration of the study drug or plans to receive such products at any time during the study.
17. Has received heparin treatment within the last 30 days prior to study drug infusion or plans to receive any heparin treatment during the study.
18. Is currently receiving antipyretic or analgesic medication on a daily or every other day basis (a daily dose of ≤ 100 mg of aspirin given under the guidance of a physician is not a contraindication to enrollment).
19. Has any acute disease or fever at the time of screening. Fever is defined as body temperature ≥ 38.0°C/100.4°F by the oral, axillary, or tympanic route. Subjects with acute disease or fever at the time of screening may be re-evaluated at a later date. Subjects with a minor illness without fever can be enrolled at the discretion of the investigator (defined as the presence of a moderate or severe illness with or without fever, or an oral temperature > 38.0°C on the planned day of infusion). In such cases, subjects may be re-evaluated during the screening period for resolution of the illness to allow at least 3 days of wellness prior to the planned study drug infusion.
20. Has a positive urine drug screen for any of the following nonprescription drugs of abuse at screening or at check-in: alcohol, opiates, cocaine, phencyclidine, amphetamines,

benzodiazepines, or methadone. Positive drug screens for amphetamines, benzodiazepines, or opiates will not be exclusionary if prescribed concomitant medications can justify the result.

21. Has known allergies to any components or excipients of the study drug, as detailed in Section 6.2.
22. Has any condition that, in the opinion of the investigator, would pose a health risk to the subject if they enrolled or could interfere with evaluation of the study drug or the interpretation of study results (including neurologic or psychiatric conditions deemed likely to impair the quality of safety reporting).
23. Has a positive test result for hepatitis B surface antigen, hepatitis C virus antibody, or human immunodeficiency virus type 1 or 2 antibodies.
24. Has a history of active cancer (malignancy) in the last 3 years. An exception is a subject with adequately treated nonmelanomatous skin carcinoma, who may participate in the study.
25. Has donated > 450 mL blood or blood products within 30 days of study drug infusion.
26. Has any grade 2 or higher vital sign measurement (systolic or diastolic blood pressure, heart rate, or respiratory rate) at screening after 2 measurements.
27. Has a prolonged Fridericia-corrected QT interval at screening or check-in, defined as > 450 millisecond for males and > 460 millisecond for females, confirmed by repeat.
28. Is an employee or first-degree relative of Sponsor, contract research organization, or study site personnel.

5.3 Withdrawal of Subjects From the Study

5.3.1 Reasons for Withdrawal

Subjects can withdraw consent and discontinue from the study at any time, for any reason, without prejudice to further treatment.

Every reasonable attempt will be made to follow up with withdrawn subjects for safety. The reason for subject withdrawal will be documented.

The investigator can also withdraw a subject upon the request of the Sponsor or if the Sponsor terminates the study. Upon occurrence of a serious or intolerable AE, the investigator will confer with the Sponsor or designee. Any subject who is withdrawn from the study, will continue to be followed for safety evaluations.

5.3.2 Handling of Withdrawals

Subjects are free to withdraw from the study at any time upon request. Subject participation in the study may be stopped at any time at the discretion of the investigator or at the request of the Sponsor.

When a subject withdraws from the study, the reason(s) for withdrawal will be recorded by the investigator on the relevant page of the electronic case report form (eCRF). Whenever possible, any subject who withdraws from the study prematurely will undergo all end-of-study (EOS)/early termination (ET) assessments. Any subject who fails to return for final assessments will be contacted by the site with a minimum of 3 telephone call attempts, followed by a certified letter.

Lost to follow-up is defined by the inability to reach the subject after a minimum of 3 documented telephone calls, faxes, text messages, or emails as well as a lack of response by the subject to 1 registered mail letter. All attempts should be documented in the subject's medical records. The status of subjects who fail to complete final assessments will be documented in the eCRF.

5.3.3 Replacements

Subjects who withdraw, are withdrawn or terminated from this study, or are lost to follow-up after signing the informed consent form (ICF) but before the study drug infusion may be replaced. Subjects who receive the study drug infusion and subsequently withdraw, are terminated from the study, or are lost to follow-up will not be replaced.

6. STUDY TREATMENTS

6.1 Treatments Administered

Up to 56 subjects will receive either mRNA-1944 (0.1, 0.3, 0.45 [optional], 0.6, or 1.0 mg/kg) or placebo. Dose level cohorts will advance in a sequential manner after review of all safety data through 7 days after dosing of the previous dose level cohort by the SMC (Section 4.2.2). *All doses of mRNA-1944 and placebo will be administered IV as a single 100 mL infusion in 2 syringes using a syringe infusion pump over 1 to 3 hours at the study site. Dose administration will be done by appropriately trained clinic staff. Subjects will be observed for 48 hours as an inpatient following the administration of mRNA-1944 or placebo.*

Sequential dose escalation of mRNA-1944 is planned in 6 dose level cohorts (Section 4.2):

- Dose level cohort 1 (N = 8): 0.1 mg/kg mRNA-1944 or placebo (overall ratio = 3:1) on Day 1
- Dose level cohort 2 (N = 8): 0.3 mg/kg mRNA-1944 or placebo (overall ratio = 3:1) on Day 1
- Dose level cohort 3 (N = 8): 0.6 mg/kg mRNA-1944 or placebo (overall ratio = 3:1) (no dexamethasone premedication) on Day 1
- Dose level cohort 4 (N = 8) (optional dose level cohort): 0.45 mg/kg mRNA-1944 or placebo (overall ratio = 3:1) (dexamethasone premedication) on Day 1
- Dose level cohort 5 (N = 8): 0.6 mg/kg mRNA-1944 or placebo (overall ratio = 3:1) (dexamethasone premedication) on Day 1

- Dose level cohort 6 (N = 8): 1.0 mg/kg mRNA-1944 or placebo (overall ratio = 3:1) (dexamethasone premedication) on Day 1
- Dose level cohort 7 (N = 8): 0.3 mg/kg mRNA-1944 or placebo (overall ratio = 3:1) no dexamethasone premedication) on Day 1 and Day 8

The clinic will be appropriately staffed, trained on emergency resuscitation, and have stocked available rescue medications. Epinephrine and corticosteroids should be readily available for immediate use if necessary to counteract any immediate adverse reaction to the infusion.

6.1.1 *Dose Modifications*

Management of IRR should be based on the grade of the reaction and include slowing the rate, or temporary interruption, of the infusion (up to 4 hours). Mild to moderate IRRs (ie, CTCAE grades 1 and 2 and infusion reactions that do not involve symptoms of anaphylaxis) can usually be managed with slowing, or temporary interruption, of the infusion and symptom management with the administration of additional antihistamines, antipyretics, and/or corticosteroids. Per judgment of the investigator, after all symptoms have resolved, the infusion may be resumed at a reduced rate with the administration of additional premedications as indicated. (Section 4.1).

Reactions with any features of anaphylaxis (such as shortness of breath, urticaria, angioedema) or severe IRRs (CTCAE grade 3 or higher) require immediate and permanent discontinuation of the study drug infusion for the subject and the convening of an SMC meeting.

6.2 Investigational Products

The study drugs that will be used are as follows:

Product	Supplied Formulation
mRNA-1944 Injection	Frozen vials containing 2 mg/mL, with a nominal fill volume of 1.4 mL in each vial
Placebo	0.9% sodium chloride

The mRNA-1944 drug product consists of 2 mRNA drug substances in a lipid nanoparticle formulation intended for IV infusion. The mRNA-1944 drug product formulation includes 4 lipid excipients: IAL, a proprietary high-purity polyethylene glycol-2k-stearate monoester, and the commercially-available lipids cholesterol and DSPC. The mRNA-1944 drug product is formulated with 20 mM Tris buffer, 60 mM NaCl, 8% sucrose, 1.3% ethanol, and 1 mM diethylenetriaminepentaacetic acid at pH 7.5.

The placebo is 0.9% Sodium Chloride.

6.2.1 Study Drug Packaging and Storage

The Sponsor will provide the investigator and study site with adequate quantities of *mRNA-1944.* The placebo (0.9% Sodium Chloride) is commercially available. mRNA-1944 will have all the required labeling per regulations. mRNA-1944 will be supplied to the pharmacy in an unblinded manner.

The mRNA-1944 Injection will be supplied in glass vials at a 1.4-mL fill volume. The unblinded study site pharmacy personnel will prepare a single dose for each subject based on the dose level cohort and randomization assignment.

All doses will be prepared within 4 hours of dosing and administered as indicated in Section 6.1. A pharmacy manual will be available, and training provided to ensure pharmacy staff can comply with all study drug storage, preparation, and drug accountability procedures.

mRNA-1944 must be stored in a secure area with limited access (unblinded pharmacy staff only), protected from moisture and light, and be stored at –20°C ± 5°C. The freezer should have an automated temperature recording and alert system. There must be an available back up freezer. The freezers must be connected to a back-up generator. The pharmacy must have in place a 24-hour alert system that allows for rapid response in case of freezer malfunctioning. In addition, drug accountability study personnel (eg, the unblinded pharmacy staff) are required to keep a temperature log to establish a record of compliance with these storage conditions. The placebo will be stored according to the instructions on the product label and must also comply with storage in a restricted access area. Only drug accountability personnel should have access to the products used in this study.

The study site is responsible for reporting any mRNA-1944 that was not temperature controlled during shipment or during storage to the unblinded site (pharmacy) monitor. Such mRNA-1944 will be quarantined pending submission of the temperature excursion report in accordance with a predefined process.

6.2.2 Study Drug Accountability

It is the investigator’s responsibility to ensure that the unblinded pharmacy personnel maintain accurate records of receipt of all mRNA-1944 and placebo including dates of receipt. In addition, accurate records will be kept regarding when and how much mRNA-1944 or placebo is dispensed and used by each subject in the study (including documentation of each dilutional record used to prepare mRNA-1944 or placebo injection). Reasons for departure from the expected dispensing regimen must also be recorded. At the completion of the study, and to satisfy regulatory requirements regarding drug accountability, mRNA-1944 will be reconciled or returned to the Sponsor according to applicable regulations or destroyed.

6.3 Method of Assigning Subjects to Treatment Groups

Within each dose level cohort, 6 subjects will receive mRNA-1944 and 2 subjects will receive placebo. Randomization numbers will be assigned in a sequential, ascending fashion and sealed in an envelope.

For each dose level cohorts 1 through 6, a sentinel group of 3 subjects will be enrolled, and all will receive mRNA-1944, with a staggered minimum 7-day interval between each subject prior to treating the remaining subjects within a dose level cohort. For dose level cohort 7, a sentinel group of 3 subjects will be enrolled; all will be administered two IV infusions of 0.3 mg/kg mRNA-1944, one infusion on Day 1 and another subsequent infusion of 0.3 mg/kg mRNA-1944 on Day 8. *PPD* will generate the randomization schedule. The 5 remaining subjects in each dose level cohort (dose level cohorts 1 through 7) will be assigned a randomization number and randomly assigned to mRNA-1944 (0.1, 0.3, 0.45 [optional], 0.6, and 1.0 mg/kg) or placebo in a 3:2 ratio (active:placebo) for an overall ratio of 3:1 according to the randomization schedule.

6.4 Blinding

6.4.1 Blinding Procedures

This is an investigator-blinded study. The investigator, study subjects, site monitors, and study site personnel will be blinded to the study drug administered, with the following exceptions:

- The 3 sentinel subjects in each dose level cohort will receive mRNA-1944; the remaining 5 subjects within each dose level cohort will receive study drug (mRNA-1944 or placebo) in a blinded fashion.
- Unblinded pharmacy personnel (of limited number) will perform accountability procedures and prepare mRNA-1944 or placebo for all subjects. The unblinded pharmacy personnel will have no other study functions other than study drug management, documentation, accountability, and preparation. They will not be involved in subject evaluations and will not reveal the study drug identity to either the subject or study site personnel involved in the conduct of the study, except in the case of an emergency.
- Appropriately trained blinded study site staff will administer (via syringe infusion pump) mRNA-1944 or placebo to all subjects.
- An unblinded study monitor, not involved in other aspects of monitoring, will be assigned as the drug accountability monitor. They will have responsibilities to ensure the study site is following all proper drug accountability, preparation, and procedures.
- An unblinded clinical team manager will oversee the clinical management of the study.

- An unblinded team will provide the SMC an analysis of safety data (laboratory test results, AEs, prior and concomitant medication, ECGs [and cardiac enzymes when obtained per protocol], and vital signs) after the completion of each dose level cohort.

The treatment assignment will be concealed by having the unblinded pharmacy personnel prepare the study drug in a secure location that is not accessible to other study personnel. Access to the randomization code will be strictly controlled at the pharmacy.

6.4.2 Breaking the Blind

A subject or subjects may be unblinded in the event of an SAE or other event, or if there is a medical emergency requiring the identity of the study drug to be known to properly treat a subject. If a subject becomes seriously ill or pregnant during the study, the blind will be broken only if knowledge of the administered study drug will affect that subject's treatment options. In the event of a medical emergency requiring identification of the study drug administered to an individual subject, the investigator will make every attempt to contact the medical monitor to explain the need for opening the code within 24 hours of opening the code. The investigator will be responsible for documenting the time, date, reason for the code break, and the names of the personnel involved.

6.5 Treatment Compliance

The dose of the study drug will be administered at the study site under direct observation of clinic personnel. The date and start and stop time of study drug administration and any dose interruptions will be recorded on the appropriate page of the eCRF. Clinic personnel will confirm that the subject has received the entire dose of the study drug.

The study site is responsible for ensuring subjects comply with the study windows allowed. Should a subject miss a visit, every effort will be made to contact the subject and achieve a visit within the defined visit window outlined in the schedule of events (SOE) (Section 1).

6.6 Prior and Concomitant Medications

Restrictions for prior and concomitant medications and therapies are provided in Section 6.6.1 and Section 6.6.2. Prior and concomitant medications and therapies will be coded using the latest version of the World Health Organization Drug Dictionary (WHODrug).

6.6.1 Prior Medications

Information regarding prior medications (including any prescription or over-the-counter medications, vaccines, or blood products) taken by the subject within the 30 days before signing the ICF (or as designated in the inclusion/exclusion requirements, Section 5.1 and Section 5.2, respectively) will be recorded in the subject's eCRF.

6.6.2 Concomitant Medications

On Day 1, all subjects in dose level cohorts 1 and 2, and sentinel subjects in dose level cohort 3 will be premedicated with loratadine (10 mg, oral) and ranitidine (150 mg, oral) approximately 90 minutes prior to the planned start of study drug infusion; all subjects in the expansion group of dose level cohort 3 will be premedicated with loratadine (10 mg, oral), ranitidine (150 mg, oral), and acetaminophen (650 mg, oral) approximately 90 minutes prior to the planned start of study drug infusion; all subjects in dose level cohorts 5 and 6 (and the optional dose level cohort 4) will be premedicated with dexamethasone (10 mg, IV), diphenhydramine (50 mg, oral), and famotidine (20 mg, oral) approximately 90 minutes prior to the planned start of study drug infusion; and all subjects in dose level cohort 7 will be premedicated with diphenhydramine (50 mg, oral) and famotidine (20 mg, oral) approximately 90 minutes prior to the planned start of study drug infusion. Because 1 subject in the 0.6 mg/kg dose level cohort (after receiving the oral premedication regimen) developed a persistent grade 2 or higher IRR all subsequent subjects in the study who receive >0.3 mg/kg as a single dose will be administered dexamethasone (10 mg, IV), in addition to the above oral regimen, as premedication prior to study drug infusion.

Any concomitant medication deemed necessary for the welfare of the subject during the study may be given at the discretion of the investigator. If a concomitant medication is taken, except for those specified in the protocol, a joint decision will be made by the investigator and the Sponsor to continue or discontinue the subject based on the time the medication was administered, its pharmacology and PK, and whether the use of the medication will compromise the safety of the subject or the interpretation of the data. The investigator is responsible for ensuring that details regarding the concomitant medication are adequately recorded in the eCRF.

7. STUDY PROCEDURES

Before undergoing any study procedures, all potential subjects will sign an ICF as outlined in Section 10.2.2.3. Subjects will undergo study procedures at the time points specified in the SOE (Section 1).

At any time during the study a subject can be seen for an unscheduled visit. This may be prompted by abnormal laboratory test results, or new or ongoing AEs. The study site also has the discretion to make reminder phone calls or text messages to inform the subject regarding visits, request further laboratory assessments, or follow-up on ongoing or outstanding issues.

7.1 Pharmacokinetic Assessments and Endpoints

In all dose level cohorts, blood samples will be collected at the prespecified times outlined in the SOE (Section 1) to determine the serum concentrations of mRNA encoding for the CHIKV24 IgG and plasma concentrations of IAL.

The following serum and plasma PK parameters, where possible, will be calculated as endpoints for mRNA encoding for the CHIKV24 IgG and IAL respectively, using the actual sampling times relative to the start of infusion rather than the scheduled sampling times:

- Area under the concentration versus time curve (AUC) from time 0 to the last measurable concentration (AUC_{last})
- AUC from time 0 extrapolated to infinity (AUC_{inf})
- Maximum observed serum concentration (C_{max})
- Time to maximum observed serum concentration (t_{max})
- Terminal elimination half-life ($t_{1/2}$)
- Apparent clearance (CL)
- Volume of distribution at steady state (V_{ss})

7.1.1 Pharmacokinetic Sample Collection

Details for the collection, processing, storage, and shipping of PK samples will be provided to the study site separately.

7.2 **Pharmacodynamic** ***assessments and endpoints***

In all dose level cohorts, blood samples will be collected at the prespecified times outlined in the SOE (Section 1) to determine the serum concentrations of CHIKV24 IgG.

The following baseline corrected serum PD parameters will be calculated, where possible, for CHIKV24 IgG concentration using the actual sampling times relative to the start of infusion:

- Maximum observed effect (E_{max})
- Time to maximum observed effect (TE_{max})
- Terminal elimination half-life ($t_{1/2}$)
- Area under the effect curve (AUEC) from time 0 to the last measurable concentration ($AUEC_{last}$)
- Area under the effect curve from time 0 extrapolated to infinity ($AUEC_{inf}$)

7.2.1 Pharmacodynamic Sample Collection

Details for the collection, processing, storage, and shipping of PD samples will be provided to the study site separately.

7.3 Exploratory Assessments and Endpoints

In all dose level cohorts, blood samples will be collected at prespecified times outlined in the SOE (Section 1) to evaluate for the formation of anti-PEG, anti-CHIKV24 IgG titers, the assessment of in vitro serum neutralizing antibody titer, and the complement and acute phase reactants.

The exploratory endpoints include the following:

- Proportion of subjects with baseline-corrected increase in post-treatment anti-PEG antibodies
- Proportion of subjects positive for post-treatment anti-CHIKV24 IgG antibodies
- To evaluate the impact of several baseline characteristics on the PK/PD of mRNA-1944
- In vitro serum neutralizing antibody titer against a clinically relevant strain of CHIKV
- To evaluate complement and acute phase reactant parameters

7.4 Safety Assessments and Endpoints

Safety and tolerability will be assessed by the following endpoints: monitoring and recording of AEs (including SAEs, IRRs, and AEs of special interest [AESI]), prior and concomitant medication, clinical laboratory test results (hematology, coagulation, serum chemistry including liver enzymes, and urinalysis), vital sign measurements (systolic and diastolic blood pressure, heart rate, respiratory rate, and body temperature), ECG results (and cardiac enzymes when obtained per protocol), and physical examination findings.

7.4.1 Adverse Events

Adverse events will be assessed from the time of informed consent until EOS/ET and should be followed until they resolve, stabilize, or judged by the investigator to be not clinically significant.

The investigator is responsible for ensuring that all AEs and SAEs are recorded in the eCRF and reported to the Sponsor, regardless of their relationship to study drug or clinical significance. If there is any doubt as to whether a clinical observation is an AE, the event should be reported.

7.4.1.1 Adverse Event Definitions

An AE is defined as any untoward medical occurrence in a subject administered a study drug and which does not necessarily have a causal relationship with receipt of the study drug. An AE can therefore be any unfavorable and unintended sign (including an abnormal laboratory finding), symptom, or disease temporally associated with the use of an investigational study product/procedure(s). Subjects will be instructed to contact the investigator at any time after study drug infusion if any symptoms develop.

A treatment-emergent AE is defined as any event not present before exposure to study drug or any event already present that worsens in intensity or frequency after exposure.

An adverse reaction is any AE for which there is a reasonable possibility that the study drug caused the AE, ie, there is a reasonable possibility that there is a causal association between study treatment and the occurrence of the AE.

An IRR or suspected IRR is any AE related to or suspected of being related to the infusion of the study drug.

Adverse events leading to study withdrawal or dose modification will also be evaluated by the investigator.

7.4.1.2 Serious Adverse Event Definitions

An AE is considered “serious” (SAE) if, in the view of either the investigator or Sponsor, it results in any of the following outcomes:

- Results in death
- Is life-threatening

Note, the term “life-threatening” in the definition of “serious” refers to an event in which the subject was at risk of death at the time of the event; it does not refer to an event which hypothetically might have caused death if it were more severe.

- Requires inpatient hospitalization or prolongation of existing hospitalization
- Results in persistent or significant disability/incapacity
- Is a congenital anomaly/birth defect
- Is a medically important event

Important medical events are events that may not result in death, be life threatening, or require hospitalization but may be considered serious when, based upon appropriate medical judgment, they may jeopardize the subject and may require medical or surgical intervention to prevent 1 of the outcomes listed above in this definition. Examples of such medical events include allergic bronchospasm that requires intensive treatment in an emergency room or at home, blood dyscrasias or convulsions that do not result in inpatient hospitalization, or the development of drug dependency or drug abuse.

7.4.1.3 Adverse Events of Special Interest

Certain AESIs are evaluated after the administration of immunostimulatory agents. All subjects enrolled in the study will be monitored for AESIs from enrollment through the EOS/ET visit.

Adverse events of special interest include: hypersensitivity (including signs and symptoms of generalized rash, generalized pruritus, generalized erythema, cough, dyspnea, respiratory distress, acute bronchospasm, hypotension, and chest discomfort), anaphylactic reaction, acute allergic reaction, angioedema, and allergic urticaria. Adverse events of special interest also include gastrointestinal symptoms (nausea, diarrhea, abdominal pain, vomiting) and/or grade 2 or higher liver function test elevation.

A diagnosis of an AESI will be reported to the Sponsor in an expedited manner similar to that for an SAE. The AESI diagnosis, as well as any medications taken to treat the condition, will be recorded in the subject's eCRF.

7.4.1.4 Pregnancy

The investigator is required to inform the Sponsor (within 24 hours of investigator awareness) about any case of pregnancy occurring during the study (for female subjects as well as for partners of male subjects). The pregnancy must be followed until an outcome has been reached and report whatever outcome to the Sponsor.

Pregnancy report forms will be distributed to the study site to be used for this purpose.

The investigator is required to inform the Sponsor (within 24 hours of awareness) about any case of pregnancy resulting in an abnormal outcome (miscarriage, newborn with congenital abnormality, and/or stillbirth) according to the procedures described for SAEs.

7.4.1.5 Eliciting and Documenting Adverse Events

The investigator is responsible for ensuring that all AEs and SAEs are recorded in the eCRF and reported to ModernaTX, Inc.

Adverse events will be assessed from the time informed consent is provided. All AEs will be recorded through 28 days after study drug infusion (after the second infusion for dose level cohort 7), all medically attended AEs will be recorded for 3 months after study drug infusion, and all SAEs and AESIs (Section 7.4.1.3) will be recorded through Week 52. Any new SAE assessed as related to study drug infusion should be reported to the Sponsor, regardless of when the SAE occurs, throughout the duration of the study.

At every clinic visit or telephone contact, subjects will be asked a standard question to elicit any medically related changes in their well-being. Subjects will also be asked if they have been hospitalized, had any accidents, used any new medications, or changed concomitant medication regimens (both prescription and over-the-counter medications).

In addition to subject observations, data from clinical laboratory test results, physical examination findings, ECG results (and cardiac enzymes when obtained per protocol), vital sign measurements,

or other documents relevant to subject safety that indicate an AE will be documented on the AE page of the eCRF.

7.4.1.6 Reporting Adverse Events

All AEs reported or observed during the study will be recorded on the AE page of the eCRF. Information to be collected includes drug treatment and dose, type of event, time of onset, investigator-specified assessment of severity (toxicity grading) and relationship to study drug, time of resolution of the event, seriousness, any required treatment or evaluations, and outcome. Any AEs resulting from concurrent illnesses, reactions to concurrent illnesses, reactions to concurrent medications, or progression of disease states must also be reported. All AEs will be followed until they are resolved, stable, or judged by the investigator to be not clinically significant. If any related, nonserious AE, or any SAE regardless of relationship is unresolved at study completion, a clinical assessment will be made by the investigator and medical monitor to determine whether continued follow-up of the AE is warranted. The Medical Dictionary of Regulatory Activities (MedDRA) will be used to code all AEs.

Any medical condition that is present at the time that the subject is screened but does not deteriorate should not be reported as an AE. However, if it deteriorates at any time during the study, it should be recorded as an AE.

Any AE that is considered serious by the investigator or which meets SAE criteria (Section 7.4.1.2) must be reported to the Sponsor immediately (after the investigator has confirmed the occurrence of the SAE). The investigator will assess whether there is a reasonable possibility that the study drug caused the SAE. The Sponsor will be responsible for notifying the relevant regulatory authorities of any SAE in accordance with Title 21 of the Code of Federal Regulations Part 312.32. The investigator is responsible for notifying the institutional review board or independent human research ethics committee directly.

7.4.1.7 Assessment of Severity

The severity (or intensity) of an AE refers to the extent to which it affects the subject's daily activities and will be classified according to the toxicity grading scale (Section 10.4) as mild (grade 1), moderate (grade 2), severe (grade 3), and potentially life threatening (grade 4) using the following criteria:

- Mild (grade 1): These events do not interfere with the subject's daily activities.
- Moderate (grade 2): These events cause some interference with the subject's daily activities but do not require medical intervention.

- Severe (grade 3): These events prevent the subject's daily activities and require medical intervention.
- Life threatening (grade 4): These events require an emergency room visit or hospitalization.

Changes in the severity of an AE should be documented to allow the duration of the event at each level of intensity to be assessed. An AE characterized as intermittent does not require documentation of the onset and duration of each episode. The CTCAE v5 grading scale will be used to assess all AEs and laboratory abnormalities for the purposes of determining whether a pause criterion has been met. Vital signs will be graded using the toxicity grading scale for healthy adult and adolescent volunteers enrolled in preventative vaccine clinical trials; tables for clinical abnormalities (DHHS 2007).

7.4.1.8 Assessment of Causality

The investigator's assessment of an AE's relationship to study drug is part of the documentation process but is not a factor in determining what is or is not reported in the study.

The investigator will assess causality (ie, whether there is a reasonable possibility that the study drug caused the event) for all AEs, AESIs, and SAEs. The relationship will be characterized using the following classification:

- Not related: There is not a reasonable possibility of a causal association between the event and the administration of the Sponsor's investigational product. Examples: it could be that the subject did not receive the investigational product, OR the temporal sequence of the AE onset relative to administration of the investigational product is not reasonable, OR the AE is more likely explained by another cause than the investigational product.
- Related: There is a reasonable possibility of a causal association between the event and the administration of the Sponsor's investigational product. Examples: there is evidence of exposure to the investigational drug product AND the temporal sequence of the AE onset relative to the administration of the investigational product is reasonable, OR the AE is more likely explained by the investigational product than by another cause.

7.4.1.9 Follow-Up of Adverse Events

All AEs must be reported in detail on the appropriate page of the eCRF and followed until the AE is resolved, stable, or judged by the investigator to be not clinically significant. The investigator may request an unscheduled visit at any time, if warranted. If any related, nonserious AE, or any SAE regardless of relationship is unresolved at study completion, a clinical assessment will be made by the investigator and medical monitor whether continued follow-up of the AE is warranted.

7.4.2 Infusion-Related Reaction Assessments

The infusion site will be examined by the investigator or designee for pain, tenderness, erythema/redness, and induration/swelling as indicated in the SOE (Section 1). Infusion site reactions will be assessed according to the CTCAE v5 toxicity grading scale (Section 10.4), will be recorded as AEs, and should be followed until resolution. Suspected allergic (hypersensitivity) reactions and anaphylaxis will also be assessed according to the clinical diagnostic criteria outlined by the National Institute of Allergy and Infectious Diseases and CTCAE v5 (Section 10.5).

7.4.3 Clinical Laboratory Testing

Clinical laboratory testing will be performed at the time points indicated in the SOE (Section 1) at the study site or central laboratory. Fasting is not required before the collection of laboratory samples.

Repeat clinical laboratory tests may be performed at the discretion of the investigator, if necessary, to evaluate inclusion and exclusion criteria or clinical laboratory abnormalities. The clinical laboratory will provide the reference ranges for all clinical laboratory parameters. The results will be toxicity graded using the tables in Section 10.4.

The following clinical laboratory assessments will be performed:

Hematology:	Hematocrit, hemoglobin, mean corpuscular hemoglobin, mean corpuscular hemoglobin concentration, mean corpuscular volume, platelet count, red blood cell count, and total and differential leukocyte count
Serum Chemistry:	Alanine aminotransferase, alkaline phosphatase, aspartate aminotransferase, bilirubin (total and direct), blood urea nitrogen, creatinine, random glucose, potassium, sodium, total protein, albumin, and calcium<br>Females subjects only: β-human chorionic gonadotropin (childbearing potential) and follicle-stimulating hormone (post-menopausal)
Coagulation:	Prothrombin time, international normalized ratio, and partial thromboplastin time
Urinalysis:	pH, protein, glucose, ketone, bilirubin, urobilinogen, blood, nitrite, leukocytes, and specific gravity

Complement:	Serum completement membrane attack complex
Acute phase reactants:	C-reactive protein, interleukin-6, and interferon-inducible protein-10

A pregnancy test (β-human chorionic gonadotropin) will be performed on all female subjects who have reproductive potential at screening and at check-in (serum). A follicle-stimulating hormone test will be performed at screening, as necessary, to confirm postmenopausal status in female subjects, if not documented in the subject's medical records.

Human immunodeficiency virus (type 1 and 2) antibody, hepatitis B surface antigen, and hepatitis C virus antibody will be assessed at screening.

A urine screen for drugs of abuse will be performed by the local laboratory at screening and on Day –1 for alcohol, opiates, cocaine, phencyclidine, amphetamines, benzodiazepines, and methadone.

If safety laboratory testing results in a grade 2 or higher toxicity, repeat testing must occur within the next 10 days; this may include an unscheduled visit. If the subject's laboratory value does not return to baseline, then periodic testing may be needed until the abnormality is considered associated with a new stable AE or determined to be not clinically significant by the investigator.

Those values that fall within the normal range of the laboratory will automatically be classified as normal. All values that have a toxicity of grade 1 or higher will also be evaluated by the investigator and classified as "abnormal clinically significant", or "abnormal not clinically significant". The investigators should use their clinical judgment when considering the clinical significance of any abnormal laboratory findings. All laboratory test values with a toxicity of grade 3 or higher will be entered as AEs. Any additional laboratory test value that is determined to be clinically significant will also be recorded as an AE, if it is considered the primary diagnosis. In such instances, the abnormal value and grade will be documented on the AE page of the eCRF. The investigator will continue to monitor the subject with additional assessments until the values reach the reference range or the values at screening or until the investigator determines that follow-up is no longer medically necessary. The only exception to this rule is a laboratory test value that is associated with an identified ongoing AE that is considered the classifying AE and is recorded as such.

7.4.4 Vital Sign Measurements

Vital sign measurements will include systolic and diastolic blood pressures, heart rate, respiratory rate, and body temperature. Vital sign measurements will be collected after the subject has been

resting in a semi-recumbent position for at least 5 minutes. Vital signs will be measured at the time points indicated in the SOE (Section 1).

If any of the vital sign measurements meet the toxicity grading criteria for a clinical abnormality (Section 10.4) of Grade 3 or higher, the abnormal value and grade will be documented on the AE page of the eCRF (unless there is another known cause of the abnormality that would result in an AE classification). The investigator will continue to monitor the subject with additional assessments until the value reaches the reference range or the value at screening or is considered stable or until the investigator determines that follow-up is no longer medically necessary.

7.4.5 12-Lead Electrocardiograms

Safety 12-lead ECGs will be obtained after the subject has rested in the supine position for at least 5 minutes, at the time points indicated in the SOE (Section 1). Electrocardiogram assessments will include comments on whether the tracings are normal or abnormal, the rhythm, the presence of arrhythmia or conduction defects, morphology, any evidence of myocardial infarction, or ST-segment, T-Wave, and U-Wave abnormalities. In addition, measurements of the following intervals will be measured and reported: RR interval, PR interval, QRS width, and Fridericia-corrected QT interval.

The investigator will determine whether any of the 12-lead ECG results are clinically significant or not clinically significant. Clinical significance is defined as any variation in results that has medical relevance and may result in an alteration in medical care (eg, active observation, diagnostic measures, or therapeutic measures). If a clinically significant change from screening is noted, the clinically significant value and reason for clinical significance will be documented on the AE page of the subject's eCRF. If a clinically significant change from screening is noted on the ECG, blood samples for the assessment of cardiac enzymes (troponin I and creatine kinase-MB) will be drawn within 1 hour and at 6 and 24 hours after the noted abnormal ECG. The investigator will continue to monitor the subject with additional assessments until either the values have reached reference range or the values at screening or until the investigator determines that follow-up is no longer medically necessary.

7.4.6 Physical Examinations

A full physical examination will be performed at screening and on Day –1, and a symptom-directed (targeted) physical examination will be performed at all other scheduled time points indicated in the SOE (Section 1). The full examination will include assessments of skin, head, ears, eyes, nose, throat, neck, thyroid, lungs, heart, cardiovascular system, abdomen, lymph nodes, and musculoskeletal system/extremities. Interim physical examinations will be performed at the discretion of the investigator, if necessary, to evaluate AEs or clinical laboratory abnormalities.

Height will be measured at screening only. Weight will be measured at screening as well as at check-in, for dose calculation.

8. STATISTICAL ANALYSIS PLANS, DATA ANALYSIS, AND STATISTICAL METHODS

This section outlines the core elements of the planned statistical summaries and analyses for the data collected in the study. The detailed methodology for the summary and statistical analyses of the data collected in this study will be documented in the statistical analysis plan.

8.1 Sample Size Calculations

Approximately 56 subjects (8 per dose level cohort) are planned to be enrolled; 6 subjects will receive mRNA-1944 and 2 subjects will receive placebo in each dose level cohort. Among the 6 subjects who receive mRNA-1944, 3 are the sentinel subjects. The remaining 5 subjects within each dose level cohort will be randomly assigned in a 3:2 ratio to receive mRNA-1944 or placebo. Formal sample size calculations were not performed, and no formal hypotheses are being tested.

8.2 Analysis Sets

The Safety Population will include all subjects who enrolled and received any dose infusion of mRNA-1944 or placebo. The Safety Population will be used in the safety and exploratory analyses.

The PK analysis dataset will include subjects in the Safety Population who have evaluable mRNA encoding for the CHIKV24 IgG and IAL concentrations and do not have any major protocol deviations impacting the PK assessments.

The PD analysis dataset will include subjects in the Safety Population who have evaluable CHIKV24 IgG concentrations and do not have any major protocol deviations impacting the PD assessments.

All data collected will be presented in data listings. Data from subjects excluded from an analysis population or dataset will be presented in the data listings but not included in the calculation of the respective summary statistics.

8.3 Statistical Analysis

For categorical variables, frequencies and percentages will be presented. Continuous variables will be summarized using descriptive statistics (number of subjects [n], arithmetic mean, median, SD, minimum, and maximum).

8.3.1 Pharmacokinetic Analyses

Serum mRNA encoding for the CHIKV24 IgG and plasma IAL concentrations will be listed and summarized descriptively by dose level (n, arithmetic mean, SD, coefficient of variation [CV], median, geometric mean, geometric CV, minimum, and maximum). Concentration versus time profiles for each subject will be presented graphically. The mean concentration versus scheduled time profiles will be presented graphically.

Pharmacokinetic parameters will be derived using noncompartmental methods with Phoenix® WinNonlin® (Certara USA Inc., Princeton, New Jersey) Version *6.4* or higher or SAS Version 9.3 or higher (SAS Institute Inc., Cary, North Carolina), and will be summarized by dose level using descriptive statistics (n, arithmetic mean, SD, CV, median, geometric mean, geometric CV, minimum, and maximum). For t_{max}, only n, median, minimum, and maximum will be presented.

Additionally, concentrations and PK parameters will be summarized by dose level using descriptive statistics for subjects with a negative anti-drug antibody response.

The statistical relationship between dose and PK parameters will be assessed overall and for subjects with a negative anti-drug antibody response.

8.3.2 Pharmacodynamic Analyses

CHIKV24 IgG concentrations will be listed and summarized descriptively by dose level (n, arithmetic mean, SD, CV, median, geometric mean, geometric CV, minimum, and maximum). CHIKV24 IgG absolute and baseline corrected concentration versus time profiles for each subject will be presented graphically. The mean serum CHIKV24 IgG absolute and baseline corrected concentration versus scheduled time profiles will be presented graphically.

Pharmacodynamic parameters derived from baseline corrected concentration using noncompartmental methods with Phoenix® WinNonlin® (Certara USA Inc., Princeton, New Jersey) Version *6.4* or higher or SAS Version 9.3 or higher (SAS Institute Inc., Cary, North Carolina) will be summarized by dose level using descriptive statistics (n, arithmetic mean, SD, CV, median, geometric mean, geometric CV, minimum, and maximum). For TE_{max}, only n, median, minimum, and maximum will be presented.

Additionally, serum concentrations and PD parameters will be summarized by dose level using descriptive statistics by anti-drug antibody response.

The statistical relationship between dose and CHIKV IgG baseline-corrected PD parameters will be assessed overall by anti-drug antibody response.

8.3.3 Exploratory Analysis

Exploratory endpoints will be listed, and summary statistics will be provided by dose level for each exploratory endpoint.

Additional analyses may be conducted to evaluate the effect of subject characteristics on the PK and PD of mRNA encoding for the CHIKV24 IgG and IAL and to evaluate complement and acute phase reactant parameters.

8.3.4 Safety Analyses

Baseline demographic and background variables will be summarized by dose level cohort for all subjects. The number of subjects who enroll in the study and the number and percentage of subjects who complete the study will be presented. Frequency and percentage of subjects who withdraw or discontinue from the study and the reasons for withdrawal or discontinuation will also be summarized. Height will be measured at screening only. Weight will be measured at screening as well as at check-in, for dose calculation. Height and weight measurements will be presented in the listings. Baseline height and weight measurements will be summarized together with the demographic data.

Prior and concomitant medication will be listed (with start and stop dates) for each subject and summarized by WHODrug classification.

Adverse events will be coded by preferred term and system organ class using the latest version of the MedDRA and summarized by treatment group and overall.

All AE data will be presented in a data listing.

Treatment-emergent AEs will be summarized by treatment group and overall, as well as by severity (toxicity grading) and relationship to study drug. Serious AEs, IRRs, AESIs, AEs leading to study withdrawal or dose modification, and deaths will also be presented in the data listings and summarized by treatment, dose, and overall. Data for subjects receiving placebo will be pooled across all dose level cohorts.

Actual values and changes from baseline for clinical laboratory test results, ECG results (and cardiac enzymes when obtained per protocol), and vital sign measurements will be summarized by dose level and treatment at each time point using descriptive statistics (number of subjects, mean, SD, median, minimum, and maximum). Clinical laboratory test results and vital sign measurements will be assessed by toxicity grading. All data will be presented in data listings.

8.4 Handling of Missing Data

Plasma and serum concentrations that are below the limit of quantification (BLQ) will be imputed as zero for descriptive statistics. Mean BLQ concentrations will be presented as BLQ, and the SD

and CV will be reported as not applicable. Missing concentrations will be excluded from the calculations.

For the PK analysis, BLQ values will be imputed as zero with the exception that a BLQ value between 2 quantifiable concentrations will be set as missing. Missing concentrations will be treated as missing from the PK parameter calculations. If consecutive BLQ concentrations are followed by quantifiable concentrations in the terminal phase, those concentrations after BLQ concentrations will be set as missing.

8.5 Periodic Pharmacokinetic/Pharmacodynamic Analyses

Periodic PK and PD analyses will be performed following completion of all subjects in each dose level cohort and will include data through at least 7 days after study drug infusion and the parameters listed in Sections 7.1 and 7.2. A final analysis will be performed at end of study following database lock.

8.6 Interim Analyses

Periodic PK and PD analyses may be performed following completion of all 7 dose level cohorts and will include the parameters listed in Section 7.1 and 7.2. All data used for interim analyses will be unblinded to subject level treatment assignments and the related parameter values used for interim analyses will be derived from Phoenix® WinNonlin® (Certara USA Inc., Princeton, New Jersey) Version *6.4* or higher based on nominal dosage and times.

The data used for interim safety analyses will include subject disposition, demographic and baseline characteristics, study drug administration (including infusion rate changes), laboratory test results, AEs, both infusion-related and non-infusion-related, prior and concomitant medication, ECGs (and cardiac enzymes when obtained per protocol), and vital signs.

9. REFERENCE LIST

Centers for Disease Control and Prevention, National Center for Emerging and Zoonotic Infectious Diseases, Division of Vector-Borne Disease. Chikungunya virus: geographic distribution [Internet]. Atlanta (GA); 2016 Apr 22 [cited 2016 Sep 09]. Available from: http://www.cdc.gov/chikungunya/geo/index.html.

Common Terminology Criteria for Adverse Events (CTCAE) Version 5, US Department of Health and Human Services. [Internet] Published 2017 Nov 27 [cited 2018 May 20]. Available from: https://ctep.cancer.gov/protocolDevelopment/electronic_applications/docs/CTCAE_v5_Quick_Reference_5x7.pdf.

Couderc T, Khandoudi N, Grandadam M, et al. Prophylaxis and therapy for chikungunya virus infection. J Infect Dis. 2009;200(4):516-23.

Department of Health and Human Services (DHHS), Food and Drug Administration, Center for Biologics Evaluation and Research (US). Guidance for industry: Toxicity grading scale for healthy adult and adolescent volunteers enrolled in preventative vaccine clinical trials. September 2007 [cited 2018 May 20] [10 screens]. Available from:

http://www.fda.gov/downloads/BiologicsBloodVaccines/GuidanceComplianceRegulatoryInformation/Guidances/Vaccines/ucm091977.pdf.

Kam YW, Lum FM, Teo TH, et al. Early neutralizing IgG response to chikungunya virus in infected patients targets a dominant linear epitope on the E2 glycoprotein. EMBO Mol Med. 2012;4(4):330-43.

Kam YW, Simarmata D, Chow A, et al. Early appearance of neutralizing immunoglobulin G3 antibodies is associated with chikungunya virus clearance and long-term clinical protection. J Infect Dis. 2012;205(7):1147-54.

Lanciotti RS, Kosoy OL, Laven JJ, et al. Chikungunya virus in US travelers returning from India, 2006. Emerg Infect Dis. 2007;13(5):764-7.

Pan American Health Organization (PAHO), Regional Office for the Americas of the World Health Organization. Number of reported cases of chikungunya fever in the Americas, by country or territory [Internet]. Washington, DC; 2017 Dec 22 [cited 2018 May 20]. Available from: http://www.paho.org/hq/index.php?option=com_docman&task=doc_view&Itemid=270&gid=43280&lang=en

Partidos CD, Weger J, Brewoo J, et al. Probing the attenuation and protective efficacy of a candidate chikungunya virus vaccine in mice with compromised interferon (IFN) signaling. Vaccine. 2011;29(16):3067-73.

Powers AM, Logue CH. Changing patterns of chikungunya virus: re-emergence of a zoonotic arbovirus. Journal of General Virology. 2007; 88: 2363-2377.

Schwartz O, Albert ML. Biology and pathogenesis of chikungunya virus. Nat Rev Microbiol. 2010;8(7):491-500.

Smith SA, Silva LA, Fox JM, et al. Isolation and characterization of broad and ultrapotent human monoclonal antibodies with therapeutic activity against chikungunya virus. Cell Host Microbe. 2015;18(1):86-95.

Weaver SC, Osorio JE, Livengood JA, et al. Chikungunya virus and prospects for a vaccine. Expert Rev Vaccines. 2012;11(9):1087-101.

World Health Organization. Chikungunya [Internet]. Geneva, Switzerland; 2017 Apr 12 [cited 2018 Jun 20]. Available from: http://www.who.int/mediacentre/factsheets/fs327/en/

10. APPENDICES

10.1 Appendix 1: List of Abbreviations

Abbreviation	Term
AE	adverse event
AESI	adverse event of special interest
Apo E	apolipoprotein E
AUC	area under the concentration versus time curve
AUC_{inf}	area under the concentration versus time curve from time 0 extrapolated to infinity
AUC_{last}	area under the concentration versus time curve from time 0 to the last measurable concentration
AUEC	area under the effect curve
$AUEC_{inf}$	area under the effect curve from time 0 extrapolated to infinity
$AUEC_{last}$	area under the effect curve from time 0 to the last measurable concentration
BLQ	below the limit of quantification
CHIKV	chikungunya virus
CL	apparent clearance
C_{max}	maximum observed serum concentration
CTCAE	Common Terminology Criteria for Adverse Events
CV	coefficient of variation
DSPC	1,2-distearoyl-sn-glycero-3-phosphocholine
DTPA	diethylenetriaminepentaacetic acid
ECG	electrocardiogram
eCRF	electronic case report form
E_{max}	maximum observed effect
EOI	end of infusion
EOS	end of study
ET	early termination
FIH	*first-in-human*
GLP	Good Laboratory Practice
GMP	Good Manufacturing Practices
ICF	informed consent form
ICH	International Council for Harmonisation
IgG	immunoglobulin G

Abbreviation	Term
IRR	infusion-related reaction
IST	internal safety team
IV	intravenous(ly)
MedDRA	Medical Dictionary for Regulatory Activities
mRNA	messenger RNA
NOAEL	no observed adverse effect level
PD	pharmacodynamic(s)
PEG	polyethylene glycol
PK	pharmacokinetic(s)
SAE	serious adverse event
SMC	safety monitoring committee
SOE	schedule of events
$t_{1/2}$	terminal elimination half-life
t_{max}	time to maximum observed serum concentration
TE_{max}	time to maximum observed effect
V_{ss}	volume of distribution at steady state

10.2 Appendix 2: Study Governance

10.2.1 Data Quality Assurance

All aspects of the study will be monitored for compliance with applicable government regulations with respect to current ICH guideline E6(R2): Good Clinical Practice and current standard operating procedures. Electronic CRFs and electronic data capture will be utilized. The electronic data capture system is validated and compliant with local regulations. Each person involved with the study will have an individual identification code and password that allows for record traceability. There may be an internal quality review audit of the data and additional reviews by the clinical monitor.

10.2.2 Investigator Obligations

The following administrative items are meant to guide the investigator in the conduct of the study and may be subject to change based on industry and government standard operating procedures, working practice documents, or guidelines. Changes will be reported to the Ethics Committee but will not result in protocol amendments.

10.2.2.1 Confidentiality

All laboratory specimens, evaluation forms, reports, and other records will be identified in a manner designed to maintain subject confidentiality. All records will be kept in a secure storage area with limited access. Clinical information will not be released without the written permission of the subject (or the subject's legal guardian), except as necessary for monitoring and auditing by the Sponsor, its designee, relevant regulatory authorities, or the Ethics Committee.

The investigator and all employees and coworkers involved with this study may not disclose or use for any purpose, other than performance of the study, any data, record, or other unpublished confidential information disclosed to those individuals for the purpose of the study. Prior written agreement from the Sponsor or its designee must be obtained for the disclosure of any said confidential information to other parties.

10.2.2.2 Institutional Review

Federal regulations and ICH guidelines require that approval be obtained from an Ethics Committee before the participation of human subjects in research studies. Before study onset, the protocol, ICF, advertisements to be used for the recruitment of study subjects, and any other written information regarding this study that is to be provided to the subject or the subject's legal guardian must be approved by the Ethics Committee. Documentation of all Ethics Committee approvals and of Ethics Committee compliance with the ICH harmonised tripartite guideline E6(R2): Good

Clinical Practice will be maintained by the site and will be available for review by the Sponsor or its designee.

All Ethics Committee approvals should be signed by the Ethics Committee chairman or designee and must identify the Ethics Committee name and address, the clinical protocol by title or protocol number or both, and the date approval or a favorable opinion was granted.

10.2.2.3 Subject Consent

Written informed consent in compliance with local regulatory authority requirements shall be obtained from each subject before he or she enters the study or before performing any unusual or nonroutine procedure that involves risk to the subject. If any institution-specific modifications to study-related procedures are proposed or made by the site, the consent should be reviewed by the Sponsor, its designee, or both before Ethics Committee submission. Once reviewed, the investigator will submit the ICF to the Ethics Committee for review and approval before the start of the study. If the ICF is revised during the course of the study, all active participating subjects must sign the revised form.

Before recruitment and enrollment, each prospective subject or his/her legal guardian will be given a full explanation of the study and will be allowed to read the approved ICF. Once the investigator is assured that the subject/legal guardian understands the implications of participating in the study, the subject/legal guardian will be asked to give his or her consent to participate in the study by signing the ICF. A copy of the ICF will be provided to the subject/legal guardian.

10.2.2.4 Study Reporting Requirements

By participating in this study, the investigator agrees to submit reports of SAEs according to the time line and method outlined in this protocol. In addition, the investigator agrees to submit annual reports to his or her Ethics Committee as appropriate.

10.2.2.5 Financial Disclosure and Obligations

The investigator is required to provide financial disclosure information to allow the Sponsor to submit the complete and accurate certification or disclosure statements required under local regulations. In addition, the investigator must provide to the Sponsor a commitment to promptly update this information if any relevant changes occur during the course of the investigation and for 1 year following the completion of the study.

Neither the Sponsor, PPD, nor the study site is financially responsible for further testing or treatment of any medical condition that may be detected during the screening process. In addition, in the absence of specific arrangements, neither the Sponsor, PPD, nor the study site is financially responsible for further treatment of the disease under study.

10.2.2.6 Investigator Documentation

Prior to beginning the study, the investigator will be asked to comply with ICH E6(R2) Section 8.2 and local regulations by providing essential documents, including but not limited to, the following:

- Ethics Committee approval.
- An original investigator-signed investigator agreement page of the protocol.
- Current licensure must be noted on the curriculum vitae. Curriculum vitae will be signed and dated by the principal investigators and subinvestigators at study start-up, to indicate that they are accurate and current.
- Financial disclosure information to allow the Sponsor to submit the complete and accurate certification or disclosure statements required under local regulation. In addition, the investigators must provide to the Sponsor a commitment to promptly update this information if any relevant changes occur during the course of the investigation and for 1 year after the completion of the study.
- An Ethics Committee -approved ICF, samples of site advertisements for recruitment for this study, and any other written information about this study that is to be provided to the subject or legal guardians.
- Laboratory certifications and reference ranges for any local laboratories used by the site, in accordance with local regulations.

10.2.2.7 Study Conduct

The investigator agrees to perform all aspects of this study in accordance with the ethical principles that have their origin in the Declaration of Helsinki, ICH E6(R2): Good Clinical Practice; the protocol; and all national, state, and local laws or regulations.

10.2.2.8 Case Report Forms and Source Documents

Site personnel will maintain source documentation, enter subject data into the eCRF as accurately as possible, and rapidly respond to any reported discrepancies.

Electronic CRFs and electronic data capture system is validated and compliant with regulations. Each person involved with the study will have an individual identification code and password that allow for record traceability. Thus, the system and any subsequent investigative reviews can identify coordinators, investigators, and individuals who have entered or modified records, as well as the time and date of any modifications. There may be an internal quality review audit of the data and additional reviews by the clinical monitor.

Each eCRF is presented in an electronic format to allow data entry by site personnel, who can add and edit data, add new subjects, identify and resolve discrepancies, and view records. This system provides immediate direct data transfer to the database, as well as immediate detection of discrepancies, thus enabling site coordinators to resolve and manage discrepancies in a timely manner.

10.2.2.9 Adherence to Protocol

The investigator agrees to conduct the study as outlined in this protocol, in accordance with ICH E6(R2) and all applicable guidelines and regulations.

10.2.2.10 Reporting Adverse Events

By participating in this study, the investigator agrees to submit reports of SAEs according to the timeline and method outlined in this protocol. In addition, the investigator agrees to submit annual reports to his or her Ethics Committee as appropriate. The investigator also agrees to provide the Sponsor with an adequate report, if applicable, shortly after completion of the investigator's participation in the study.

10.2.2.11 Investigator's Final Report

Upon completion of the study, the investigator, where applicable, should inform the institution; the investigator/institution should provide the Ethics Committee with a summary of the study's outcome and the Sponsor and regulatory authorities with any reports required.

10.2.2.12 Records Retention

Essential documents should be retained for at least 2 years after the last approval of a marketing application in an ICH region and until there are no pending or contemplated marketing applications in an ICH region or until at least 2 years have elapsed since the formal discontinuation of clinical development of the study drug. These documents should be retained for a longer period, however, if required by applicable regulatory requirements or by an agreement with the Sponsor. The Sponsor is responsible for informing the investigator/institution when these documents no longer need to be retained.

10.2.2.13 Publications

After completion of the study, the data may be considered for reporting at a scientific meeting or for publication in a scientific journal. In these cases, the Sponsor will be responsible for these activities and will work with the investigators to determine how the manuscript is written and edited, the number and order of authors, the publication to which it will be submitted, and any other related issues. The Sponsor has final approval authority over all such issues.

The data are the property of the Sponsor and cannot be published without their prior authorization, but data and any publication thereof will not be unduly withheld.

10.2.3 Study Management

10.2.3.1 Monitoring

10.2.3.1.1 Monitoring of the Study

The clinical monitor is obligated to follow the study closely. In doing so, the monitor will visit the investigator and study site at periodic intervals in addition to maintaining necessary telephone and email contact. The monitor will maintain current personal knowledge of the study through observation, review of study records and source documentation, and discussion of the conduct of the study with the investigator and staff.

All aspects of the study will be carefully monitored by the Sponsor or its designee for compliance with applicable government regulations with respect to current ICH E6(R2) guidelines and standard operating procedures. One monitor will be partially blinded to treatment assignments and 1 monitor will be unblinded to treatment assignment.

10.2.3.1.2 Inspection of Records

The investigator and institution involved in the study will permit study-related monitoring, audits, Ethics Committee review, and regulatory inspections by providing direct access to all study records. In the event of an audit, the investigator agrees to allow the Sponsor, their representatives, or the regulatory authority access to all study records.

The investigator should promptly notify the Sponsor and study site of any audits scheduled by any regulatory authorities and promptly forward copies of any audit reports received to the Sponsor.

10.2.3.2 Management of Protocol Amendments and Deviations

10.2.3.2.1 Modification of the Protocol

This is a Phase 1 assessment of the safety, tolerability, PK, and PD of mRNA-1944 and SM 86 in humans. This protocol is written with some flexibility to accommodate the inherent dynamic nature and dose finding of Phase 1 clinical studies. Modifications to the dose, dosing regimen, and/or clinical or laboratory procedures currently outlined below may be required to achieve the scientific goals of the study objectives and/or to ensure appropriate safety monitoring of the study subjects.

As such, some alterations from the currently outlined dose and/or dosing regimen by the Sponsor may be permitted based on newly available data, but the maximum dose may not exceed that currently outlined in the protocol and no subject shall receive more than 1 dose of the study drug

infusion. The total blood volume withdrawn from any single subject will not exceed the maximum allowable volume during his or her participation in the entire study.

The timing of procedures for assessment of safety procedures (eg, vital signs, ECG [and cardiac enzymes when obtained per protocol], safety laboratory tests) currently outlined in the protocol may be modified during the study based on newly available safety and tolerability data. Additional laboratory safety tests may be added to blood samples previously collected to obtain additional safety information (eg, adding creatinine kinase to a serum chemistry panel that was already collected). These changes will not increase the number of study procedures for a given subject during his or her participation in the entire study.

It is understood that the current study may employ some or none of the alterations described above. Any alteration made to this protocol to meet the study objectives must be detailed by the Sponsor in a letter to the Trial File and forwarded to the investigator for retention. The letter may be forwarded to the Ethics Committee at the discretion of the investigator.

10.2.3.2.2 Protocol Deviations

The investigator or designee must document and explain in the subject's source documentation any deviation from the approved protocol. The investigator may implement a deviation from, or a change to, the protocol to eliminate an immediate hazard to study subjects without prior Ethics Committee approval. As soon as possible after such an occurrence, the implemented deviation or change, the reasons for it, and any proposed protocol amendments should be submitted to the Ethics Committee for review and approval, to the Sponsor for agreement, and to the regulatory authorities, if required.

A protocol deviation is any change, divergence, or departure from the study design or procedures defined in the protocol. An important protocol deviation (sometimes referred to as a protocol violation or a major protocol deviation) is a subset of protocol deviations that might significantly affect the reliability of the study data or that might significantly affect a subject's safety. An important deviation can include nonadherence to inclusion or exclusion criteria or nonadherence to guidance from regulatory authorities including ICH E6(R2) guidelines.

Protocol deviations will be documented by the clinical monitor throughout the course of monitoring visits. The investigator will be notified of deviations in writing by the monitor. The Ethics Committee should be notified of all protocol deviations, if appropriate, in a timely manner.

10.2.3.3 Study Termination

Although the Sponsor has every intention of completing the study, they reserve the right to discontinue it at any time for clinical or administrative reasons.

The end of the study is defined as the last visit or last health status follow-up for the last subject discharged from the study (including the EOS visit and any additional long-term follow-up). Any additional long-term follow-up that is required for monitoring of the resolution of an AE or finding may be appended to the clinical study report.

10.2.3.4 Final Report

Regardless of whether the study is completed or prematurely terminated, the Sponsor will ensure that clinical study reports are prepared and provided to the appropriate regulatory agency(ies) as required by the applicable regulatory requirement(s). The Sponsor will also ensure that clinical study reports in marketing applications meet the standards of the ICH harmonised tripartite guideline E3: Structure and content of clinical study reports.

Where required by applicable regulatory requirements, an investigator signatory will be identified for the approval of the clinical study report. The investigator will be provided reasonable access to statistical tables, figures, and relevant reports and will have the opportunity to review the complete study results.

Upon completion of the clinical study report, the investigator(s) will be provided with the final approved clinical study report, as appropriate.

10.3 Appendix 3: Glossary of Terms

Term	Definition
Adequate contraception	Adequate contraception is defined as consistent and correct use of a Food and Drug Administration-approved contraceptive method in accordance with the product label, such as the following:<br>• Barrier method (condoms, diaphragm, or cervical cap) with spermicide<br>• Intrauterine device<br>• Hormonal contraceptive in the form of a pill or patch<br>• Medroxyprogesterone injection (Depo-Provera®)<br>• Etonogestrel implant (Nexplanon®)<br>• Sterilization of a female subject's monogamous male partner before entry into the study<br>Note: Periodic abstinence (eg, calendar, ovulation, symptothermal, and post-ovulation methods) and withdrawal are not acceptable methods of contraception.
Grade 1 laboratory abnormality with potential clinical significance	A grade 1 laboratory parameter that cannot be explained or that is judged by the investigator to be potentially clinically significant. A list of laboratory-specific normal ranges and associated toxicity grades is provided in Section 10.4.
Grade 1 laboratory abnormality without potential clinical significance	A grade 1 laboratory parameter that can be explained by a condition that is not related to study drug infusion and does not increase the risk for an adverse outcome from study drug infusion. A list of laboratory-specific normal ranges and associated toxicity grades is provided in Section 10.4.
Menopause	Natural menopause is defined as the permanent cessation of menstrual periods, determined retrospectively after a woman has experienced 12 months of amenorrhea without any other obvious pathological or physiological cause. Menopause occurs at a median age of 51.4 years.
Protocol amendment	The International Council for Harmonisation (ICH) defines a protocol amendment as "A written description of a change(s) to or formal clarification of a protocol." It may include a change that affects the safety of subjects, scope of the investigation, study design, or scientific integrity of the study.
Protocol administrative change	A protocol administrative change addresses changes only to logistical or administrative aspects of the study.

10.4 Appendix 4: Toxicity Grading Scale Tables

The toxicity grading scales for clinical abnormalities that will be utilized are included in CTCAE v5, which will be used for all toxicity grading for AEs and can be referenced here:

https://ctep.cancer.gov/protocoldevelopment/electronic_applications/docs/ctcae_v5_quick_reference_5x7.pdf.

For vital sign toxicity grading, the DHHS 2007 "toxicity grading scale for healthy adult and adolescent volunteers enrolled in preventative vaccine clinical trials" will be used as reference (tables for clinical abnormalities). This will be used for vital signs only and is presented in Table 10-1.

The toxicity grading scales for laboratory abnormalities are presented in Table 10-2, Table 10-3, and Table 10-4. Note that for laboratory abnormalities, grading only occurs if the values reside outside the normal values established by the clinical laboratory. For study-specific laboratory normal ranges and associated toxicity grades, refer to the laboratory manual.

Table 10-1: Tables for Clinical Abnormalities - Vital Signs Only

Vital Signs[(a)]	Mild (Grade 1)	Moderate (Grade 2)	Severe (Grade 3)	Potentially Life Threatening (Grade 4)
Fever (°C)[(b)]<br>(°F)[(b)]	38.0 - 38.4<br>100.4 - 101.1	38.5 - 38.9<br>101.2 - 102.0	39.0 - 40.0<br>102.1 - 104.0	> 40<br>> 104
Tachycardia (beats per minute)	101 - 115	116 - 130	> 130	ER visit or hospitalization for arrhythmia
Bradycardia (beats per minute)[(c)]	50 - 54	45 - 49	< 45	ER visit or hospitalization for arrhythmia
Hypertension (systolic) (mm Hg)	141 - 150	151 - 155	> 155	ER visit or hospitalization for malignant hypertension
Hypertension (diastolic) (mm Hg)	91 - 95	96 - 100	> 100	ER visit or hospitalization for malignant hypertension
Hypotension (systolic) (mm Hg)	85 -89	80 - 84	< 80	ER visit or hospitalization for hypotensive shock
Respiratory rate (breaths per minute)	17 - 20	21 - 25	> 25	Intubation

Abbreviation: ER, emergency room.

a. Subject should be at rest for all vital sign measurements.

b. Oral temperature; no recent hot or cold beverages or smoking.

c. When resting heart rate is between 60 - 100 beats per minute. Use clinical judgment when characterizing bradycardia among some healthy subject populations, for example, conditioned athletes.

Source: Guidance for industry - Toxicity grading scale for heathy adult and adolescent volunteers enrolled in preventative vaccine clinical trials; Tables for clinical abnormalities (DHHS 2007).

Table 10-2: Study-Specific Normal Ranges and Associated Laboratory Abnormalities

Serum Chemistry[a]	Mild (Grade 1)	Moderate (Grade 2)	Severe (Grade 3)	Potentially Life Threatening (Grade 4)[b]
Sodium – hyponatremia (mEq/L)	132 - 134	130 - 131	125 - 129	< 125
Sodium – hypernatremia (mEq/L)	144 -145	146 - 147	148 - 150	> 150
Potassium – hyperkalemia (mEq/L)	5.1 - 5.2	5.3 - 5.4	5.5 - 5.6	> 5.6
Potassium – hypokalemia (mEq/L)	3.5 - 3.6	3.3 - 3.4	3.1 - 3.2	< 3.1
Glucose – hypoglycemia (mg/dL)	65 - 69	55 - 64	45 - 54	< 45
Glucose – hyperglycemia<br>Fasting (mg/dL)<br>Random (mg/dL)	 100 - 110<br>110 - 125	 111 - 125<br>126 - 200	 > 125<br>> 200	Insulin requirements or hyperosmolar coma
Blood urea nitrogen (mg/dL)	23 - 26	27 - 31	> 31	Requires dialysis
Creatinine (mg/dL)	1.5 - 1.7	1.8 - 2.0	2.1 - 2.5	> 2.5 or requires dialysis
Calcium – hypocalcemia (mg/dL)	8.0 - 8.4	7.5 - 7.9	7.0 - 7.4	< 7.0
Calcium – hypercalcemia (mg/dL)	10.5 - 11.0	11.1 - 11.5	11.6 - 12.0	> 12.0
Magnesium – hypomagnesemia (mg/dL)	1.3 - 1.5	1.1 - 1.2	0.9 - 1.0	< 0.9
Phosphorous – hypophosphatemia (mg/dL)	2.3 - 2.5	2.0 - 2.2	1.6 - 1.9	< 1.6
Albumin – hypoalbuminemia (g/dL)	2.8 - 3.1	2.5 - 2.7	< 2.5	–
Total Protein – hypoproteinemia (g/dL)	5.5 - 6.0	5.0 - 5.4	< 5.0	–
Alkaline phosphate; increase by factor	1.1 - 2.0 × ULN	2.1 - 3.0 × ULN	3.1 - 10 × ULN	> 10 × ULN
Liver function tests –ALT and AST; increase by factor	1.1 - 2.5 × ULN	2.6 - 5.0 × ULN	5.1 - 10 × ULN	> 10 × ULN
Bilirubin – when accompanied by any increase in liver function test; increase by factor	1.1 - 1.25 × ULN	1.26 - 1.5 × ULN	1.51 - 1.75 × ULN	> 1.75 × ULN
Bilirubin – when liver function test is normal; increase by factor	1.1 - 1.5 × ULN	1.6 - 2.0 × ULN	2.0 - 3.0 × ULN	> 3.0 × ULN
Cholesterol	201 - 210	211 - 225	> 226	–

Abbreviations: ALT, alanine aminotransferase; AST, aspartate aminotransferase; ULN, upper limits of normal.

[a.] The laboratory values provided in the tables serve as guidelines and are dependent upon institutional normal parameters. Normal reference ranges in SI units (CBER Table for Laboratory Abnormalities in SI Units) are provided in a Study-Specific Laboratory Manual.

[b.] The clinical signs or symptoms associated with laboratory abnormalities might result in characterization of the laboratory abnormalities as potentially life threatening (Grade 4). For example, a low sodium value that falls within a Grade 3 parameter (125 - 129 mEq/L) should be recorded as a Grade 4 hyponatremia event if the subject had a new seizure associated with the low sodium value.

Source: Guidance for industry – Toxicity grading scale for heathy adult and adolescent volunteers enrolled in preventative vaccine clinical trials; Tables for laboratory abnormalities (CBER 2007).

Table 10-3: Study-Specific Normal Ranges - Hematology

Hematology[(a)]	Mild (Grade 1)	Moderate (Grade 2)	Severe (Grade 3)	Potentially Life Threatening (Grade 4)
Hemoglobin (female) (g/dL)	11.0 - 12.0	9.5 - 10.9	8.0 - 9.4	< 8.0
Hemoglobin (female) change from Baseline value (g/dL)	Any decrease - 1.5	1.6 - 2.0	2.1 - 5.0	> 5.0
Hemoglobin (male) (g/dL)	12.5 - 13.5	10.5 - 12.4	8.5 - 10.4	< 8.5
Hemoglobin (male) change from Baseline value (g/dL)	Any decrease - 1.5	1.6 - 2.0	2.1 - 5.0	> 5.0
WBC increase (cell/mm^3)	10 800 - 15 000	15 001 - 20 000	20 001 - 25 000	> 25 000
WBC decrease (cell/mm^3)	2500 - 3500	1500 - 2499	1000 - 1499	< 1000
Lymphocytes decrease (cell/mm^3)	750 - 1000	500 - 749	250 - 499	< 250
Neutrophils decrease (cell/mm^3)	1500 - 2000	1000 - 1499	500 - 999	< 500
Eosinophils (cell/mm^3)	650 - 1500	1501 - 5000	> 5000	Hypereosinophilic
Platelets decreased (cell/mm^3)	125 000 - 140 000	100 000 - 124 000	25 000 - 99 000	< 25 000
PT; increase by factor	1.0 - 1.10 × ULN	1.11 - 1.20 × ULN	1.21 - 1.25 × ULN	> 1.25 × ULN
PTT; increase by factor	1.0 - 1.2 × ULN	1.21 - 1.4 × ULN	1.41 - 1.5 × ULN	> 1.5 × ULN
Fibrinogen increase (mg/dL)	400 - 500	501 - 600	> 600	–
Fibrinogen decrease (mg/dL)	150 - 200	125 - 149	100 - 124	< 100 or associated with gross bleeding or disseminated intravascular coagulation

Abbreviations: PT, prothrombin time; PTT, partial thromboplastin time; ULN, upper limit of the normal range; WBC, white blood cell.

a. The laboratory values provided in the tables serve as guidelines and are dependent upon institutional normal parameters. Normal reference ranges in SI units (CBER Table for Laboratory Abnormalities in SI Units) are provided in a Study-Specific Laboratory Manual.

Source: Guidance for industry – Toxicity grading scale for heathy adult and adolescent volunteers enrolled in preventative vaccine clinical trials; Tables for laboratory abnormalities (CBER 2007).

Table 10-4: Study-Specific Normal Ranges - Urine

Urine[a]	Mild (Grade 1)	Moderate (Grade 2)	Severe (Grade 3)	Potentially Life Threatening (Grade 4)
Protein	Trace	1+	2+	Hospitalization or dialysis
Glucose	Trace	1+	2+	Hospitalization for hyperglycemia
Blood (microscopic) – red blood cells per high power field (rbc/hpf)	1 – 10	11 – 50	> 50 and/or gross blood	Hospitalization or packed red blood cells transfusion

a. The laboratory values provided in the tables serve as guidelines and are dependent upon institutional normal parameters. Normal reference ranges in SI units (CBER Table for Laboratory Abnormalities in SI Units) are provided in a Study-Specific Laboratory Manual.

Source: Guidance for industry – Toxicity grading scale for heathy adult and adolescent volunteers enrolled in preventative vaccine clinical trials; Tables for laboratory abnormalities (CBER 2007).

10.5 Appendix 5: Second Symposium on the Definition and Management of Anaphylaxis Summary Report - Second National Institute of Allergy and Infectious Disease/Food Allergy and Anaphylaxis Network Symposium

Second symposium on the definition and management of anaphylaxis: Summary report-Second National Institute of Allergy and Infectious Disease/Food Allergy and Anaphylaxis Network symposium can be accessed at the following website link: https://www.jacionline.org/article/S0091-6749%2805%2902723-5/pdf.

Clinical criteria for diagnosing anaphylaxis are presented in Table 10-5.

Deleted:

Table 10-5: Clinical Criteria for Diagnosing Anaphylaxis

**Anaphylaxis is highly likely when any <u>one</u> of the following 3 criteria are fulfilled:**
1. Acute onset of an illness (minutes to several hours) with involvement of the skin, mucosal tissue, or both (eg, generalized hives, pruritus or flushing, swollen lips-tongue-uvula)<br>*AND AT LEAST ONE OF THE FOLLOWING:*<br>a. Respiratory compromise (eg, dyspnea, wheeze-bronchospasm, stridor, reduced PEF, hypoxemia)<br>b. Reduced BP or associated symptoms of end-organ dysfunction (eg, hypotonia [collapse], syncope, incontinence)<br>2. Two or more of the following that occur rapidly after exposure to a likely allergen for that patient (minutes to several hours):<br>a. Involvement of the skin-mucosal tissue (eg, generalized hives, itch-flush, swollen lips-tongue-uvula)<br>b. Respiratory compromise (eg, dyspnea, wheeze-bronchospasm, stridor, reduced PEF, hypoxemia)<br>c. Reduced BP or associated symptoms (eg, hypotonia [collapse], syncope, incontinence)<br>d. Persistent gastrointestinal symptoms (eg, crampy abdominal pain, vomiting)<br>3. Reduced BP after exposure to *<u>known</u> allergen for that patient* (minutes to several hours):<br>a. Infants and children: low systolic BP (age specific) or greater than 30% decrease in systolic BP(Error! Reference source not found.)<br>b. Adults: systolic BP of less than 90 mm Hg or greater than 30% decrease from that person's baseline

Abbreviations: BP, blood pressure; PEF, peak expiratory flow.

Low systolic blood pressure for children is defined as less than 70 mm Hg from 1 month to 1 year, less than (70 mm Hg + [2 × age]) from 1 to 10 years, and less than 90 m

Delete
