# Supplementary material for: A phase 1 trial of lipid-encapsulated mRNA encoding a monoclonal antibody with neutralizing activity against Chikungunya virus
Source: Nat Med. 2021 Dec 9;27(12):2224–33. doi: 10.1038/s41591-021-01573-6 (PMC8674127; doi:10.1038/s41591-021-01573-6)
Supplement: Supplementary file 2 — Reporting Summary [file 41591_2021_1573_MOESM2_ESM.pdf]

nature research

Corresponding author(s): Allison August

Last updated by author(s): Jun 29, 2021

Reporting Summary

Nature Research wishes to improve the reproducibility of the work that we publish. This form provides structure for consistency and transparency in reporting. For further information on Nature Research policies, see our Editorial Policies and the Editorial Policy Checklist.

Statistics

For all statistical analyses, confirm that the following items are present in the figure legend, table legend, main text, or Methods section.

n/a	Confirmed	
☐	☒	The exact sample size (*n*) for each experimental group/condition, given as a discrete number and unit of measurement
☒	☐	A statement on whether measurements were taken from distinct samples or whether the same sample was measured repeatedly
☒	☐	The statistical test(s) used AND whether they are one- or two-sided<br>*Only common tests should be described solely by name; describe more complex techniques in the Methods section.*
☒	☐	A description of all covariates tested
☒	☐	A description of any assumptions or corrections, such as tests of normality and adjustment for multiple comparisons
☐	☒	A full description of the statistical parameters including central tendency (e.g. means) or other basic estimates (e.g. regression coefficient) AND variation (e.g. standard deviation) or associated estimates of uncertainty (e.g. confidence intervals)
☒	☐	For null hypothesis testing, the test statistic (e.g. *F*, *t*, *r*) with confidence intervals, effect sizes, degrees of freedom and *P* value noted<br>*Give P values as exact values whenever suitable.*
☒	☐	For Bayesian analysis, information on the choice of priors and Markov chain Monte Carlo settings
☒	☐	For hierarchical and complex designs, identification of the appropriate level for tests and full reporting of outcomes
☒	☐	Estimates of effect sizes (e.g. Cohen's *d*, Pearson's *r*), indicating how they were calculated

Our web collection on statistics for biologists contains articles on many of the points above.

Software and code

Policy information about availability of computer code

Data collection	*Provide a description of all commercial, open source and custom code used to collect the data in this study, specifying the version used OR state that no software was used.*
Data analysis	Parameters for PK and PD were derived using non-compartmental methods with Phoenix® WinNonlin® (Certara USA Inc., Princeton, New Jersey) Version 6.4 or higher or SAS Version 9.3 or higher (SAS Institute Inc., Cary, North Carolina).Vital signs were graded using the toxicity grading scale for healthy adult and adolescent volunteers enrolled in preventative vaccine clinical trials tables for clinical abnormalities (DHHS 2007). AEs were assessed according to the CTCAE v5 toxicity grading scale.

For manuscripts utilizing custom algorithms or software that are central to the research but not yet described in published literature, software must be made available to editors and reviewers. We strongly encourage code deposition in a community repository (e.g. GitHub). See the Nature Research guidelines for submitting code & software for further information.

Data

Policy information about availability of data

All manuscripts must include a data availability statement. This statement should provide the following information, where applicable:

- Accession codes, unique identifiers, or web links for publicly available datasets
- A list of figures that have associated raw data
- A description of any restrictions on data availability

Moderna, Inc. is committed to sharing, access to patient-level data and supporting clinical documents from eligible studies with external researchers who provide methodologically-sound scientific proposals upon request, once the trial is complete. Such requests can be made to Moderna Inc., 200 Technology Square, Cambridge, MA 02139. A redacted protocol is available online as supplementary material to this article. Clintrials.gov.NCT03829384.

Field-specific reporting

Please select the one below that is the best fit for your research. If you are not sure, read the appropriate sections before making your selection.

☒ Life sciences ☐ Behavioural & social sciences ☐ Ecological, evolutionary & environmental sciences

For a reference copy of the document with all sections, see nature.com/documents/nr-reporting-summary-flat.pdf

Life sciences study design

All studies must disclose on these points even when the disclosure is negative.

Sample size	Per protocol, it was planned to enroll approximately 56 participants in the study, with 8 in each dose level group. No formal power calculations or hypotheses-testing were performed. The planned sample size was regarded as appropriate to evaluate the safety, PK and PD of mRNA-1944 in this first-in-human study.
Data exclusions	No data exclusions were used.
Replication	Replication is not applicable as it was not planned. This is a phase 1 FIH study in an early stage of clinical development that would progress to further assessment in a a next step trial such as phase 1a.
Randomization	Eight participants were planned to be enrolled at each dose level group with 6 participants to receive mRNA-1944 and 2 to receive placebo. In each group, mRNA-1944 was administered to 3 participants (sentinel), 1 participant at a time to assess safety prior to treating the remaining 5 participants (expansion) within each dose group. Following confirmation of acceptable safety and tolerability for all sentinel participants, enrollment was expanded (expansion) and the remaining 5 participants within each dose level group were randomly assigned to receive mRNA-1944 (0.1, 0.3 and 0.6 mg/kg) or placebo in a 3:2 ratio (active:placebo) for an overall ratio of 3:1.
Blinding	This phase 1 trial was investigator-blinded (except for sentinel participants). As this is a FIH study for mRNA-1944, a sentinel strategy was utililized. The administration of mRNA-1944 was not blinded in the case of sentinel participants to enable the assessment of safety by the IST. The IST reviewed the safety of each sentinel participant for 7days, the first 48 hours of which occurred in-house at the site, prior to dosing the next sentinel participant in each dose group. The IST then reviewed all sentinel data prior to randomly assigning the remainder of the dose cohort to treatment (3active:2placebo). Randomization numbers were assigned in a sequential, ascending manner and sealed in an envelope.

Reporting for specific materials, systems and methods

We require information from authors about some types of materials, experimental systems and methods used in many studies. Here, indicate whether each material, system or method listed is relevant to your study. If you are not sure if a list item applies to your research, read the appropriate section before selecting a response.

Materials & experimental systems

n/a	Involved in the study	
☐	☒	Antibodies
☐	☒	Eukaryotic cell lines
☒	☐	Palaeontology and archaeology
☒	☐	Animals and other organisms
☐	☒	Human research participants
☐	☒	Clinical data
☒	☐	Dual use research of concern

Methods

n/a	Involved in the study	
☒	☐	ChIP-seq
☒	☐	Flow cytometry
☒	☐	MRI-based neuroimaging

Antibodies

Antibodies used	mRNA-1944 encoded and produced the heavy and light chains of chikungunya antibody in humans
Validation	Serum antibodies produced elicited chikungunya neutralizing antibody, confirming that functional antibody was produced

Eukaryotic cell lines

Policy information about cell lines

Cell line source(s)	*State the source of each cell line used.*
Authentication	*Describe the authentication procedures for each cell line used OR declare that none of the cell lines used were authenticated.*

Mycoplasma contamination	*Confirm that all cell lines tested negative for mycoplasma contamination OR describe the results of the testing for mycoplasma contamination OR declare that the cell lines were not tested for mycoplasma contamination.*
Commonly misidentified lines (See ICLAC register)	*Name any commonly misidentified cell lines used in the study and provide a rationale for their use.*

Human research participants

Policy information about studies involving human research participants

Population characteristics	Healthy adult participants, 18 to 50 years of age. The mean age was 35 (range 20-50), 52.6% were female, 73.7% were White, 21.1% were Black or African American, and 5.3% were other.
Recruitment	Between January 10, 2019 and June 1, 2020, 38 adults were enrolled in the study, all of whom were eligible and randomized to treatment during January 22, 2019 and June 18, 2020. Participants were recruited to this dedicated Phase 1 research unit through largely three ways, (1) review of unit past participant logs for eligible individuals based on the inclusion and exclusion criteria, (2) public advertisements using social media, and (3) referrals from primary care physicians. As such, there are not believed to be any self-selection biases that would impact results.
Ethics oversight	The study was conducted at a single site in accordance with the International Council on Harmonization of Good Clinical Practice guidelines and the protocol was approved by regulatory (Food and Drug Administration) and institutional review board (Western Institutional Review Board (WIRB) 1019 39th Avenue SE Suite 120 Puyallup, WA 98374-2115)

Note that full information on the approval of the study protocol must also be provided in the manuscript.

Clinical data

Policy information about clinical studies

All manuscripts should comply with the ICMJE guidelines for publication of clinical research and a completed CONSORT checklist must be included with all submissions.

Clinical trial registration	ClinicalTrials.gov Identifier: NCT03829384
Study protocol	Protocol was provided with submission
Data collection	The study was conducted at a single site. in the US (study site in US, PPD Pharmacology Unit, Austin; 7551 Metro Center Drive, Suite 200 Austin, Texas 7874). Between January 10, 2019 and June 1, 2020, 38 adults were enrolled and randomized and treated January 22, 2019 to June 18, 2020.
Outcomes	As this was a first-in-human study for mRNA-1944, the primary objective of this study was to evaluate the safety and tolerability of escalating doses of mRNA-1944 administered via intravenous (IV) infusion in subjects 18 to 50 years of age, and the secondary objectives were to determine the PK of mRNA encoding for CHIKV24 IgG (heavy and light chain mRNA) and the ionic amino lipid (IAL), and to determine the PD of mRNA-1944 as assessed by CHIKV24 IgG.<br>Safety and tolerability were assessed by monitoring and recording of adverse effects (AEs), including serious AEs, infusion-related reactions, and AEs of special interest, prior and concomitant medication, clinical laboratory test results (hematology, coagulation, serum chemistry including liver enzymes, and urinalysis), vital sign measurements (systolic and diastolic blood pressure, heart rate, respiratory rate, and body temperature), ECG results (and cardiac enzymes when obtained per protocol), and physical examination findings.<br>Pharmacokinetic data for mRNA encoding for the CHKV24 IgG and for IAL were assessed from day 0 (pre-dose) through 28 days for participants in the safety population who had evaluable mRNA encoding for CHKV24 IgG. Pharmacodynamic data for serum CHKV24 IgG were assessed from day 0 (pre-dose) through 366 days for 28 participants on active treatment in the safety population who had evaluable mRNA encoding for CHKV24 IgG.
